# Global, regional, and national burden of kidney cancer, 1990 to 2021, and projections to 2050: A Global Burden of Disease Study 2021

**DOI:** 10.1097/MD.0000000000046059

**Published:** 2026-05-12

**Authors:** Yoon Lee, Seohyun Hong, Jaehyun Kong, Sooji Lee, Hayeon Lee, Jinseok Lee, Jiseung Kang, Damiano Pizzol, Hyeon Seok Hwang, Dong Keon Yon

**Affiliations:** a Department of Medicine, Kyung Hee University College of Medicine, Seoul, South Korea; b Center for Digital Health, Medical Science Research Institute, Kyung Hee University College of Medicine, Seoul, South Korea; c Department of Electronics and Information Convergence Engineering, Kyung Hee University, Yongin, South Korea; d Department of Biomedical Engineering, Kyung Hee University, Yongin, South Korea; e Division of Sleep Medicine, Harvard Medical School, Boston, MA; f School of Health and Environmental Science, College of Health Science, Korea University, Seoul, South Korea; g Department of Health and Safety Convergence Science, Korea University Graduate School, Seoul, South Korea; h Health Unit Eni, Maputo, Mozambique; i Health Unit Eni, San Donato Milanese, Italy; j Division of Nephrology, Department of Internal Medicine, Kyung Hee University Medical Center, Kyung Hee University College of Medicine, Seoul, South Korea; k Department of Pediatrics, Kyung Hee University Medical Center, Kyung Hee University College of Medicine, Seoul, South Korea.

**Keywords:** DALY, global, Global Burden of Disease Study, kidney cancer

## Abstract

Although the Global Burden of Disease Study (GBD) 2019 offered important insights into kidney cancer, no research has yet examined the updated GBD 2021. Therefore, utilizing the GBD 2021, this study provides updated estimates of kidney cancer burden, identifies attributable risk factors, and projects future trends through 2050. Data on the global and regional burden of kidney cancer from 1990 to 2021 were extracted from the GBD 2021, stratified by age, sex, Sociodemographic Index (SDI), and geographical region. Mortality-to-incidence ratio and annual percentage changes on mortality, disability-adjusted life year (DALY), and incidence were analyzed to assess trends of the burden. Attributable risk factors (i.e., tobacco use, high body mass index, and occupational risks) were examined. Additionally, future mortality and DALYs up to 2050 were forecasted by integrating independent drivers, with analyses conducted under a baseline scenario and an improved scenario with enhanced behavioral and metabolic risk factors. In 2021, kidney cancer caused 161.19 thousand (95% uncertainty interval, 150.32–169.35) deaths, 4.02 million (3.81–4.25) DALYs, 387.83 thousand (365.36–406.64) incident cases, and 0.42 (0.39–0.46) mortality-to-incidence ratio globally. While the absolute burden of kidney cancer increased from 1990 to 2021, age-standardized rates decreased. The burden was higher in males, older populations, and regions with higher SDI, although recent declines in these regions contrast with rising burden in lower SDI regions. High body mass index was the leading risk factor, particularly in females, while tobacco was more prominent in males. By 2050, the global age-standardized DALY rate is projected to rise slightly to 47.72 (35.89–63.05) per 100,000. However, under a scenario with improved behavioral and metabolic risks, it is expected to decline to 35.86 (26.71–48.11), except in South Asia and Sub-Saharan Africa. Addressing sociodemographic disparities in kidney cancer burden and strengthening global efforts are essential. Managing behavioral and metabolic risks could reduce the kidney cancer burden.

## 1. Introduction

Kidney cancer is globally recognized as a lethal disease, with over 400,000 new cases estimated to be diagnosed annually.^[1]^ Ranking as the 14th most prevalent cancer, kidney cancer presents complex epidemiological patterns and imposes a significant burden on global healthcare systems.^[1,2]^ It predominantly affects males and older populations, with well-established risk factors including a high body mass index (BMI), smoking, and chronic kidney disease.^[3]^ Europe and North America have reported higher incidence rates, while low-income regions are anticipated to experience a substantial increase in the coming years.^[2,4]^

Although these differences in demographic groups have been well-documented, kidney cancer remains under-researched and its incidence continues to increase, contributing substantially to the global burden.^[4,5]^ Nevertheless, the updated Global Burden of Disease Study (GBD) 2021 has yet to be examined, leaving critical knowledge gaps in this growing global burden unaddressed. Additionally, responses in low-income countries are restricted by research gaps, socioeconomic inequalities, and limited access to high-quality healthcare.^[6,7]^ These disparities may lead to premature deaths in low- and middle-income countries.^[8]^ Therefore, this study utilizes the GBD 2021 to provide a comprehensive and up-to-date analysis, examining data by sex, age, geographical region, Sociodemographical Index (SDI), and attributable risk factors. By avoiding an exclusive focus on specific groups, such as high-income regions, our approach assesses the recent global burden and identifies areas where significant knowledge gaps persist.

Moreover, given the scarcity of previous research on the forecasted burden of kidney cancer, updated forward-looking analyses are essential.^[9]^ By focusing on populations that are projected to be more vulnerable in the future, we aim to analyze the future kidney cancer burden, for assisting policymakers and researchers in developing effective alternatives.^[10]^ Furthermore, this study incorporates regional factors as predictive indicators to forecast kidney cancer trends by 2050, thus promoting equitable and sustainable healthcare responses.

## 2. Methods

### 2.1. Study data and case definition

This study utilized data on global, regional, and national kidney cancer burdens from the GBD 2021. The GBD provides a comprehensive assessment of 371 diseases and conditions across 204 countries and territories from 1990 to 2021 (Table S1, Supplemental Digital Content, https://links.lww.com/MD/Q720). Key health metrics, including cause-specific incidence, prevalence, mortality, years of life lost, years lived with disability, and disability-adjusted life years (DALYs) were assessed. Analyses were conducted at the country level and stratified by GBD regions and SDI levels. SDI is a composite measure of social and economic factors affecting health outcomes across regions. It is calculated as the geometric mean of 3 indices, total fertility rate under 25 years of age, average educational attainment, and lagged distributive income per capita. Additionally, future projections of the kidney cancer burden through 2050 were obtained. The GBD 2021 follows the Guidelines for Accurate and Transparent Health Estimates Reporting to ensure methodological transparency.

Kidney cancer was defined based on the International Classification of Diseases (ICD) codes. Specifically, kidney cancer was classified under ICD-10 as Z80.43 and Z85.47 to Z85.48, and under ICD-9 as V10.47 to V10.48 and V16.43.^[11]^ Data sources included vital registration, verbal autopsy, and cancer registry data within the GBD framework. For kidney cancer, 3 prevalence sources, 3499 incidence sources, 4128 death sources, and 7215 all measure sources were analyzed.^[12]^ All statistical analyses were conducted via Python (version 3.10.4; Python Software Foundation, Wilmington) and R software (version 4.3.2; R Foundation, Vienna, Austria).

### 2.2. Estimating the burden of kidney cancer

Cancer mortality estimates were produced using the Cause of Death Ensemble Model, integrating crude mortality estimates with additional data from vital registration, verbal autopsy sources, and other relevant covariates.^[12]^ Furthermore, to account for heterogeneity in regional data quality, the GBD 2021 framework applies standardized data cleaning, redistribution of ill-defined codes, and ensemble modeling. The Cause of Death Ensemble Model strategy applied 4 model classes, including linear mixed effects regression and spatiotemporal Gaussian process regression models. Covariates were selected through a literature review and categorized into 3 levels based on evidence strength. The included covariates for kidney cancer were cigarettes per capita, cumulative cigarettes, mean BMI, log-transformed summary exposure value scalar, alcohol consumption, diabetes prevalence, systolic blood pressure, Healthcare Access and Quality index, education, lagged distributive income, and SDI. Model performance was evaluated through repeated out-of-sample cross-validation, in which subsets of data were predicted by candidate models. Based on predictive validity, models with higher out-of-sample predictive performance received higher weights compared to those with poorer performance. Final estimates were generated from a weighted ensemble of these models, ensuring that results reflected a balance of predictive performance across regions and time periods. Additional detailed methods can be found elsewhere.^[13]^

Mortality-to-incidence ratios (MIRs) were derived from registries containing both incidence and mortality data to enhance data availability in the disease-model-Bayesian meta-regression (DisMod-MR) 2.1 modeling framework.^[12]^ Because MIRs can occasionally exceed 1.0, especially in older age groups, the GBD framework applies upper cap thresholds defined as the 95th percentile of the dataset by age group. These upper caps constrain MIRs to plausible maximum levels while still permitting MIR above 1.0 when supported by data. Further details on upper cap values of MIR are described elsewhere.^[14]^ Kidney cancer incidence was then directly estimated from kidney cancer mortality estimates using MIRs. Initially, incidence and mortality data were processed and aligned by cancer type, age, sex, year, and location to compute crude MIRs. MIRs were refined using spatiotemporal Gaussian process regression, incorporating the Healthcare Access and Quality index, age, and sex as covariates. Subsequently, final mortality estimates were divided by the modeled MIR estimates. The 95% uncertainty interval (UI) was generated with 1000 draws.^[12]^ Uncertainty in the MIR was assumed to be independent of uncertainty in the estimated mortality.

### 2.3. Annual percentage change

Temporal trends in the burden of kidney cancer were analyzed using the annual percentage change (APC). The formula for the regression model is as follows.


$$\begin{array}{l} y=\;\alpha +\beta x+\;\varepsilon \end{array}$$

*y* represents the natural logarithm of the burden of kidney cancer, *x* is the year, and β is the regression coefficient. Age-standardized mortality rates (ASMR), age-standardized incidence rates (ASIR), and age-standardized DALYs rates (ASDR) were used as the burden of kidney cancer. APC was calculated using the following formula. A *P* value ≤ .05 was considered statistically significant using a 2-sided test.^[15]^


$$\begin{array}{l} APC\;\left(\%\right)=(\exp\left(\beta \right)-1)\;\times 100 \end{array}$$

### 2.4. Risk factors

GBD 2021 incorporated risk–outcome pairs supported by strong evidence to quantify the proportion of disease burden attributable to specific risk factors.^[13]^ The relative risk of the outcome was based on exposure levels, the prevalence of exposures, and the theoretical minimum risk exposure level. The risk-deleted kidney cancer mortality rate is calculated by subtracting the population attributable fraction for a specific risk factor from the observed kidney cancer mortality rate. Through this, the contribution of each risk factor and the number of deaths that could be prevented if exposure to these factors were minimized were estimated. Our study examined the burden of kidney cancer attributable to tobacco use, high BMI, and occupational risks, classified as level 2 risk factors in the GBD. Specifically, disaggregating to level 3 risk factor classification, tobacco use was assessed solely through smoking, while occupational risks included occupational carcinogens and trichloroethylene.

### 2.5. Projections to 2050

Kidney cancer mortality and DALYs were forecasted up to 2050 by integrating independent drivers of health, sociodemographic determinants, and key interventions.^[16]^ SDI, vaccine coverages, and anti-retroviral therapy were included. The forecasting framework incorporated historical trends, recency-weighted changes, and external shocks. First, future risk factor trajectories were modeled using summary exposure values to assess population attributable fractions and cause-specific mortality scalars. A generalized ensemble modeling approach was utilized, incorporating 12 sub-models based on 2 primary methodologies: annualized rate of change-based models and a 2-stage spline model employing a meta-regression-Bayesian, regularized, trimmed model. An ensemble modeling approach was adopted for mediation in mediator summary exposure value computation to prevent overestimation of risk contributions. Sub-model weights were determined through out-of-sample predictive validity experiments, training models in 1990 to 2009 data, and validating in 2010 to 2019 data using root mean squared error. Final forecasts up to 2050 were generated using 500 draws per model.

Cause-specific mortality estimates were obtained using a log-linear model, modeled as a function of the SDI, temporal factors, other cause-specific covariates, and the risk factor scalars. DALYs and mortality forecasts were generated under 2 scenarios, the reference scenario and an alternative scenario. The alternative scenario, termed as the improved behavioral and metabolic risks scenario, assumes the complete elimination of smoking, dietary risks, and high BMI in adults by 2050, with a linear decline to 0 starting in 2023.^[16,17]^ Additionally, no new smoker initiation was assumed after 2022.

## 3. Results

### 3.1. Global trends in kidney cancer burdens

In 2021, there were 387.83 thousand (95% UI, 365.36–406.64) incident cases and 77.42 thousand (74.81–79.70) deaths of kidney cancer globally. Global ASIR increased from 3.89 (95% UI, 3.75–3.99) in 1990 to 4.52 (4.26–4.75) in 2021, while the ASMR decreased from 1.99 (1.91–2.06) to 1.91 (1.78–2.01) per 100,000 population (Table 1). Between 1990 and 2021, the overall burden of kidney cancer increased in absolute terms, while age-standardized rates (ASR) and MIR decreased. Total DALYs increased from 2.29 million (95% UI, 2.19–2.39) in 1990 to 4.02 million (3.81–4.25) in 2021, and the number of incident cases also increased from 159.77 thousand (154.83–163.93) to 387.83 thousand (365.36–406.64). However, from 2010 to 2021, ASDR and ASIR showed declining trends (APC of ASDR: −0.86 [95% UI, −0.94 to −0.78]; APC of ASIR: −0.58 [−0.70 to −0.47]) (Tables S2–S4, Supplemental Digital Content, https://links.lww.com/MD/Q720).

**Table 1:** Global counts and age-standardized rates (per 100,000 population) of incidence, mortality, and disability-adjusted life years and mortality-to-incidence ratio for kidney cancer in 1990 and 2021.

	Both (95% UI)		Males (95% UI)		Females (95% UI)	
	1990	2021	1990	2021	1990	2021
Incidence						
Count	159,774.29 (154,831.25–163,926.28)	387,828.72 (365,359.71–406,635.25)	96,350.35 (93,531.34–99,172.95)	252,589.19 (237,746.50–268,143.49)	63,423.94 (60,062.48–66,261.42)	135,239.53 (124,078.89–144,234.80)
ASR, per 100,000 population	3.89 (3.75–3.99)	4.52 (4.26–4.75)	5.09 (4.93–5.23)	6.26 (5.88–6.64)	2.87 (2.73–2.99)	3.00 (2.76–3.20)
Mortality						
Count	77,420.69 (74,807.39–79,691.27)	161,194.54 (150,317.57–169,348.28)	47,898.42 (46,249.71–49,525.03)	106,543.65 (99,856.60–112,786.67)	29,522.27 (27,711.13–31,094.16)	54,650.89 (48,631.49–58,643.21)
ASR, per 100,000 population	1.99 (1.91–2.06)	1.91 (1.78–2.01)	2.75 (2.66–2.84)	2.79 (2.61–2.95)	1.39 (1.30–1.46)	1.19 (1.06–1.28)
DALYs						
Count	2291,994.78 (2,190,460.43–2,385,797.84)	4016,362.12 (3,806,832.21–4246,783.03)	1431,456.95 (1368,620.90–1509,811.37)	2738,077.26 (2581,071.77–2925,663.38)	860,537.84 (790,202.97–939,488.08)	1278,284.86 (1176,214.64–1371,879.89)
ASR, per 100,000 population	53.02 (50.96–54.93)	47.33 (44.76–50.07)	70.83 (68.10–73.84)	67.79 (63.83–72.46)	37.41 (34.74–40.23)	28.91 (26.54–31.21)
MIR	0.51 (0.49–0.54)	0.42 (0.39–0.46)	0.54 (0.52–0.57)	0.45 (0.41–0.49)	0.48 (0.45–0.52)	0.40 (0.35–0.44)

ASR = age-standardized rate, DALYs = disability-adjusted life years, MIR = mortality-to-incidence ratio, UI = uncertainty interval.

In 2021, the burden of kidney cancer was significantly higher in males than in females (Table 1). For males, the number of deaths was 106.54 thousand (95% UI, 99.86–112.79) and incident cases were 252.59 thousand (237.75–268.14). Females exhibited lower values in both metrics, with 54.65 thousand deaths (95% UI, 48.63–58.64) and 135.24 thousand incident cases (124.08–144.23). The MIR was 0.45 (95% UI, 0.41–0.49) in males and 0.40 (0.35–0.44) in females, highlighting a greater burden of kidney cancer in males.

### 3.2. Kidney cancer burden by age

**Figure 1.**
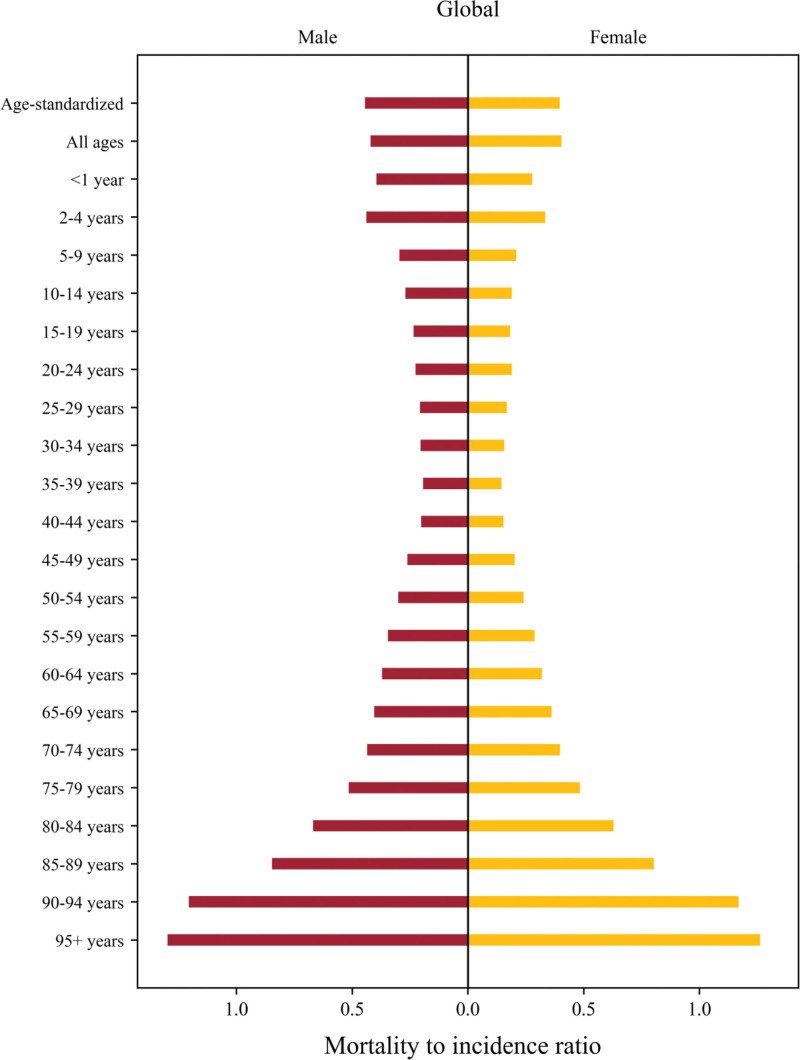
Global mortality-to-incidence ratio of kidney cancer stratified by sex and age in 2021.

Figure S1 and Tables S5 to S10, Supplemental Digital Content, https://links.lww.com/MD/Q720 present age-standardized burden rates and MIR by age groups. At the global level in 2021, the mortality and DALY rates were highest in 90 to 94 years group in males and in 95+ years group in females. The DALY rates of high SDI region were highest in the 90 to 94 years group for males (867.90 [95% UI, 722.93–950.95]) and in the 95+ years group for females (418.76 [293.92–493.24]). In contrast, in low SDI region, the 75 to 79 years group was highest for both males (140.08 [95% UI, 104.10–171.17]) and females (71.30 [55.14–90.08]). For both sexes, low SDI region had higher DALY rates in the 2 to 4 years group (males: 62.11 [95% UI, 37.54–89.10]; females: 50.54 [31.54–71.75]) than in high SDI region (males: 4.77 [95% UI, 4.14–5.48]; females: 5.14 [4.54–5.84]). Moreover, as shown in Figure 1 and Figure S2 to S6, Supplemental Digital Content, https://links.lww.com/MD/Q720 the MIR was higher in lower SDI regions across all age groups. In the 2 to 4 years group, the MIR in high SDI region was 0.14 (95% UI, 0.12–0.17) in males and 0.11 (0.09–0.13) in females. In low SDI region, it was significantly higher with 0.55 (95% UI, 0.29–1.04) in males and 0.46 (0.25–0.85) in females. The age group for both sexes in which MIR exceeded 1.00 was 90 to 94 years in higher SDI regions, whereas in lower SDI regions, this threshold was reached at an earlier age (80–84 years).

### 3.3. Kidney cancer burden by regions

**Figure 2.**
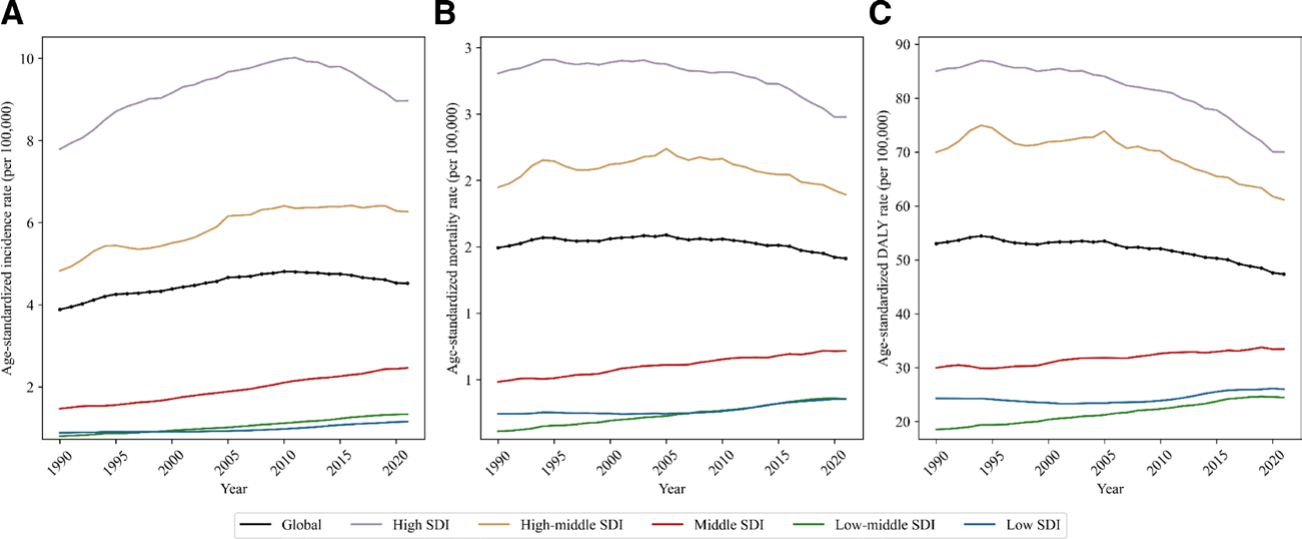
Trends of age-standardized rates for (A) incidence, (B) mortality, and (C) disability-adjusted life years of kidney cancer for both sexes, 1990 to 2021, globally and by SDI regions. SDI = Sociodemographic Index.

Figure 2 shows that higher SDI regions had higher burdens of kidney cancer (ASMR, high SDI: 2.98 [95% UI, 2.74–3.12]; low SDI: 0.85 [0.65–1.04]) in 2021. The ASIR in high SDI region (8.97 [8.46–9.33]) was more than 7 times higher than that in low SDI region (1.15 [0.87–1.40]). However, the MIR was higher in lower SDI regions (high SDI: 0.33 [95% UI, 0.31–0.36]; low SDI: 0.74 [0.53–1.03]) (Table S11, Supplemental Digital Content, https://links.lww.com/MD/Q720). Furthermore, in recent years, the burdens have increased in lower SDI regions and decreased in higher SDI regions. In Tables S2 to S4, Supplemental Digital Content, https://links.lww.com/MD/Q720, this increasing trend in lower SDI region and the decreasing trend in higher SDI region have been shown through the APC of ASMR and ASDR from 2010 to 2021 (low SDI, APC of ASMR: 1.10 [95% UI, 0.93–1.27]; APC of ASDR: 0.85 [0.64–1.07]; high SDI, APC of ASMR: −1.07 [−1.23 to −0.91]; APC of ASDR: −1.47 [−1.64 to −1.29]).

A similar trend was also observed across global regions. Figures S7 and S8, Supplemental Digital Content, https://links.lww.com/MD/Q720 present the global distribution of kidney cancer burdens in 1990 and 2021. High-income (e.g., Southern Latin America and Western Europe) and Central Europe, Eastern Europe, and Central Asia exhibited higher ASIR, ASMR, and ASDR values in both years, whereas South Asia and Sub-Saharan Africa showed a lower burden (Figures S9–S12, Supplemental Digital Content, https://links.lww.com/MD/Q720). However, in recent years, high-income and Central Europe, Eastern Europe, and Central Asia have shown a decreasing trend in kidney cancer burdens. In Table S4, Supplemental Digital Content, https://links.lww.com/MD/Q720, from 2010 to 2021, the ASDR showed the most significant decline in high-income (APC: −1.25 [95% UI, −1.50 to −1.01]), followed by Central Europe, Eastern Europe, and Central Asia (APC: −1.11 [−1.22 to −1.00]). In contrast, South Asia exhibited the highest increase (APC: 1.35 [95% UI, 1.19–1.50]). Additionally, Southern Sub-Saharan Africa was the only region to increase by more than 3 ranks in ASDR between 1990 and 2021 (Figure S12, Supplemental Digital Content, https://links.lww.com/MD/Q720).

### 3.4. Risk factors

Globally in 2021, Figure 3 and Figure S13, Supplemental Digital Content, https://links.lww.com/MD/Q720 illustrate that the risk factor metrics for males and females were similar in both ASMR and ASDR. High BMI was the leading risk factor for kidney cancer ASMR and ASDR for both males (ASMR: 0.53 [95% UI, 0.21–0.86] per 100,000 population; ASDR: 12.52 [5.09–20.38]) and females (ASMR: 0.26 [95% UI, 0.10–0.41]; ASDR: 5.81 [2.34–9.20]). In both rates, tobacco followed next, while occupational risks exhibited a minimal value (Tables S12–S17, Supplemental Digital Content, https://links.lww.com/MD/Q720). The ASDR for high BMI in females increased between 1990 and 2021 in most regions, except for Australasia, high-income North America, and Western Europe. The ASDR attributable to tobacco was over 8 times higher in males than in females, and the impact of tobacco among males was pronounced in high-income Asia Pacific, East Asia, and Southeast Asia, where tobacco imposed a greater burden than high BMI. These were the only 3 regions among all GBD regions where tobacco had a greater impact than high BMI. Notably, the ASDR for high BMI for both sexes combined in high-income Asia Pacific (4.74 [95% UI, 1.80–7.60]) was the lowest among high-income and Central Europe, Eastern Europe, and Central Asia super-regions, with tobacco in high-income Asia Pacific also having the lowest rates (4.26 [95% UI, 2.63–6.08]) (Tables S13–S15, Supplemental Digital Content, https://links.lww.com/MD/Q720).

**Figure 3.**
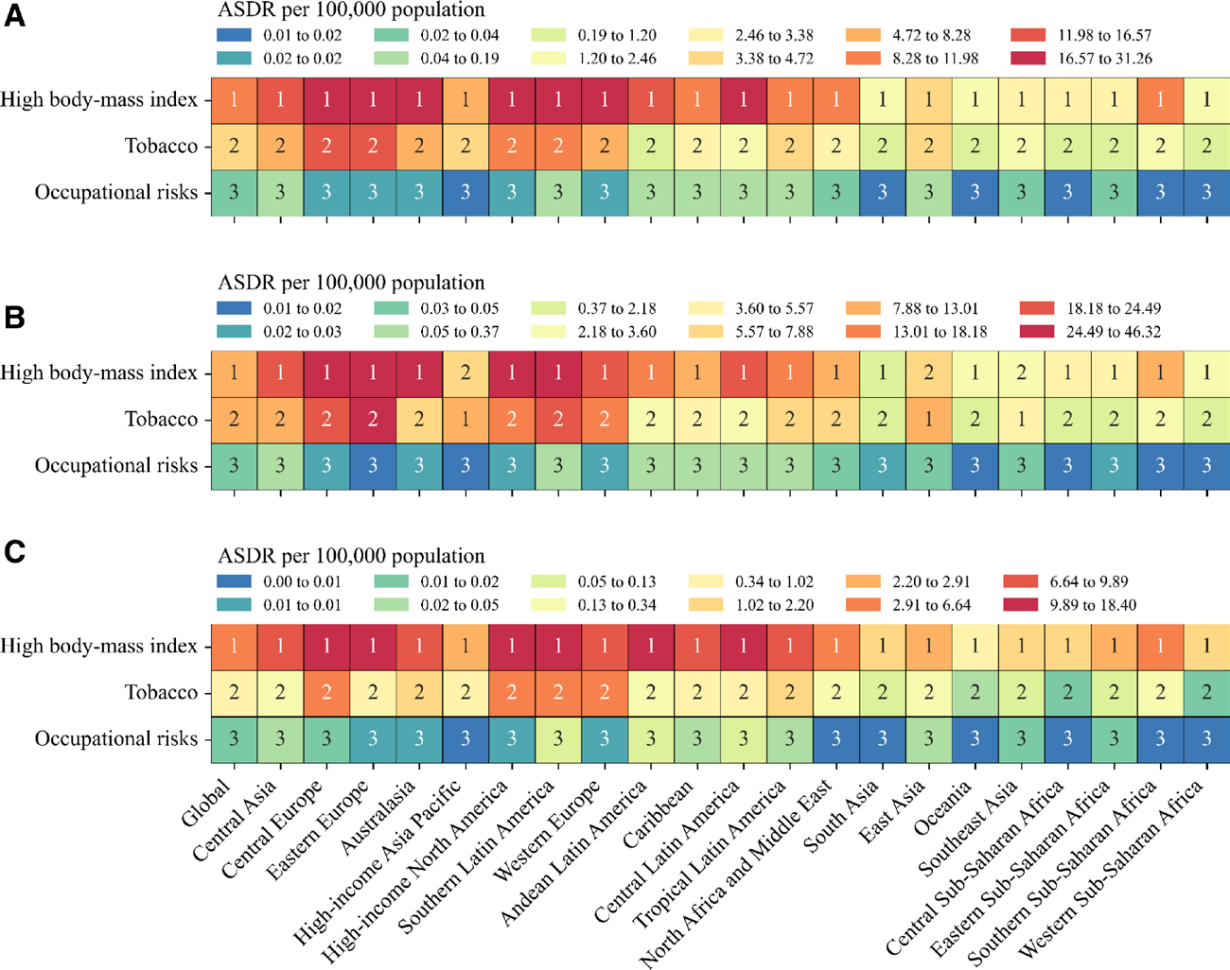
ASDR of kidney cancer attributable to high body mass index, tobacco, and occupational risks in 2021, by (A) both sexes, (B) males, and (C) females, ranked globally and by region. ASDR = age-standardized disability-adjusted life years rate.

### 3.5. Forecasts to 2050

Globally, the kidney cancer death counts are projected to more than double between 2021 and 2050, from 161.19 thousand (95% UI, 150.04–169.98) to 323.95 thousand (231.58–430.00). Total DALYs are also forecasted to increase from 4.02 million (95% UI, 3.81–4.25) to 6.85 million (4.99–9.09). However, under the improved behavioral and metabolic risks scenario, deaths and DALYs are estimated to decrease, with 282.19 thousand (95% UI, 203.13–371.35) deaths and 5.58 million (4.11–7.39) DALYs expected in 2050 (Tables S18–S21, Supplemental Digital Content, https://links.lww.com/MD/Q720). In terms of regions, the high-income region is forecasted to have the highest number of deaths (108.71 thousand, [95% UI, 87.30–130.53]) and DALYs (1.90 million [1.60–2.24]) in 2050. North Africa and Middle East, South Asia, and Sub-Saharan Africa are projected to experience a threefold increase in deaths and a twofold increase in DALYs between 2021 and 2050.

**Figure 4.**
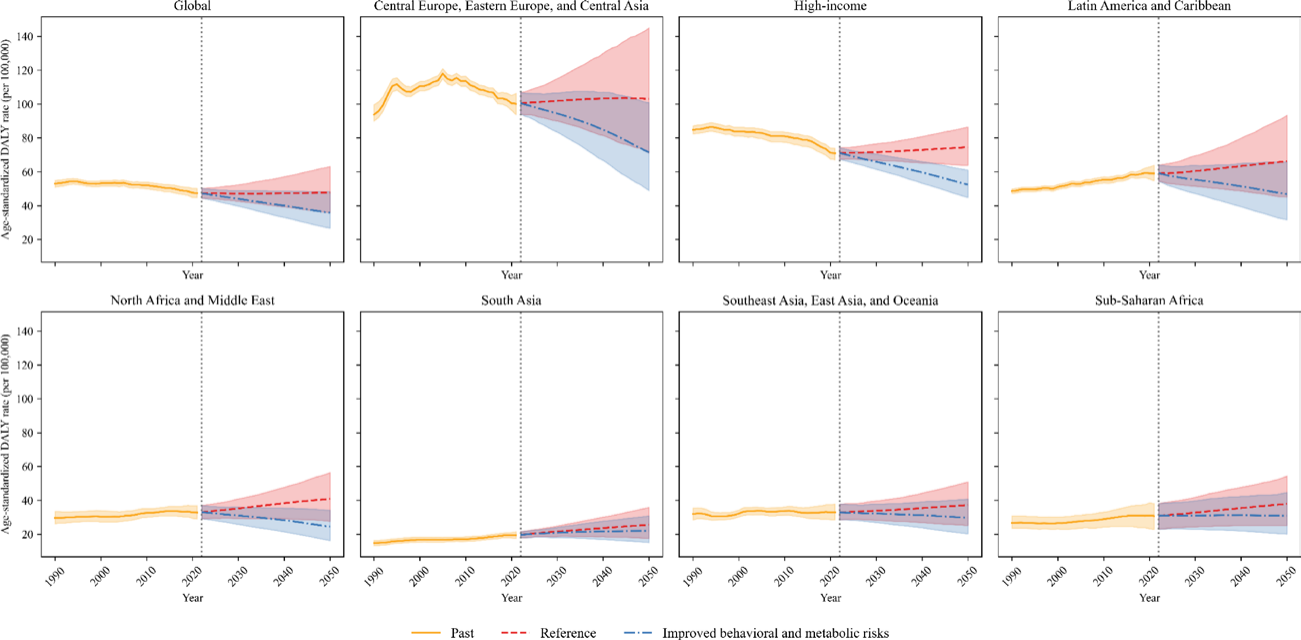
Forecasted DALYs rates (per 100,000 population) to 2050 under 2 scenarios (reference and improved behavioral and metabolic risks), globally and by regions. The shaded regions indicate the 95% uncertainty intervals, and the vertically dashed line marks the start of the forecast period in 2022. DALYs = disability-adjusted life years.

From 2021 to 2050, the ASMR and ASDR for all regions are expected to slightly increase under the reference scenario. However, under the improved scenario, 5 regions, excluding South Asia and Sub-Saharan Africa, are predicted to experience a decline in ASDR (Figure 4 and Figure S14, Supplemental Digital Content, https://links.lww.com/MD/Q720). In South Asia, all 5 countries are projected to see an increase in ASDR even under the improved scenario, with Bhutan the sharpest increase. Similarly, in Sub-Saharan Africa, the ASDR of 25 out of the 46 countries increased under the improved scenario, with Ethiopia and Uganda predicted to experience significant increases (Figures S15–S21, Supplemental Digital Content, https://links.lww.com/MD/Q720).

## 4. Discussion

### 4.1. Key findings

This study provides a comprehensive analysis of the global and regional burden of kidney cancer based on GBD 2021, with attributable risk factors and projections to 2050. From 1990 to 2021, the global number of kidney cancer burdens nearly doubled, with 387.82 thousand (95% UI, 365.36–406.64) new cases and 161.20 thousand (150.32–169.35) deaths in 2021. Despite the increase in the absolute numbers of deaths, DALY, and incidence, ASRs have decreased from 1990 to 2021. Males and older populations showed higher burdens of kidney cancer compared to females and younger populations. Although higher SDI regions have higher burdens of kidney cancer, recent trends indicate declines in these regions, in contrast to the rising burdens in lower SDI regions. Furthermore, lower SDI regions have higher MIRs of kidney cancer. High BMI is the leading risk factor for kidney cancer in both sexes, with attributable burdens increasing from 1990 to 2021. Tobacco use is the following risk factor, particularly attributable to males. In the baseline scenario, future projections indicate that mortality and DALYs from kidney cancer will slightly increase on a global scale. However, in a scenario with improved behavioral and metabolic risks, burdens are expected to decline by 2050, except in South Asia and Sub-Saharan Africa. Targeted policies, adapted to national and sociodemographic contexts, are essential for reducing the future burden of kidney cancer.

### 4.2. Comparisons with previous studies

In GBD 2021, several methodological refinements were introduced compared to GBD 2019.^[18]^ Since MIRs can exceed 1.00, the MIR input data were capped at the 95th percentile of the cleaned dataset, stratified by age group. This approach permitted MIRs above 1.00 in certain age groups while applying an upper constraint to prevent extreme values. The incorporation of additional datasets in GBD 2021 led to modifications in these caps compared to GBD 2019. For instance, the upper cap for age groups under 20 years was increased to 1.00. A comparison between Global Cancer Observatory (GLOBOCAN) 2022 and GBD 2021 revealed some discrepancies.^[1]^ For kidney cancer, the projected number of deaths in 2022 from this study was 164,488.44 (95% UI: 152,341.66–174,111.09), whereas GLOBOCAN reported 155,702 deaths. Furthermore, GLOBOCAN 2022 reported 277,574 incident cases in males and 156,845 in females, both of which were over 20,000 higher than the corresponding estimates in this study (males: 252,589.19 [95% UI: 237,746.50–268,143.49]; females: 135,239.53 [124,078.89–144,234.80]). However, in contrast to GLOBOCAN, this study includes both DALYs and projections through 2050, and utilizes time-series data from 1990, thus providing a more comprehensive analysis.^[1,17]^

### 4.3. Plausible mechanisms

There are notable differences between males and females in kidney cancer. High BMI was the leading risk factor for both sexes, while tobacco use was identified as a main risk factor, particularly among males. Prior research has shown that tobacco ranked as the leading risk factor for males and the seventh for females in terms of attributable DALYs, as males tend to smoke more frequently than females.^[19]^ In addition, high BMI was found to impose a more rapidly increasing health burden among females.^[20]^

Lower SDI regions exhibited higher MIR and reported increasing trends in kidney cancer burdens. Additionally, the highest DALY rates by age occurred 15 years earlier in the low SDI region than in the high SDI region. Previous research explains that each region’s healthcare and policy environments lead to these disparities.^[7,21,22]^ In low SDI regions, the cancer burden is increasing due to etiology transitions and aging populations.^[7]^ Furthermore, insufficient cancer management policies and low levels of primary health care have led to delays in achieving sustainable cancer control.^[7,21]^ Consequently, inadequate cancer management with early diagnosis delay in low SDI regions has resulted in the highest DALYs being recorded at a younger age. However, more concerning is the observation that low SDI region exhibited higher DALY rates in the 2 to 4 years group than high SDI region. This finding may be attributed to the fact that Wilms tumor, the most common malignant tumor in children, predominantly affects those living in low- and middle-income countries.^[22,23]^

Additionally, in only South Asia and Sub-Saharan Africa, this study observed an increase in the predicted ASDR under the improved scenario compared to 2021. This trend may be attributable to the high BMI burden currently observed in these regions.^[13,24]^ Given that high BMI is a leading risk factor for kidney cancer, its increasing prevalence may have contributed to the rising burden of kidney cancer. South Asia exhibited the most significant increase in the proportion of global type 2 diabetes DALYs associated with high BMI, while Sub-Saharan Africa also experienced a notable rise.^[24]^ Moreover, South Asia and Central Sub-Saharan Africa were among the regions with the greatest increases in high BMI (annualized rates of change increase of > 1.2%).^[13]^

### 4.4. Strengths and limitations

Several limitations should be acknowledged. First, although various subtypes of kidney cancer have distinct prognoses and epidemiological characteristics, this study did not differentiate among them (clear cell renal cell carcinoma, 80%; papillary renal cell carcinoma, 13–20%; chromophobe renal cell carcinoma, 5%).^[4]^ Second, the possibility of underdiagnosis in acquiring disease data exists. Since the early symptoms of kidney cancer are often ambiguous, misdiagnosis with other diseases may have led to an underestimation of its incidence.^[25]^ Third, there are disparities in both the quality and quantity of data across several regions. In developing countries and low-income regions, limited research on cancer may have contributed to inaccurate diagnoses, affecting the reliability of regional data.^[6,26]^ In these settings, estimates were generated through model-based extrapolation. Furthermore, to improve validity, verbal autopsy methods were employed, with multiple models tested and the most valid approach selected. Therefore, although the GBD framework incorporates multiple bias-adjustment strategies, residual disparities in regional quality may persist. Fourth, the intricate interactions among risk factors may have introduced uncertainty in data measurement and since this study only includes certain risk factors, it is not feasible to assess the contribution of additional ones.^[3]^ As the risk factor analysis does not encompass all potential factors of kidney cancer, the attributable burden should be interpreted with caution, as they may underestimate the overall contribution of the full spectrum of risk factors. As such, these estimates should be interpreted as reflecting the burden attributable to the 3 risk factors currently recognized within the GBD 2021 framework, rather than the totality of possible etiologic contributors. Fifth, this study possibly overlooked the detailed differences across individual countries. However, this limitation could have been mitigated by collecting data from 204 countries and analyzing the future ASDRs of individual nations. Sixth, it is important to acknowledge that MIRs slightly exceeded 1.0 in some age groups in interpreting our findings. This arises from the GBD 2021 estimation framework, which applies upper cap thresholds over 1.0 to MIR inputs, allowing MIR to exceed 1.0 in certain contexts but constraining it to a plausible maximum level. Seventh, the improved behavioral and metabolic risks scenario in this study should be interpreted as an aspirational counterfactual rather than a realistic projection. Within the GBD foresight framework, this scenario assumes the complete elimination of smoking, dietary risks, and high BMI by 2050, thereby representing the maximum potential reduction in kidney cancer burden attributable to modifiable risk factors. While this assumption is highly optimistic and unlikely to be achieved in practice, it provides an upper-bound estimate of the health gains that could be theoretically be realized. Finally, since the forecast only considered improved behavioral and metabolic risks, predictions under other scenarios should be considered in future studies. However, when investigating ASDRs in other scenarios, no significant changes were observed compared to the reference by 2050, thus, these 2 scenarios were excluded.

Hence, the findings of this study should be interpreted with caution, and future research must focus on enhancing data management and ensuring the collection of accurate and comprehensive data. Despite these limitations, this research provides a novel perspective by integrating GBD 2021 on kidney cancer with predictive modeling, offering a deeper insight into its projected burden. Furthermore, by effectively addressing behavioral and metabolic risks, this study provides valuable guidance for public health strategies aimed at mitigating kidney cancer mortality and incidence trends.

### 4.5. Clinical and policy implications

Although the GBD does not differentiate among subtypes of kidney cancer, research efforts should primarily focus on clear cell renal cell carcinoma, the most prevalent subtype and the 1 most commonly associated with poor prognosis.^[4]^ Particular attention should be directed toward regions where subtype-specific data remain scarce (e.g., Sub-Saharan Africa). In addition, papillary renal cell carcinoma is more common in males and non-Latino Black adults, while chromophobe renal cell carcinoma is more common in females, thereby necessitating tailored approaches.^[27]^ Furthermore, MIRs approaching or exceeding 1.0 in older populations emphasize that timely diagnosis and effective management of kidney cancer are crucial, as incidence in these groups more frequently progresses to mortality.

Analyzing the most significant risk factors attributable to the kidney cancer burden underscores the global challenge of managing high BMI. Although high BMI is known to be associated with a higher health burden in affluent regions, this study observed that high-income Asia Pacific exhibits a remarkably lower ASDR for high BMI compared to other high-income regions. These findings may provide valuable insights to guide regional public health strategies. In Singapore, through various healthcare policies such as the National Steps Challenge and the “Let’s BEAT Diabetes” campaign, there has been progress in overall physical activity levels and disease management.^[28]^ Therefore, implementing these policies and social movements in other high-income regions may help to decrease the high BMI burden.

However, other research indicates that the increased rate of high BMI is significant in low-income regions, and in South Asia and Sub-Saharan Africa, the health burden is expected to grow even with improved conditions by 2050.^[13,24]^ Additionally, in recent years, these 2 regions have reported a substantial number of deaths from non-communicable diseases.^[29,30]^ The inadequate primary healthcare infrastructure in these regions contributes to delays in early diagnosis, thereby exacerbating the burden of kidney cancer.^[21]^ Thus, establishing a well-structured healthcare governance system and efficient early diagnosis framework at the national level is essential. This could involve building healthcare infrastructure, training primary health workers, and collaborating with international organizations.^[31]^

The results of the improved behavioral and metabolic risks scenario underscore the potential reductions in future kidney cancer burden that may be achieved if modifiable risk factors such as smoking and high BMI were fully addressed. Although such complete elimination is unlikely, the scenario quantifies the upper bound of health gains that risk reduction strategies could deliver. Actual future projections will likely fall between the reference forecast and the improved behavioral and metabolic risks scenario, reinforcing the urgency of implementing effective policies and clinical interventions targeting these modifiable risks.

## 5. Conclusion

Globally, the total number of deaths and DALYs of kidney cancer has increased from 1990 to 2021, the rates of which are projected to continue slightly increasing by 2050. Kidney cancer is significantly influenced by high BMI and tobacco use, with high BMI having a greater impact on females, while tobacco use is more significant in males. The burden of kidney cancer is disproportionately higher in males and older populations. Although the total burden was higher in high SDI regions in 2021, ASRs increased more rapidly in low SDI regions from 1990 to 2021. This study underscores that addressing these disparities could be a strategic focus for kidney cancer prevention. Therefore, by fostering international collaboration, the global community needs to focus on region-specific prevention, early detection programs, and equitable access to tailored treatment.

## Author contributions

**Conceptualization**: Yoon Lee, Seohyun Hong, Jaehyun Kong, Hyeon Seok Hwang, Dong Keon Yon.

**Data curation**: Yoon Lee, Seohyun Hong, Jaehyun Kong, Hyeon Seok Hwang, Dong Keon Yon.

**Formal analysis**: Yoon Lee, Seohyun Hong, Jaehyun Kong, Hyeon Seok Hwang, Dong Keon Yon.

**Funding acquisition**: Dong Keon Yon.

**Investigation**: Yoon Lee, Seohyun Hong, Jaehyun Kong, Hyeon Seok Hwang, Dong Keon Yon.

**Methodology**: Yoon Lee, Seohyun Hong, Jaehyun Kong, Hyeon Seok Hwang, Dong Keon Yon.

**Project administration**: Yoon Lee, Seohyun Hong, Jaehyun Kong, Hyeon Seok Hwang, Dong Keon Yon.

**Resources**: Yoon Lee, Seohyun Hong, Jaehyun Kong, Hyeon Seok Hwang, Dong Keon Yon.

**Software**: Yoon Lee, Seohyun Hong, Jaehyun Kong, Hyeon Seok Hwang, Dong Keon Yon.

**Supervision**: Dong Keon Yon.

**Validation**: Yoon Lee, Seohyun Hong, Jaehyun Kong, Hyeon Seok Hwang, Dong Keon Yon.

**Visualization**: Yoon Lee, Seohyun Hong, Jaehyun Kong, Hyeon Seok Hwang, Dong Keon Yon.

**Writing – original draft**: Yoon Lee, Seohyun Hong, Jaehyun Kong, Hyeon Seok Hwang, Dong Keon Yon.

**Writing – review & editing**: Yoon Lee, Seohyun Hong, Jaehyun Kong, Sooji Lee, Hayeon Lee, Jinseok Lee, Jiseung Kang, Damiano Pizzol, Hyeon Seok Hwang, Dong Keon Yon.

## Supplementary Material
