# Supplementary material for: Global, regional, and national burden of kidney cancer, 1990 to 2021, and projections to 2050: A Global Burden of Disease Study 2021
Source: Medicine (Baltimore). 2026 May 12;104(49):e46059. doi: 10.1097/MD.0000000000046059 (PMC12688882; doi:10.1097/MD.0000000000046059)

Table S1. Classification of seven super-regions and 21 regions by Global Burden of Disease Study 2021.

GBD super regions	GBD regions	Country
Central Europe, Eastern Europe, and Central Asia	Central Asia	Armenia, Azerbaijan, Georgia, Kazakhstan, Kyrgyzstan, Mongolia, Tajikistan, Turkmenistan, Uzbekistan
	Central Europe	Albania, Bosnia and Herzegovina, Bulgaria, Croatia, Czechia, Hungary, Montenegro, North Macedonia, Poland, Romania, Serbia, Slovakia, Slovenia
	Eastern Europe	Belarus, Estonia, Latvia, Lithuania, Republic of Moldova, Russian Federation, Ukraine
High-income	Australasia	Australia, New Zealand
	High-income Asia Pacific	Brunei Darussalam, Japan, Republic of Korea, Singapore
	High-income North America	Canada, Greenland, United States of America
	Southern Latin America	Argentina, Chile, Uruguay
	Western Europe	Andorra, Austria, Belgium, Cyprus, Denmark, Finland, France, Germany, Greece, Iceland, Ireland, Israel, Italy, Luxembourg, Malta, Monaco, Netherlands, Norway, Portugal, San Marino, Spain, Sweden, Switzerland, United Kingdom
Latin America and Caribbean	Andean Latin America	Bolivia, Ecuador, Peru
	Caribbean	Antigua and Barbuda, Bahamas, Barbados, Belize, Bermuda, Cuba, Dominica, Dominican Republic, Grenada, Guyana, Haiti, Jamaica, Puerto Rico, Saint Kitts and Nevis, Saint Lucia, Saint Vincent and the Grenadines, Suriname, Trinidad and Tobago, United States Virgin Islands

North Africa and Middle East	North Africa and Middle East	Afghanistan, Algeria, Bahrain, Egypt, Iran (Islamic Republic of), Iraq, Jordan, Kuwait, Lebanon, Libya, Morocco, Oman, Palestine, Qatar, Saudi Arabia, Sudan, Syrian Arab Republic, Tunisia, Türkiye, United Arab Emirates, Yemen
South Asia	South Asia	Bangladesh, Bhutan, India, Nepal, Pakistan
Southeast Asia, East Asia, and Oceania	East Asia	China, Democratic People's Republic of Korea, Taiwan (Province of China)
	Oceania	American Samoa, Cook Islands, Fiji, Guam, Kiribati, Marshall Islands, Micronesia, Nauru, Niue, Northern Mariana Islands, Palau, Papua New Guinea, Samoa, Solomon Islands, Tokelau, Tonga, Tuvalu, Vanuatu
	Southeast Asia	Cambodia, Indonesia, Lao People's Democratic Republic, Malaysia, Maldives, Mauritius, Myanmar, Philippines, Seychelles, Sri Lanka, Thailand, Timor-Leste, Viet Nam
Sub-Saharan Africa	Central Sub-Saharan Africa	Angola, Central African Republic, Congo, Democratic Republic of Congo, Equatorial Guinea, Gabon
	Eastern Sub-Saharan Africa	Burundi, Comoros, Djibouti, Eritrea, Ethiopia, Kenya, Madagascar, Malawi, Mozambique, Rwanda, Somalia, South Sudan, Uganda, United Republic of Tanzania, Zambia
	Southern Sub-Saharan Africa	Botswana, Eswatini, Lesotho, Namibia, South Africa, Zimbabwe
	Western Sub-Saharan Africa	Benin, Burkina Faso, Cabo Verde, Cameroon, Chad, Côte d'Ivoire, Gambia, Ghana, Guinea, Guinea-Bissau, Liberia, Mali, Mauritania, Niger, Nigeria, Sao Tome and Principe, Senegal, Sierra Leone, Togo

Abbreviation: GBD, Global Burden of Disease Study.

Table S2. Annual percentage change in age-standardized incidence rates for kidney cancer from 1990 to 2000, 2000 to 2010, and 2010 to 2021 for both sexes, globally and by regions.

	1990–2000		2000–2010		2010–2021	
	APC (95% UI)	p-value	APC (95% UI)	p-value	APC (95% UI)	p-value
**Global**	**1.17 (0.90 to 1.44)**	<0.001	**0.94 (0.83 to 1.05)**	<0.001	**-0.58 (-0.70 to -0.47)**	<0.001
**SDI**						
High SDI	**1.70 (1.43 to 1.97)**	<0.001	**0.85 (0.77 to 0.92)**	<0.001	**-1.10 (-1.31 to -0.88)**	<0.001
High-middle SDI	**1.11 (0.56 to 1.66)**	0.001	**1.66 (1.38 to 1.95)**	<0.001	-0.10 (-0.23 to 0.03)	0.131
Middle SDI	**1.39 (1.22 to 1.56)**	<0.001	**1.99 (1.87 to 2.11)**	<0.001	**1.45 (1.35 to 1.56)**	<0.001
Low-middle SDI	**1.53 (1.38 to 1.67)**	<0.001	**1.76 (1.66 to 1.85)**	<0.001	**1.76 (1.59 to 1.93)**	<0.001
Low SDI	**0.23 (0.14 to 0.31)**	<0.001	**0.75 (0.63 to 0.88)**	<0.001	**1.58 (1.44 to 1.73)**	<0.001
**Central Europe, Eastern Europe, and Central Asia**	**1.67 (0.65 to 2.70)**	0.005	**1.94 (1.59 to 2.29)**	<0.001	**-0.25 (-0.41 to -0.08)**	0.007
Central Asia	**1.25 (0.03 to 2.48)**	0.045	**1.95 (1.67 to 2.24)**	<0.001	-0.24 (-0.66 to 0.18)	0.235
Central Europe	**3.10 (2.82 to 3.39)**	<0.001	**1.51 (1.11 to 1.92)**	<0.001	**-0.20 (-0.39 to -0.00)**	0.046
Eastern Europe	1.05 (-0.41 to 2.54)	0.139	**2.27 (1.86 to 2.69)**	<0.001	-0.14 (-0.40 to 0.11)	0.239
**High-income**	**1.73 (1.45 to 2.01)**	<0.001	**0.97 (0.88 to 1.05)**	<0.001	**-0.90 (-1.17 to -0.63)**	<0.001
Australasia	**2.21 (1.98 to 2.45)**	<0.001	**1.45 (1.23 to 1.68)**	<0.001	**-1.01 (-1.38 to -0.63)**	<0.001
High-income Asia Pacific	**3.38 (2.86 to 3.90)**	<0.001	**1.67 (1.27 to 2.07)**	<0.001	**-1.05 (-1.57 to -0.52)**	0.001
High-income North America	**1.42 (1.13 to 1.70)**	<0.001	0.08 (-0.10 to 0.27)	0.328	**-1.58 (-1.78 to -1.39)**	<0.001
Southern Latin America	**2.52 (2.30 to 2.74)**	<0.001	**1.05 (0.70 to 1.40)**	<0.001	**0.59 (0.12 to 1.07)**	0.019
Western Europe	**1.80 (1.43 to 2.16)**	<0.001	**1.53 (1.36 to 1.70)**	<0.001	**-0.68 (-1.07 to -0.28)**	0.004
**Latin America and Caribbean**	**1.78 (1.65 to 1.90)**	<0.001	**1.61 (1.48 to 1.75)**	<0.001	**1.56 (1.41 to 1.71)**	<0.001
Andean Latin America	**0.94 (0.13 to 1.75)**	0.028	**1.73 (1.04 to 2.41)**	<0.001	**1.52 (1.10 to 1.95)**	<0.001
Caribbean	**0.94 (0.66 to 1.22)**	<0.001	**1.42 (0.99 to 1.86)**	<0.001	**0.92 (0.57 to 1.28)**	<0.001
Central Latin America	**1.61 (1.33 to 1.88)**	<0.001	**1.66 (1.39 to 1.93)**	<0.001	**1.91 (1.71 to 2.12)**	<0.001
Tropical Latin America	**2.40 (1.99 to 2.82)**	<0.001	**1.50 (1.30 to 1.69)**	<0.001	**1.19 (0.93 to 1.45)**	<0.001

**North Africa and Middle East**	**1.63 (1.56 to 1.69)**	<0.001	**2.36 (2.07 to 2.65)**	<0.001	**1.21 (0.95 to 1.47)**	<0.001
**South Asia**	**2.02 (1.79 to 2.25)**	<0.001	**1.09 (0.96 to 1.23)**	<0.001	**2.37 (2.23 to 2.51)**	<0.001
**Southeast Asia, East Asia, and Oceania**	**1.45 (1.00 to 1.89)**	<0.001	**2.77 (2.60 to 2.95)**	<0.001	**1.28 (1.07 to 1.49)**	<0.001
East Asia	**1.24 (0.65 to 1.84)**	0.001	**3.26 (2.95 to 3.56)**	<0.001	**1.23 (0.98 to 1.49)**	<0.001
Oceania	**0.31 (0.24 to 0.37)**	<0.001	-0.24 (-0.56 to 0.08)	0.118	**0.83 (0.57 to 1.08)**	<0.001
Southeast Asia	**2.37 (2.11 to 2.63)**	<0.001	0.01 (-0.50 to 0.52)	0.980	**1.67 (1.58 to 1.77)**	<0.001
**Sub-Saharan Africa**	**0.49 (0.39 to 0.58)**	<0.001	**1.47 (1.39 to 1.55)**	<0.001	**1.30 (1.13 to 1.46)**	<0.001
Central Sub-Saharan Africa	**-0.60 (-0.71 to -0.49)**	<0.001	**0.64 (0.25 to 1.03)**	0.005	**1.95 (1.81 to 2.09)**	<0.001
Eastern Sub-Saharan Africa	**0.06 (0.03 to 0.08)**	<0.001	**0.95 (0.80 to 1.10)**	<0.001	**1.61 (1.56 to 1.66)**	<0.001
Southern Sub-Saharan Africa	**1.57 (1.02 to 2.12)**	<0.001	**2.00 (1.65 to 2.36)**	<0.001	**0.91 (0.68 to 1.14)**	<0.001
Western Sub-Saharan Africa	**0.79 (0.69 to 0.89)**	<0.001	**2.19 (2.11 to 2.28)**	<0.001	**1.10 (0.78 to 1.42)**	<0.001

Abbreviations: APC, annual percentage change; SDI, Socio-demographic Index; UI, uncertainty interval.

The numbers in bold indicate significant differences (p<0.05)

Table S3. Annual percentage change in age-standardized mortality rates for kidney cancer from 1990 to 2000, 2000 to 2010, and 2010 to 2021 for both sexes, globally and by regions.

	1990–2000		2000–2010		2010–2021	
	**APC (95% UI)**	**p-value**	**APC (95% UI)**	**p-value**	**APC (95% UI)**	**p-value**
**Global**	**0.23 (0.02 to 0.44)**	0.036	-0.08 (-0.20 to 0.04)	0.158	**-0.67 (-0.75 to -0.59)**	<0.001
**SDI**						
High SDI	**0.18 (0.01 to 0.34)**	0.037	**-0.31 (-0.40 to -0.22)**	<0.001	**-1.07 (-1.23 to -0.91)**	<0.001
High-middle SDI	**0.49 (0.03 to 0.95)**	0.038	0.12 (-0.14 to 0.38)	0.312	**-0.87 (-0.97 to -0.78)**	<0.001
Middle SDI	**0.68 (0.55 to 0.82)**	<0.001	**0.67 (0.53 to 0.80)**	<0.001	**0.53 (0.43 to 0.62)**	<0.001
Low-middle SDI	**1.24 (1.11 to 1.37)**	<0.001	**1.08 (1.01 to 1.15)**	<0.001	**1.16 (0.99 to 1.34)**	<0.001
Low SDI	0.05 (-0.06 to 0.16)	0.342	**0.24 (0.14 to 0.33)**	<0.001	**1.10 (0.93 to 1.27)**	<0.001
**Central Europe, Eastern Europe, and Central Asia**	**1.66 (0.87 to 2.45)**	0.001	**0.76 (0.49 to 1.04)**	<0.001	**-0.67 (-0.78 to -0.55)**	<0.001
Central Asia	**1.57 (0.45 to 2.69)**	0.011	**1.43 (1.13 to 1.73)**	<0.001	**-0.90 (-1.36 to -0.44)**	0.001
Central Europe	**1.69 (1.51 to 1.88)**	<0.001	**0.56 (0.23 to 0.89)**	0.004	**-0.42 (-0.54 to -0.30)**	<0.001
Eastern Europe	**1.63 (0.44 to 2.83)**	0.013	**0.83 (0.51 to 1.14)**	<0.001	**-0.78 (-0.99 to -0.58)**	<0.001
**High-income**	0.06 (-0.12 to 0.25)	0.472	**-0.20 (-0.30 to -0.10)**	0.001	**-0.91 (-1.14 to -0.68)**	<0.001
Australasia	-0.05 (-0.32 to 0.22)	0.698	**-0.42 (-0.67 to -0.18)**	0.004	**-1.10 (-1.40 to -0.80)**	<0.001
High-income Asia Pacific	**1.94 (1.54 to 2.35)**	<0.001	0.13 (-0.12 to 0.39)	0.269	**-1.00 (-1.42 to -0.57)**	<0.001
High-income North America	0.19 (-0.04 to 0.41)	0.090	**-0.57 (-0.72 to -0.42)**	<0.001	**-1.36 (-1.56 to -1.17)**	<0.001
Southern Latin America	**1.36 (1.01 to 1.70)**	<0.001	**0.49 (0.20 to 0.79)**	0.004	-0.13 (-0.58 to 0.33)	0.555
Western Europe	-0.26 (-0.54 to 0.02)	0.061	-0.07 (-0.19 to 0.06)	0.262	**-0.73 (-1.01 to -0.45)**	<0.001
**Latin America and Caribbean**	**0.66 (0.52 to 0.79)**	<0.001	**1.00 (0.89 to 1.12)**	<0.001	**1.02 (0.87 to 1.18)**	<0.001
Andean Latin America	-0.09 (-0.83 to 0.66)	0.802	**0.86 (0.23 to 1.49)**	0.013	**0.50 (0.10 to 0.90)**	0.020
Caribbean	-0.04 (-0.29 to 0.21)	0.718	**0.75 (0.31 to 1.19)**	0.004	**0.64 (0.31 to 0.97)**	0.001
Central Latin America	**0.31 (0.07 to 0.55)**	0.018	**1.00 (0.72 to 1.28)**	<0.001	**1.35 (1.13 to 1.56)**	<0.001
Tropical Latin America	**1.42 (1.00 to 1.85)**	<0.001	**0.99 (0.81 to 1.16)**	<0.001	**0.76 (0.48 to 1.05)**	<0.001

**North Africa and Middle East**	**0.69 (0.59 to 0.78)**	<0.001	**0.96 (0.65 to 1.27)**	<0.001	**0.54 (0.29 to 0.80)**	0.001
**South Asia**	**1.73 (1.40 to 2.05)**	<0.001	**0.39 (0.29 to 0.49)**	<0.001	**1.52 (1.40 to 1.65)**	<0.001
**Southeast Asia, East Asia, and Oceania**	**0.54 (0.11 to 0.98)**	0.020	**0.42 (0.14 to 0.70)**	0.008	0.02 (-0.24 to 0.29)	0.839
East Asia	0.14 (-0.43 to 0.71)	0.596	**0.47 (0.07 to 0.87)**	0.026	-0.21 (-0.52 to 0.11)	0.173
Oceania	**0.36 (0.28 to 0.45)**	<0.001	-0.08 (-0.30 to 0.14)	0.416	**0.67 (0.40 to 0.94)**	<0.001
Southeast Asia	**2.02 (1.74 to 2.30)**	<0.001	**-0.49 (-0.90 to -0.07)**	0.026	**0.83 (0.75 to 0.90)**	<0.001
**Sub-Saharan Africa**	**0.36 (0.28 to 0.44)**	<0.001	**1.05 (0.97 to 1.12)**	<0.001	**0.83 (0.67 to 0.99)**	<0.001
Central Sub-Saharan Africa	**-0.81 (-0.91 to -0.72)**	<0.001	0.24 (-0.09 to 0.56)	0.140	**1.45 (1.32 to 1.57)**	<0.001
Eastern Sub-Saharan Africa	**-0.10 (-0.12 to -0.08)**	<0.001	**0.52 (0.39 to 0.64)**	<0.001	**1.14 (1.08 to 1.20)**	<0.001
Southern Sub-Saharan Africa	**1.87 (1.36 to 2.39)**	<0.001	**1.59 (1.29 to 1.90)**	<0.001	0.18 (-0.06 to 0.41)	0.127
Western Sub-Saharan Africa	**0.61 (0.53 to 0.70)**	<0.001	**1.77 (1.69 to 1.85)**	<0.001	**0.68 (0.38 to 0.98)**	0.001

Abbreviations: APC, annual percentage change; SDI, Socio-demographic Index; UI, uncertainty interval.

The numbers in bold indicate significant differences (p<0.05).

Table S4. Annual percentage change in age-standardized disability-adjusted life year rates for kidney cancer from 1990 to 2000, 2000 to 2010, and 2010 to 2021 for both sexes, globally and by regions.

	1990–2000		2000–2010		2010–2021	
	APC (95% UI)	p-value	APC (95% UI)	p-value	APC (95% UI)	p-value
**Global**	-0.10 (-0.32 to 0.12)	0.342	**-0.28 (-0.41 to -0.16)**	0.001	**-0.86 (-0.94 to -0.78)**	<0.001
**SDI**						
High SDI	-0.04 (-0.21 to 0.14)	0.652	**-0.54 (-0.64 to -0.45)**	<0.001	**-1.47 (-1.64 to -1.29)**	<0.001
High-middle SDI	0.04 (-0.46 to 0.55)	0.853	**-0.30 (-0.58 to -0.02)**	0.037	**-1.15 (-1.25 to -1.05)**	<0.001
Middle SDI	0.13 (-0.07 to 0.33)	0.181	**0.41 (0.27 to 0.54)**	<0.001	**0.27 (0.18 to 0.35)**	<0.001
Low-middle SDI	**0.90 (0.79 to 1.01)**	<0.001	**0.94 (0.88 to 1.01)**	<0.001	**0.96 (0.76 to 1.17)**	<0.001
Low SDI	**-0.38 (-0.47 to -0.29)**	<0.001	**0.19 (0.11 to 0.27)**	0.001	**0.85 (0.64 to 1.07)**	<0.001
**Central Europe, Eastern Europe, and Central Asia**	**1.45 (0.58 to 2.32)**	0.004	0.33 (-0.02 to 0.68)	0.062	**-1.11 (-1.22 to -1.00)**	<0.001
Central Asia	**1.46 (0.34 to 2.59)**	0.016	**1.10 (0.77 to 1.43)**	<0.001	**-1.05 (-1.50 to -0.60)**	<0.001
Central Europe	**1.61 (1.40 to 1.83)**	<0.001	0.29 (-0.05 to 0.63)	0.088	**-0.86 (-1.01 to -0.72)**	<0.001
Eastern Europe	**1.39 (0.11 to 2.70)**	0.037	0.30 (-0.10 to 0.69)	0.126	**-1.23 (-1.43 to -1.03)**	<0.001
**High-income**	-0.17 (-0.36 to 0.03)	0.091	**-0.41 (-0.51 to -0.32)**	0.000	**-1.25 (-1.50 to -1.01)**	<0.001
Australasia	-0.25 (-0.57 to 0.08)	0.122	**-0.45 (-0.70 to -0.19)**	0.003	**-1.47 (-1.76 to -1.17)**	<0.001
High-income Asia Pacific	**1.33 (0.98 to 1.68)**	<0.001	-0.10 (-0.42 to 0.23)	0.508	**-1.61 (-2.03 to -1.19)**	<0.001
High-income North America	-0.09 (-0.33 to 0.15)	0.412	**-0.86 (-1.02 to -0.71)**	<0.001	**-1.65 (-1.85 to -1.44)**	<0.001
Southern Latin America	**0.68 (0.48 to 0.89)**	<0.001	**0.41 (0.14 to 0.67)**	0.008	-0.16 (-0.59 to 0.27)	0.417
Western Europe	**-0.46 (-0.72 to -0.19)**	0.004	**-0.34 (-0.47 to -0.21)**	<0.001	**-1.16 (-1.47 to -0.85)**	<0.001
**Latin America and Caribbean**	**0.38 (0.25 to 0.50)**	<0.001	**0.76 (0.65 to 0.87)**	<0.001	**0.74 (0.60 to 0.89)**	<0.001
Andean Latin America	-0.61 (-1.36 to 0.16)	0.107	0.50 (-0.15 to 1.16)	0.115	0.32 (-0.09 to 0.73)	0.116
Caribbean	-0.19 (-0.47 to 0.09)	0.164	**0.51 (0.11 to 0.91)**	0.017	**0.53 (0.25 to 0.82)**	0.002
Central Latin America	0.12 (-0.10 to 0.33)	0.257	**0.83 (0.56 to 1.10)**	<0.001	**1.25 (1.05 to 1.45)**	<0.001
Tropical Latin America	**1.03 (0.58 to 1.48)**	0.001	**0.74 (0.55 to 0.92)**	<0.001	0.19 (-0.07 to 0.45)	0.134

**North Africa and Middle East**	**0.32 (0.25 to 0.39)**	<0.001	**0.71 (0.49 to 0.94)**	<0.001	0.10 (-0.12 to 0.33)	0.336
**South Asia**	**1.38 (1.20 to 1.55)**	<0.001	**0.21 (0.08 to 0.34)**	0.004	**1.35 (1.19 to 1.50)**	<0.001
**Southeast Asia, East Asia, and Oceania**	-0.23 (-0.70 to 0.24)	0.302	0.19 (-0.10 to 0.47)	0.170	-0.21 (-0.44 to 0.01)	0.060
East Asia	**-0.66 (-1.25 to -0.07)**	0.033	0.16 (-0.24 to 0.57)	0.385	**-0.49 (-0.79 to -0.19)**	0.004
Oceania	**0.52 (0.47 to 0.57)**	<0.001	0.00 (-0.18 to 0.19)	0.955	**0.52 (0.32 to 0.71)**	<0.001
Southeast Asia	**1.85 (1.62 to 2.08)**	<0.001	**-0.55 (-0.90 to -0.19)**	0.008	**0.67 (0.58 to 0.76)**	<0.001
**Sub-Saharan Africa**	**-0.15 (-0.22 to -0.08)**	0.001	**0.87 (0.79 to 0.94)**	<0.001	**0.60 (0.34 to 0.85)**	<0.001
Central Sub-Saharan Africa	**-0.83 (-0.93 to -0.73)**	<0.001	0.23 (-0.10 to 0.56)	0.148	**1.17 (0.98 to 1.36)**	<0.001
Eastern Sub-Saharan Africa	**-0.69 (-0.76 to -0.61)**	<0.001	**0.36 (0.22 to 0.50)**	<0.001	**0.80 (0.68 to 0.92)**	<0.001
Southern Sub-Saharan Africa	**1.15 (0.59 to 1.71)**	0.001	**1.56 (1.15 to 1.98)**	<0.001	**0.23 (0.07 to 0.40)**	0.010
Western Sub-Saharan Africa	**0.23 (0.15 to 0.31)**	<0.001	**1.40 (1.30 to 1.51)**	<0.001	**0.48 (0.01 to 0.95)**	0.048

Abbreviations: APC, annual percentage change; DALYs, disability-adjusted life years; SDI, Socio-demographic Index; UI, uncertainty interval.

The numbers in bold indicate significant differences (p<0.05).

Table S5. Age-standardized rates (per 100,000 population) of incidence, mortality, and disability-adjusted life years and mortality-to-incidence ratio for kidney cancer in 2021 by age group, globally.

Age	ASIR, per 100,000 population (95% UI)		ASMR, per 100,000 population (95% UI)		ASDR, per 100,000 population (95% UI)		MIR (95% UI)	
	Males	Females	Males	Females	Males	Females	Males	Females
<1 year	1.10 (0.80 to 1.42)	1.06 (0.82 to 1.41)	0.44 (0.30 to 0.57)	0.29 (0.21 to 0.40)	39.63 (27.72 to 51.44)	26.76 (19.69 to 36.81)	0.40 (0.26 to 0.60)	0.28 (0.18 to 0.43)
2-4 years	0.76 (0.52 to 1.00)	0.75 (0.54 to 0.99)	0.33 (0.22 to 0.45)	0.25 (0.17 to 0.34)	29.19 (19.55 to 39.77)	21.95 (15.15 to 30.29)	0.44 (0.27 to 0.71)	0.33 (0.20 to 0.53)
5-9 years	0.30 (0.25 to 0.35)	0.38 (0.33 to 0.44)	0.09 (0.07 to 0.11)	0.08 (0.07 to 0.09)	7.59 (6.24 to 8.95)	6.71 (5.75 to 7.95)	0.30 (0.23 to 0.38)	0.21 (0.17 to 0.26)
10-14 years	0.15 (0.13 to 0.17)	0.18 (0.16 to 0.21)	0.04 (0.03 to 0.05)	0.03 (0.03 to 0.04)	3.27 (2.74 to 3.79)	2.71 (2.39 to 3.13)	0.27 (0.22 to 0.34)	0.19 (0.16 to 0.23)
15-19 years	0.13 (0.11 to 0.14)	0.15 (0.14 to 0.17)	0.03 (0.03 to 0.03)	0.03 (0.02 to 0.03)	2.21 (1.97 to 2.50)	2.13 (1.89 to 2.46)	0.24 (0.20 to 0.28)	0.18 (0.15 to 0.22)
20-24 years	0.24 (0.22 to 0.26)	0.23 (0.21 to 0.26)	0.05 (0.05 to 0.06)	0.04 (0.04 to 0.05)	3.73 (3.46 to 4.06)	3.11 (2.79 to 3.45)	0.23 (0.20 to 0.25)	0.19 (0.16 to 0.22)
25-29 years	0.45 (0.42 to 0.49)	0.35 (0.32 to 0.40)	0.09 (0.09 to 0.10)	0.06 (0.05 to 0.07)	6.08 (5.61 to 6.62)	3.92 (3.50 to 4.39)	0.21 (0.19 to 0.23)	0.17 (0.14 to 0.20)
30-34 years	0.91 (0.83 to 1.02)	0.61 (0.55 to 0.68)	0.19 (0.17 to 0.21)	0.10 (0.09 to 0.11)	11.26 (10.24 to 12.57)	5.83 (5.23 to 6.49)	0.21 (0.18 to 0.24)	0.16 (0.13 to 0.18)
35-39 years	1.90 (1.74 to 2.09)	1.08 (0.99 to 1.20)	0.37 (0.34 to 0.41)	0.16 (0.14 to 0.17)	20.41 (18.76 to 22.46)	8.84 (8.06 to 9.73)	0.19 (0.17 to 0.22)	0.15 (0.13 to 0.17)
40-44 years	3.79 (3.52 to 4.11)	1.85 (1.71 to 2.01)	0.77 (0.71 to 0.83)	0.28 (0.26 to 0.30)	38.62 (35.55 to 42.10)	14.42 (13.33 to 15.61)	0.20 (0.18 to 0.23)	0.15 (0.14 to 0.17)

**45-49 years**	6.13 (5.64 to 6.70)	2.66 (2.47 to 2.86)	1.60 (1.47 to 1.75)	0.54 (0.50 to 0.58)	71.78 (65.84 to 78.39)	24.40 (22.74 to 26.21)	0.26 (0.23 to 0.30)	0.20 (0.18 to 0.22)
**50-54 years**	10.10 (9.33 to 10.98)	4.07 (3.79 to 4.35)	3.05 (2.81 to 3.31)	0.98 (0.92 to 1.05)	120.97 (111.85 to 131.79)	39.35 (36.63 to 41.99)	0.30 (0.27 to 0.34)	0.24 (0.22 to 0.27)
**55-59 years**	15.05 (14.09 to 16.18)	6.05 (5.66 to 6.44)	5.21 (4.87 to 5.60)	1.74 (1.64 to 1.85)	181.24 (169.41 to 195.09)	61.08 (57.21 to 64.89)	0.35 (0.31 to 0.38)	0.29 (0.26 to 0.31)
**60-64 years**	21.65 (20.56 to 22.93)	9.70 (9.09 to 10.30)	8.06 (7.64 to 8.48)	3.10 (2.92 to 3.27)	241.86 (230.13 to 255.45)	93.72 (87.80 to 99.03)	0.37 (0.35 to 0.40)	0.32 (0.29 to 0.35)
**65-69 years**	27.52 (25.95 to 29.27)	12.95 (11.89 to 13.82)	11.15 (10.55 to 11.82)	4.68 (4.33 to 4.98)	282.28 (267.60 to 299.49)	119.12 (110.18 to 126.86)	0.41 (0.37 to 0.44)	0.36 (0.33 to 0.40)
**70-74 years**	36.54 (34.27 to 38.53)	17.51 (15.96 to 18.78)	15.92 (14.98 to 16.78)	6.96 (6.36 to 7.41)	332.13 (312.52 to 351.56)	145.83 (133.54 to 155.70)	0.44 (0.40 to 0.47)	0.40 (0.36 to 0.44)
**75-79 years**	42.70 (39.54 to 45.25)	20.13 (17.54 to 21.66)	22.04 (20.58 to 23.42)	9.72 (8.62 to 10.46)	366.38 (342.25 to 389.59)	161.91 (143.09 to 173.32)	0.52 (0.47 to 0.57)	0.48 (0.42 to 0.56)
**80-84 years**	46.63 (41.10 to 50.22)	22.89 (18.65 to 25.38)	31.21 (27.86 to 33.49)	14.39 (11.99 to 15.92)	403.08 (360.59 to 433.28)	186.02 (154.98 to 205.32)	0.67 (0.59 to 0.77)	0.63 (0.51 to 0.77)
**85-89 years**	56.19 (48.27 to 60.58)	25.95 (19.83 to 29.55)	47.59 (41.41 to 51.16)	20.83 (16.22 to 23.60)	483.79 (421.56 to 520.01)	211.47 (165.29 to 240.37)	0.85 (0.73 to 0.98)	0.80 (0.62 to 1.04)
**90-94 years**	51.77 (43.03 to 56.37)	25.51 (18.97 to 29.07)	62.47 (52.04 to 67.84)	29.84 (22.26 to 33.92)	548.80 (459.05 to 596.59)	262.02 (195.06 to 298.15)	1.21 (1.01 to 1.45)	1.17 (0.88 to 1.55)
**95+ years**	43.02 (33.75 to 48.90)	27.33 (19.40 to 32.01)	55.85 (44.04 to 63.45)	34.49 (24.57 to 40.33)	457.88 (361.47 to 518.93)	281.80 (200.64 to 329.48)	1.30 (1.01 to 1.67)	1.26 (0.91 to 1.76)

Abbreviations: DALYs, disability-adjusted life years; ASIR, age-standardized incidence rate; ASMR, age-standardized mortality rate; ASDR, age-standardized DALY rate; MIR, mortality-to-incidence ratio; UI, uncertainty interval.

Table S6. Age-standardized rates (per 100,000 population) of incidence, mortality, and disability-adjusted life years and mortality-to-incidence ratio for kidney cancer in 2021 by age group, in high Socio-demographic Index region.

Age	ASIR, per 100,000 population (95% UI)		ASMR, per 100,000 population (95% UI)		ASDR, per 100,000 population (95% UI)		MIR (95% UI)	
	Males	Females	Males	Females	Males	Females	Males	Females
<1 year	0.91 (0.78 to 1.04)	1.19 (1.02 to 1.38)	0.13 (0.11 to 0.15)	0.13 (0.11 to 0.15)	12.19 (10.52 to 14.05)	12.11 (10.42 to 14.06)	0.14 (0.12 to 0.18)	0.11 (0.09 to 0.13)
2-4 years	0.38 (0.33 to 0.43)	0.53 (0.46 to 0.60)	0.05 (0.05 to 0.06)	0.06 (0.05 to 0.06)	4.77 (4.14 to 5.48)	5.14 (4.54 to 5.84)	0.14 (0.12 to 0.17)	0.11 (0.09 to 0.13)
5-9 years	0.51 (0.47 to 0.55)	0.85 (0.79 to 0.91)	0.07 (0.06 to 0.08)	0.09 (0.08 to 0.09)	6.09 (5.57 to 6.59)	7.83 (7.35 to 8.38)	0.14 (0.12 to 0.16)	0.10 (0.10 to 0.12)
10-14 years	0.26 (0.24 to 0.28)	0.43 (0.40 to 0.46)	0.04 (0.03 to 0.04)	0.04 (0.04 to 0.05)	2.92 (2.67 to 3.19)	3.71 (3.45 to 4.01)	0.14 (0.12 to 0.16)	0.10 (0.09 to 0.12)
15-19 years	0.28 (0.27 to 0.30)	0.39 (0.36 to 0.43)	0.04 (0.04 to 0.04)	0.04 (0.04 to 0.04)	2.97 (2.79 to 3.15)	3.16 (2.94 to 3.43)	0.14 (0.13 to 0.15)	0.10 (0.09 to 0.12)
20-24 years	0.56 (0.53 to 0.59)	0.54 (0.51 to 0.58)	0.08 (0.07 to 0.08)	0.06 (0.06 to 0.06)	5.59 (5.27 to 5.95)	4.29 (4.06 to 4.57)	0.14 (0.13 to 0.15)	0.11 (0.10 to 0.12)
25-29 years	0.99 (0.93 to 1.05)	0.82 (0.77 to 0.89)	0.13 (0.12 to 0.14)	0.08 (0.08 to 0.09)	8.64 (8.09 to 9.27)	5.63 (5.27 to 6.03)	0.13 (0.12 to 0.14)	0.10 (0.09 to 0.11)
30-34 years	1.78 (1.63 to 1.93)	1.37 (1.26 to 1.48)	0.24 (0.22 to 0.26)	0.14 (0.13 to 0.15)	14.63 (13.40 to 16.12)	8.64 (8.00 to 9.25)	0.13 (0.12 to 0.15)	0.10 (0.09 to 0.11)
35-39 years	3.87 (3.56 to 4.20)	2.34 (2.16 to 2.53)	0.49 (0.45 to 0.53)	0.22 (0.21 to 0.24)	27.85 (25.72 to 30.11)	12.93 (12.01 to 13.98)	0.13 (0.11 to 0.14)	0.10 (0.09 to 0.11)
40-44 years	7.65 (7.22 to 8.12)	4.05 (3.78 to 4.32)	1.02 (0.96 to 1.08)	0.41 (0.39 to 0.43)	52.81 (49.46 to 56.51)	21.52 (20.22 to 22.81)	0.13 (0.12 to 0.14)	0.10 (0.09 to 0.11)

**45-49 years**	12.34 (11.66 to 13.00)	5.89 (5.53 to 6.28)	2.20 (2.08 to 2.30)	0.81 (0.77 to 0.85)	100.55 (95.00 to 105.89)	37.50 (35.61 to 39.72)	0.18 (0.17 to 0.19)	0.14 (0.13 to 0.15)
**50-54 years**	20.37 (19.27 to 21.51)	8.86 (8.35 to 9.39)	4.42 (4.20 to 4.65)	1.50 (1.43 to 1.56)	178.54 (169.02 to 188.51)	61.39 (58.47 to 64.48)	0.22 (0.20 to 0.23)	0.17 (0.16 to 0.18)
**55-59 years**	30.78 (29.34 to 32.38)	12.63 (11.91 to 13.35)	7.88 (7.52 to 8.28)	2.65 (2.53 to 2.77)	277.96 (264.75 to 293.56)	94.55 (89.99 to 99.77)	0.26 (0.24 to 0.27)	0.21 (0.19 to 0.23)
**60-64 years**	44.29 (41.81 to 46.99)	18.61 (17.36 to 19.95)	12.47 (11.78 to 13.17)	4.46 (4.20 to 4.71)	379.50 (358.88 to 401.47)	137.23 (128.75 to 145.47)	0.28 (0.26 to 0.31)	0.24 (0.22 to 0.26)
**65-69 years**	58.39 (55.52 to 61.24)	25.29 (23.29 to 26.95)	18.51 (17.65 to 19.40)	7.06 (6.57 to 7.44)	474.38 (451.99 to 499.99)	182.74 (169.78 to 192.56)	0.32 (0.30 to 0.34)	0.28 (0.25 to 0.31)
**70-74 years**	74.67 (69.78 to 79.16)	33.00 (30.01 to 35.52)	26.43 (24.84 to 27.97)	10.55 (9.62 to 11.16)	556.69 (524.03 to 590.90)	223.88 (203.04 to 237.63)	0.35 (0.32 to 0.39)	0.32 (0.29 to 0.36)
**75-79 years**	87.87 (81.54 to 92.81)	40.07 (34.58 to 43.67)	38.04 (35.56 to 40.22)	16.08 (14.10 to 17.29)	637.05 (594.07 to 674.72)	270.34 (237.53 to 291.72)	0.43 (0.40 to 0.47)	0.40 (0.35 to 0.47)
**80-84 years**	88.72 (77.20 to 97.24)	42.61 (33.68 to 48.43)	52.35 (45.88 to 57.33)	23.49 (18.84 to 26.47)	679.20 (595.09 to 743.46)	305.31 (246.40 to 344.83)	0.59 (0.50 to 0.69)	0.55 (0.43 to 0.70)
**85-89 years**	97.55 (83.23 to 105.53)	45.47 (34.18 to 52.94)	76.29 (65.17 to 82.36)	33.74 (25.30 to 38.75)	776.89 (663.97 to 839.35)	342.64 (258.24 to 393.30)	0.78 (0.67 to 0.92)	0.74 (0.55 to 0.99)
**90-94 years**	84.24 (69.89 to 92.60)	41.03 (29.95 to 47.61)	98.81 (82.09 to 108.23)	46.86 (34.40 to 54.04)	867.90 (722.93 to 950.59)	411.34 (301.05 to 474.10)	1.17 (0.97 to 1.42)	1.14 (0.84 to 1.56)
**95+ years**	68.17 (52.98 to 78.44)	41.37 (28.93 to 48.93)	86.31 (67.67 to 99.00)	51.42 (36.03 to 60.50)	705.49 (552.70 to 812.12)	418.76 (293.92 to 493.24)	1.27 (0.97 to 1.65)	1.24 (0.88 to 1.76)

Abbreviations: DALYs, disability-adjusted life years; ASIR, age-standardized incidence rate; ASMR, age-standardized mortality rate; ASDR, age-standardized DALY rate; MIR, mortality-to-incidence ratio; UI, uncertainty interval.

Table S7. Age-standardized rates (per 100,000 population) of incidence, mortality, and disability-adjusted life years and mortality-to-incidence ratio for kidney cancer in 2021 by age group, in high-middle Socio-demographic Index region.

	ASIR, per 100,000 population (95% UI)		ASMR, per 100,000 population (95% UI)		ASDR, per 100,000 population (95% UI)		MIR (95% UI)	
**Age**	**Males**	**Females**	**Males**	**Females**	**Males**	**Females**	**Males**	**Females**
**<1 year**	1.44 (1.10 to 1.86)	1.70 (1.20 to 2.41)	0.29 (0.22 to 0.38)	0.21 (0.15 to 0.28)	26.84 (20.26 to 34.73)	19.35 (14.01 to 26.49)	0.20 (0.14 to 0.30)	0.12 (0.07 to 0.20)
**2-4 years**	0.56 (0.43 to 0.74)	0.73 (0.52 to 1.03)	0.11 (0.09 to 0.15)	0.09 (0.07 to 0.13)	10.20 (7.80 to 13.55)	8.40 (5.92 to 11.67)	0.20 (0.14 to 0.31)	0.13 (0.08 to 0.21)
**5-9 years**	0.56 (0.46 to 0.68)	0.66 (0.54 to 0.80)	0.11 (0.09 to 0.13)	0.08 (0.07 to 0.09)	9.08 (7.50 to 10.89)	6.91 (5.77 to 8.13)	0.19 (0.15 to 0.25)	0.12 (0.09 to 0.16)
**10-14 years**	0.33 (0.28 to 0.40)	0.38 (0.31 to 0.48)	0.06 (0.05 to 0.07)	0.05 (0.04 to 0.06)	4.92 (4.20 to 5.89)	3.84 (3.15 to 4.66)	0.18 (0.14 to 0.23)	0.12 (0.09 to 0.17)
**15-19 years**	0.34 (0.29 to 0.40)	0.34 (0.28 to 0.41)	0.05 (0.05 to 0.06)	0.04 (0.03 to 0.05)	4.11 (3.51 to 4.77)	3.15 (2.63 to 3.73)	0.16 (0.13 to 0.20)	0.12 (0.09 to 0.16)
**20-24 years**	0.63 (0.54 to 0.73)	0.49 (0.41 to 0.59)	0.10 (0.09 to 0.12)	0.06 (0.06 to 0.08)	7.24 (6.28 to 8.36)	4.60 (3.95 to 5.36)	0.16 (0.13 to 0.20)	0.13 (0.10 to 0.17)
**25-29 years**	1.02 (0.88 to 1.22)	0.69 (0.57 to 0.83)	0.16 (0.14 to 0.19)	0.08 (0.07 to 0.10)	10.60 (9.29 to 12.29)	5.59 (4.68 to 6.63)	0.16 (0.13 to 0.20)	0.12 (0.09 to 0.16)
**30-34 years**	1.81 (1.54 to 2.16)	1.08 (0.91 to 1.29)	0.30 (0.26 to 0.36)	0.13 (0.11 to 0.16)	18.44 (15.94 to 21.93)	8.10 (6.83 to 9.70)	0.17 (0.13 to 0.21)	0.12 (0.09 to 0.16)
**35-39 years**	3.55 (3.06 to 4.11)	1.86 (1.56 to 2.17)	0.59 (0.51 to 0.68)	0.21 (0.18 to 0.24)	32.71 (28.37 to 37.81)	11.96 (10.19 to 13.91)	0.16 (0.13 to 0.20)	0.11 (0.09 to 0.14)
**40-44 years**	7.17 (6.37 to 8.32)	3.15 (2.78 to 3.57)	1.23 (1.10 to 1.41)	0.38 (0.34 to 0.42)	62.66 (55.68 to 72.08)	19.64 (17.49 to 21.87)	0.17 (0.14 to 0.21)	0.12 (0.10 to 0.14)

**45-49 years**	10.62 (9.36 to 12.27)	4.12 (3.59 to 4.69)	2.46 (2.16 to 2.78)	0.68 (0.61 to 0.76)	110.68 (97.80 to 125.77)	31.28 (27.79 to 35.26)	0.23 (0.19 to 0.28)	0.17 (0.14 to 0.20)
**50-54 years**	16.13 (14.19 to 18.33)	6.00 (5.33 to 6.77)	4.48 (3.96 to 5.11)	1.24 (1.11 to 1.38)	178.59 (158.43 to 203.65)	50.26 (44.72 to 56.15)	0.28 (0.23 to 0.33)	0.21 (0.18 to 0.24)
**55-59 years**	22.51 (20.15 to 25.46)	8.86 (7.97 to 9.88)	7.49 (6.72 to 8.43)	2.28 (2.05 to 2.52)	260.92 (233.46 to 292.66)	80.22 (71.83 to 88.68)	0.33 (0.28 to 0.39)	0.26 (0.22 to 0.30)
**60-64 years**	31.67 (29.08 to 34.51)	14.53 (13.20 to 15.91)	11.79 (10.87 to 12.86)	4.31 (3.92 to 4.68)	353.50 (325.76 to 386.10)	130.76 (118.80 to 142.39)	0.37 (0.33 to 0.42)	0.30 (0.26 to 0.34)
**65-69 years**	37.07 (33.72 to 40.82)	18.38 (16.57 to 20.40)	15.36 (13.98 to 16.78)	6.38 (5.77 to 7.03)	388.57 (353.80 to 426.04)	162.79 (146.88 to 179.05)	0.41 (0.36 to 0.47)	0.35 (0.30 to 0.40)
**70-74 years**	45.42 (41.50 to 49.40)	23.38 (20.89 to 25.86)	20.77 (19.09 to 22.54)	9.22 (8.32 to 10.06)	432.83 (397.32 to 471.41)	193.44 (174.09 to 210.86)	0.46 (0.41 to 0.52)	0.39 (0.34 to 0.46)
**75-79 years**	49.58 (44.41 to 54.36)	24.27 (21.30 to 27.00)	26.69 (24.17 to 29.12)	11.94 (10.60 to 13.20)	442.78 (402.43 to 483.10)	198.32 (175.76 to 219.13)	0.54 (0.47 to 0.62)	0.49 (0.42 to 0.58)
**80-84 years**	52.64 (46.97 to 57.37)	26.19 (21.92 to 29.31)	37.17 (33.38 to 40.31)	17.06 (14.40 to 18.92)	478.51 (429.35 to 519.82)	219.79 (186.27 to 244.29)	0.71 (0.62 to 0.81)	0.65 (0.54 to 0.79)
**85-89 years**	59.98 (51.53 to 66.63)	25.87 (19.89 to 29.94)	53.00 (46.19 to 58.53)	21.60 (16.98 to 24.69)	537.78 (468.76 to 594.91)	219.34 (172.82 to 251.69)	0.88 (0.74 to 1.05)	0.83 (0.64 to 1.09)
**90-94 years**	50.86 (42.58 to 56.16)	21.80 (16.57 to 24.90)	62.70 (52.77 to 68.79)	25.93 (20.09 to 29.45)	550.87 (464.37 to 605.06)	227.93 (176.91 to 258.80)	1.23 (1.02 to 1.49)	1.19 (0.91 to 1.56)
**95+ years**	41.21 (32.49 to 47.03)	19.88 (14.39 to 23.10)	55.40 (43.75 to 62.96)	25.54 (18.67 to 29.59)	457.71 (361.62 to 521.30)	211.44 (154.83 to 245.04)	1.34 (1.05 to 1.73)	1.28 (0.94 to 1.76)

Abbreviations: DALYs, disability-adjusted life years; ASIR, age-standardized incidence rate; ASMR, age-standardized mortality rate; ASDR, age-standardized DALY rate; MIR, mortality-to-incidence ratio; UI, uncertainty interval.

Table S8. Age-standardized rates (per 100,000 population) of incidence, mortality, and disability-adjusted life years and mortality-to-incidence ratio for kidney cancer in 2021 by age group, in middle Socio-demographic Index region.

Age	ASIR, per 100,000 population (95% UI)		ASMR, per 100,000 population (95% UI)		ASDR, per 100,000 population (95% UI)		MIR (95% UI)	
	Males	Females	Males	Females	Males	Females	Males	Females
<1 year	1.10 (0.79 to 1.45)	0.96 (0.69 to 1.40)	0.34 (0.24 to 0.45)	0.21 (0.16 to 0.30)	31.16 (22.16 to 40.79)	19.69 (14.57 to 27.32)	0.31 (0.20 to 0.48)	0.22 (0.14 to 0.38)
2-4 years	0.57 (0.41 to 0.77)	0.53 (0.39 to 0.72)	0.19 (0.14 to 0.26)	0.12 (0.09 to 0.17)	16.77 (12.03 to 22.76)	11.04 (8.15 to 14.84)	0.33 (0.21 to 0.53)	0.23 (0.15 to 0.36)
5-9 years	0.38 (0.30 to 0.46)	0.38 (0.31 to 0.48)	0.11 (0.09 to 0.13)	0.08 (0.07 to 0.09)	9.33 (7.55 to 11.29)	6.67 (5.53 to 7.94)	0.29 (0.22 to 0.39)	0.20 (0.15 to 0.28)
10-14 years	0.20 (0.16 to 0.24)	0.20 (0.16 to 0.24)	0.06 (0.05 to 0.07)	0.04 (0.03 to 0.05)	4.48 (3.72 to 5.30)	3.22 (2.68 to 3.78)	0.28 (0.22 to 0.37)	0.20 (0.16 to 0.27)
15-19 years	0.14 (0.12 to 0.16)	0.16 (0.14 to 0.20)	0.04 (0.03 to 0.05)	0.03 (0.03 to 0.04)	2.95 (2.60 to 3.44)	2.55 (2.16 to 2.96)	0.29 (0.23 to 0.36)	0.21 (0.16 to 0.27)
20-24 years	0.23 (0.20 to 0.26)	0.24 (0.21 to 0.28)	0.07 (0.06 to 0.07)	0.05 (0.05 to 0.06)	4.65 (4.21 to 5.18)	3.80 (3.29 to 4.35)	0.30 (0.25 to 0.35)	0.22 (0.18 to 0.27)
25-29 years	0.42 (0.37 to 0.47)	0.34 (0.29 to 0.40)	0.11 (0.10 to 0.12)	0.07 (0.06 to 0.08)	7.21 (6.55 to 8.04)	4.45 (3.82 to 5.08)	0.27 (0.23 to 0.31)	0.20 (0.16 to 0.25)
30-34 years	0.83 (0.72 to 0.97)	0.53 (0.45 to 0.62)	0.21 (0.18 to 0.24)	0.10 (0.09 to 0.12)	12.33 (10.91 to 14.15)	6.18 (5.34 to 7.08)	0.25 (0.21 to 0.31)	0.19 (0.16 to 0.24)
35-39 years	1.60 (1.37 to 1.86)	0.90 (0.78 to 1.04)	0.38 (0.34 to 0.43)	0.16 (0.14 to 0.18)	21.11 (18.63 to 23.95)	9.01 (7.97 to 10.15)	0.24 (0.20 to 0.29)	0.18 (0.15 to 0.22)
40-44 years	2.88 (2.52 to 3.30)	1.41 (1.25 to 1.57)	0.75 (0.67 to 0.84)	0.28 (0.25 to 0.31)	37.17 (33.17 to 41.91)	14.24 (12.75 to 15.76)	0.26 (0.22 to 0.31)	0.20 (0.17 to 0.24)

**45-49 years**	4.35 (3.77 to 5.05)	1.87 (1.66 to 2.11)	1.43 (1.27 to 1.64)	0.50 (0.45 to 0.56)	63.64 (56.79 to 72.84)	22.43 (20.06 to 24.87)	0.33 (0.27 to 0.40)	0.27 (0.23 to 0.32)
**50-54 years**	6.73 (5.89 to 7.87)	2.59 (2.30 to 2.91)	2.53 (2.25 to 2.88)	0.83 (0.74 to 0.92)	99.73 (88.54 to 114.24)	32.78 (29.49 to 36.39)	0.38 (0.31 to 0.46)	0.32 (0.27 to 0.38)
**55-59 years**	9.07 (7.87 to 10.46)	3.48 (3.09 to 3.95)	3.97 (3.54 to 4.49)	1.35 (1.21 to 1.51)	137.16 (122.34 to 154.92)	46.81 (42.03 to 52.44)	0.44 (0.36 to 0.53)	0.39 (0.33 to 0.46)
**60-64 years**	11.16 (9.99 to 12.56)	5.26 (4.66 to 5.86)	5.51 (5.04 to 6.11)	2.31 (2.08 to 2.56)	163.70 (149.47 to 181.27)	68.83 (61.86 to 76.06)	0.49 (0.43 to 0.58)	0.44 (0.38 to 0.51)
**65-69 years**	13.22 (11.67 to 15.04)	6.41 (5.55 to 7.31)	7.01 (6.32 to 7.80)	3.17 (2.81 to 3.55)	175.71 (158.08 to 195.63)	79.61 (70.42 to 89.14)	0.53 (0.45 to 0.63)	0.49 (0.41 to 0.60)
**70-74 years**	16.32 (14.49 to 18.42)	7.72 (6.65 to 8.89)	9.47 (8.50 to 10.51)	4.30 (3.76 to 4.86)	195.46 (175.19 to 217.26)	88.82 (77.63 to 100.97)	0.58 (0.50 to 0.68)	0.56 (0.46 to 0.68)
**75-79 years**	19.60 (16.98 to 22.53)	8.25 (7.12 to 9.41)	13.39 (11.83 to 15.10)	5.69 (4.96 to 6.43)	220.49 (195.36 to 248.62)	93.47 (81.77 to 105.66)	0.68 (0.57 to 0.83)	0.69 (0.57 to 0.83)
**80-84 years**	19.13 (16.56 to 21.73)	7.83 (6.53 to 8.93)	16.58 (14.53 to 18.72)	6.95 (5.87 to 7.93)	212.64 (186.47 to 239.75)	88.95 (75.14 to 101.41)	0.87 (0.72 to 1.05)	0.89 (0.72 to 1.10)
**85-89 years**	23.57 (20.14 to 27.24)	8.37 (6.51 to 9.85)	24.65 (21.15 to 28.21)	8.96 (7.00 to 10.51)	249.62 (214.82 to 286.52)	90.85 (71.10 to 106.91)	1.05 (0.85 to 1.29)	1.07 (0.81 to 1.42)
**90-94 years**	19.65 (16.49 to 22.35)	7.59 (5.96 to 8.80)	26.41 (22.35 to 30.00)	10.24 (8.08 to 11.84)	232.28 (196.09 to 264.18)	90.03 (70.75 to 104.36)	1.34 (1.09 to 1.66)	1.35 (1.03 to 1.76)
**95+ years**	12.47 (9.37 to 14.64)	6.60 (4.93 to 7.75)	18.43 (14.11 to 21.48)	9.72 (7.29 to 11.33)	152.43 (116.58 to 178.09)	80.15 (59.95 to 93.68)	1.48 (1.10 to 1.99)	1.47 (1.09 to 2.00)

Abbreviations: DALYs, disability-adjusted life years; ASIR, age-standardized incidence rate; ASMR, age-standardized mortality rate; ASDR, age-standardized DALY rate; MIR, mortality-to-incidence ratio; UI, uncertainty interval.

Table S9. Age-standardized rates (per 100,000 population) of incidence, mortality, and disability-adjusted life years and mortality-to-incidence ratio for kidney cancer in 2021 by age group, in low-middle Socio-demographic Index region.

Age	ASIR, per 100,000 population (95% UI)		ASMR, per 100,000 population (95% UI)		ASDR, per 100,000 population (95% UI)		MIR (95% UI)	
	Males	Females	Males	Females	Males	Females	Males	Females
<1 year	0.94 (0.71 to 1.21)	0.85 (0.64 to 1.08)	0.44 (0.33 to 0.57)	0.28 (0.20 to 0.36)	39.62 (29.64 to 51.42)	25.09 (18.64 to 33.18)	0.47 (0.32 to 0.69)	0.32 (0.22 to 0.48)
2-4 years	0.69 (0.48 to 0.92)	0.58 (0.40 to 0.82)	0.32 (0.22 to 0.44)	0.21 (0.14 to 0.30)	28.38 (19.28 to 38.58)	18.05 (12.19 to 26.35)	0.47 (0.29 to 0.77)	0.35 (0.20 to 0.61)
5-9 years	0.18 (0.15 to 0.22)	0.24 (0.18 to 0.32)	0.08 (0.06 to 0.10)	0.07 (0.05 to 0.09)	6.65 (5.35 to 8.30)	5.85 (4.58 to 7.51)	0.44 (0.32 to 0.60)	0.29 (0.20 to 0.43)
10-14 years	0.07 (0.06 to 0.08)	0.09 (0.07 to 0.12)	0.03 (0.02 to 0.04)	0.02 (0.02 to 0.03)	2.34 (1.90 to 2.83)	1.96 (1.56 to 2.59)	0.42 (0.32 to 0.56)	0.29 (0.19 to 0.44)
15-19 years	0.04 (0.04 to 0.05)	0.07 (0.06 to 0.09)	0.02 (0.01 to 0.02)	0.02 (0.02 to 0.03)	1.32 (1.08 to 1.56)	1.54 (1.25 to 1.95)	0.41 (0.31 to 0.52)	0.28 (0.20 to 0.40)
20-24 years	0.07 (0.06 to 0.08)	0.10 (0.08 to 0.13)	0.03 (0.02 to 0.03)	0.03 (0.03 to 0.04)	1.90 (1.58 to 2.21)	2.16 (1.77 to 2.71)	0.42 (0.34 to 0.53)	0.30 (0.22 to 0.42)
25-29 years	0.12 (0.10 to 0.15)	0.15 (0.12 to 0.18)	0.05 (0.04 to 0.06)	0.04 (0.03 to 0.05)	3.10 (2.48 to 3.70)	2.64 (2.20 to 3.26)	0.39 (0.30 to 0.51)	0.28 (0.21 to 0.38)
30-34 years	0.25 (0.20 to 0.30)	0.24 (0.20 to 0.29)	0.10 (0.08 to 0.11)	0.06 (0.05 to 0.08)	5.79 (4.64 to 6.81)	3.82 (3.19 to 4.59)	0.39 (0.30 to 0.51)	0.27 (0.21 to 0.36)
35-39 years	0.53 (0.43 to 0.63)	0.44 (0.36 to 0.53)	0.20 (0.16 to 0.23)	0.11 (0.09 to 0.13)	10.62 (8.60 to 12.53)	5.96 (5.05 to 7.09)	0.37 (0.29 to 0.48)	0.25 (0.19 to 0.32)
40-44 years	1.08 (0.93 to 1.24)	0.69 (0.59 to 0.81)	0.42 (0.36 to 0.49)	0.19 (0.16 to 0.21)	20.72 (17.63 to 23.92)	9.32 (8.02 to 10.72)	0.39 (0.32 to 0.48)	0.27 (0.22 to 0.34)

**45-49 years**	1.91 (1.63 to 2.20)	1.01 (0.88 to 1.17)	0.91 (0.77 to 1.04)	0.36 (0.31 to 0.40)	39.88 (33.92 to 45.75)	15.81 (13.93 to 17.77)	0.48 (0.38 to 0.59)	0.35 (0.29 to 0.43)
**50-54 years**	3.17 (2.76 to 3.61)	1.58 (1.37 to 1.81)	1.69 (1.47 to 1.93)	0.66 (0.58 to 0.75)	66.01 (57.46 to 75.14)	26.02 (22.68 to 29.42)	0.53 (0.44 to 0.65)	0.42 (0.35 to 0.51)
**55-59 years**	4.78 (4.27 to 5.35)	2.29 (2.04 to 2.60)	2.86 (2.55 to 3.22)	1.15 (1.02 to 1.30)	97.89 (87.23 to 110.10)	39.37 (35.00 to 44.39)	0.60 (0.51 to 0.71)	0.50 (0.42 to 0.60)
**60-64 years**	6.37 (5.75 to 7.03)	3.17 (2.81 to 3.55)	4.09 (3.67 to 4.53)	1.77 (1.58 to 1.97)	120.69 (108.31 to 133.93)	52.24 (46.73 to 58.23)	0.64 (0.55 to 0.74)	0.56 (0.47 to 0.65)
**65-69 years**	7.74 (6.94 to 8.59)	3.95 (3.53 to 4.39)	5.43 (4.85 to 6.03)	2.50 (2.25 to 2.79)	134.96 (120.55 to 149.44)	62.30 (56.02 to 69.60)	0.70 (0.60 to 0.82)	0.63 (0.54 to 0.74)
**70-74 years**	9.07 (8.20 to 9.94)	4.74 (4.24 to 5.25)	6.99 (6.29 to 7.65)	3.38 (3.04 to 3.75)	143.05 (128.91 to 156.42)	69.40 (62.32 to 76.99)	0.77 (0.67 to 0.88)	0.71 (0.61 to 0.83)
**75-79 years**	9.81 (8.88 to 10.75)	4.87 (4.37 to 5.40)	8.67 (7.85 to 9.51)	4.05 (3.65 to 4.51)	141.85 (128.68 to 155.27)	66.24 (59.60 to 73.72)	0.88 (0.77 to 1.01)	0.83 (0.72 to 0.97)
**80-84 years**	9.72 (8.66 to 10.76)	4.90 (4.29 to 5.48)	10.16 (9.09 to 11.24)	4.93 (4.32 to 5.53)	130.10 (116.37 to 144.30)	63.01 (55.40 to 71.09)	1.04 (0.90 to 1.22)	1.00 (0.84 to 1.20)
**85-89 years**	9.87 (8.68 to 11.03)	4.63 (3.82 to 5.28)	11.87 (10.51 to 13.28)	5.40 (4.49 to 6.17)	120.51 (106.86 to 134.72)	54.81 (45.58 to 62.52)	1.20 (1.02 to 1.42)	1.17 (0.93 to 1.46)
**90-94 years**	9.38 (7.89 to 10.50)	4.31 (3.38 to 5.10)	13.26 (11.20 to 14.84)	6.02 (4.75 to 7.10)	116.58 (98.68 to 129.95)	52.89 (41.79 to 62.40)	1.41 (1.16 to 1.72)	1.40 (1.05 to 1.86)
**95+ years**	5.96 (4.57 to 7.05)	3.53 (2.45 to 4.33)	9.17 (7.07 to 10.83)	5.37 (3.72 to 6.55)	75.82 (58.63 to 89.65)	43.51 (30.25 to 53.14)	1.54 (1.14 to 2.08)	1.52 (1.03 to 2.24)

Abbreviations: DALYs, disability-adjusted life years; ASIR, age-standardized incidence rate; ASMR, age-standardized mortality rate; ASDR, age-standardized DALY rate; MIR, mortality-to-incidence ratio; UI, uncertainty interval.

Table S10. Age-standardized rates (per 100,000 population) of incidence, mortality, and disability-adjusted life years and mortality-to-incidence ratio for kidney cancer in 2021 by age group, in low Socio-demographic Index region.

Age	ASIR, per 100,000 population (95% UI)		ASMR, per 100,000 population (95% UI)		ASDR, per 100,000 population (95% UI)		MIR (95% UI)	
	Males	Females	Males	Females	Males	Females	Males	Females
<1 year	1.23 (0.74 to 1.79)	1.11 (0.68 to 1.65)	0.67 (0.40 to 0.96)	0.46 (0.29 to 0.70)	60.27 (35.69 to 87.15)	41.84 (26.30 to 63.27)	0.54 (0.28 to 1.05)	0.42 (0.21 to 0.82)
2-4 years	1.29 (0.76 to 1.84)	1.26 (0.79 to 1.81)	0.71 (0.43 to 1.02)	0.58 (0.36 to 0.82)	62.11 (37.54 to 89.10)	50.54 (31.54 to 71.75)	0.55 (0.29 to 1.04)	0.46 (0.25 to 0.85)
5-9 years	0.14 (0.10 to 0.18)	0.21 (0.14 to 0.31)	0.07 (0.05 to 0.10)	0.09 (0.06 to 0.12)	6.26 (4.42 to 8.29)	7.29 (5.03 to 10.16)	0.53 (0.34 to 0.84)	0.41 (0.24 to 0.72)
10-14 years	0.05 (0.03 to 0.06)	0.06 (0.04 to 0.09)	0.03 (0.02 to 0.03)	0.03 (0.02 to 0.04)	2.06 (1.33 to 2.70)	2.00 (1.43 to 2.84)	0.54 (0.33 to 0.87)	0.39 (0.23 to 0.69)
15-19 years	0.02 (0.01 to 0.03)	0.05 (0.03 to 0.08)	0.01 (0.01 to 0.02)	0.02 (0.01 to 0.03)	0.90 (0.55 to 1.28)	1.31 (0.87 to 1.96)	0.53 (0.29 to 0.98)	0.37 (0.19 to 0.84)
20-24 years	0.03 (0.02 to 0.05)	0.07 (0.05 to 0.10)	0.02 (0.01 to 0.03)	0.03 (0.02 to 0.04)	1.32 (0.81 to 1.78)	1.81 (1.30 to 2.49)	0.55 (0.32 to 0.95)	0.39 (0.24 to 0.67)
25-29 years	0.07 (0.04 to 0.10)	0.10 (0.07 to 0.14)	0.04 (0.02 to 0.05)	0.04 (0.03 to 0.05)	2.29 (1.39 to 3.19)	2.29 (1.66 to 3.05)	0.50 (0.28 to 0.90)	0.36 (0.23 to 0.59)
30-34 years	0.16 (0.10 to 0.21)	0.14 (0.10 to 0.19)	0.08 (0.05 to 0.10)	0.05 (0.04 to 0.07)	4.61 (2.91 to 6.07)	2.90 (2.16 to 3.89)	0.49 (0.29 to 0.82)	0.36 (0.23 to 0.57)
35-39 years	0.36 (0.23 to 0.47)	0.25 (0.18 to 0.36)	0.17 (0.11 to 0.22)	0.08 (0.06 to 0.12)	8.89 (5.71 to 11.74)	4.61 (3.34 to 6.28)	0.46 (0.28 to 0.76)	0.34 (0.21 to 0.56)
40-44 years	0.76 (0.51 to 0.98)	0.41 (0.29 to 0.56)	0.37 (0.24 to 0.47)	0.14 (0.11 to 0.19)	17.83 (11.90 to 23.11)	7.12 (5.16 to 9.51)	0.48 (0.30 to 0.77)	0.35 (0.22 to 0.56)

**45-49 years**	1.44 (1.00 to 1.89)	0.71 (0.52 to 0.99)	0.83 (0.57 to 1.08)	0.31 (0.23 to 0.41)	36.14 (24.99 to 46.97)	13.75 (10.18 to 18.22)	0.57 (0.36 to 0.90)	0.44 (0.28 to 0.70)
**50-54 years**	2.41 (1.63 to 3.12)	1.15 (0.86 to 1.50)	1.53 (1.03 to 1.98)	0.60 (0.45 to 0.78)	59.38 (39.90 to 77.20)	23.23 (17.47 to 30.47)	0.63 (0.40 to 1.00)	0.52 (0.34 to 0.78)
**55-59 years**	3.60 (2.56 to 4.53)	1.67 (1.27 to 2.14)	2.51 (1.78 to 3.18)	1.02 (0.78 to 1.29)	85.61 (60.73 to 108.19)	34.85 (26.63 to 44.06)	0.70 (0.47 to 1.05)	0.61 (0.42 to 0.89)
**60-64 years**	5.15 (3.77 to 6.54)	2.63 (1.99 to 3.42)	3.81 (2.78 to 4.84)	1.78 (1.36 to 2.30)	111.86 (81.86 to 142.38)	52.44 (40.00 to 67.71)	0.74 (0.50 to 1.10)	0.68 (0.46 to 1.00)
**65-69 years**	6.34 (4.74 to 7.87)	3.41 (2.59 to 4.29)	5.02 (3.72 to 6.22)	2.52 (1.93 to 3.18)	124.34 (92.32 to 154.24)	62.35 (47.70 to 78.71)	0.79 (0.55 to 1.13)	0.74 (0.51 to 1.06)
**70-74 years**	7.47 (5.67 to 9.17)	4.26 (3.27 to 5.35)	6.40 (4.85 to 7.88)	3.46 (2.68 to 4.36)	130.70 (99.09 to 160.60)	70.75 (54.81 to 89.17)	0.86 (0.61 to 1.20)	0.81 (0.57 to 1.16)
**75-79 years**	8.92 (6.63 to 10.89)	4.70 (3.62 to 5.86)	8.61 (6.37 to 10.51)	4.36 (3.38 to 5.49)	140.80 (104.10 to 171.17)	71.30 (55.14 to 90.08)	0.97 (0.68 to 1.37)	0.93 (0.66 to 1.32)
**80-84 years**	8.67 (6.74 to 10.45)	4.89 (3.63 to 6.43)	9.64 (7.50 to 11.64)	5.31 (3.95 to 7.04)	123.49 (95.79 to 148.60)	67.89 (50.59 to 89.97)	1.11 (0.82 to 1.51)	1.09 (0.71 to 1.66)
**85-89 years**	9.21 (7.08 to 11.38)	4.55 (3.30 to 6.20)	11.54 (8.89 to 14.27)	5.61 (4.09 to 7.64)	117.33 (90.45 to 145.11)	57.07 (41.58 to 77.41)	1.25 (0.89 to 1.76)	1.23 (0.77 to 1.97)
**90-94 years**	8.60 (6.55 to 10.54)	3.99 (2.80 to 5.52)	12.33 (9.44 to 15.07)	5.69 (4.00 to 7.90)	108.48 (83.28 to 132.70)	50.04 (35.07 to 69.47)	1.43 (1.03 to 2.00)	1.43 (0.85 to 2.36)
**95+ years**	4.24 (2.83 to 5.58)	2.37 (1.62 to 3.41)	6.65 (4.46 to 8.74)	3.69 (2.51 to 5.29)	56.03 (37.60 to 73.68)	31.00 (21.11 to 44.50)	1.57 (0.97 to 2.53)	1.56 (0.88 to 2.75)

Abbreviations: DALYs, disability-adjusted life years; ASIR, age-standardized incidence rate; ASMR, age-standardized mortality rate; ASDR, age-standardized DALY rate; MIR, mortality-to-incidence ratio; UI, uncertainty interval.

Table S11. Counts and age-standardized rates (per 100,000 population) of incidence, mortality, and disability-adjusted life years and mortality-to-incidence ratio for kidney cancer in 2021, globally and by regions.

	Incidence		Mortality		DALYs		
	Count (95% UI)	ASR, per 100,000 population (95% UI)	Count (95% UI)	ASR, per 100,000 population (95% UI)	Count (95% UI)	ASR, per 100,000 population (95% UI)	MIR (95% UI)
**Global**	387,828.72 (365,359.71 to 406,635.25)	4.52 (4.26 to 4.75)	161,194.54 (150,317.57 to 169,348.28)	1.91 (1.78 to 2.01)	4,016,362.12 (3,806,832.21 to 4,246,783.03)	47.33 (44.76 to 50.07)	0.42 (0.39 to 0.46)
**Sex**							
Male	252,589.19 (237,746.50 to 268,143.49)	6.26 (5.88 to 6.64)	106,543.65 (99,856.60 to 112,786.67)	2.79 (2.61 to 2.95)	2,738,077.26 (2,581,071.77 to 2,925,663.38)	67.79 (63.83 to 72.46)	0.45 (0.41 to 0.49)
Female	135,239.53 (124,078.89 to 144,234.80)	3.00 (2.76 to 3.20)	54,650.89 (48,631.49 to 58,643.21)	1.19 (1.06 to 1.28)	1,278,284.86 (1,176,214.64 to 1,371,879.89)	28.91 (26.54 to 31.21)	0.40 (0.35 to 0.44)
**SDI regions**							
High SDI	173,289.72 (161,693.15 to 180,828.97)	8.97 (8.46 to 9.33)	65,032.57 (58,999.09 to 68,540.11)	2.98 (2.74 to 3.12)	1,345,338.88 (1,259,629.25 to 1,408,119.30)	69.99 (66.59 to 73.07)	0.33 (0.31 to 0.36)
High-middle SDI	119,118.88 (111,198.35 to 127,043.89)	6.27 (5.84 to 6.69)	46,847.85 (43,590.15 to 49,875.18)	2.39 (2.23 to 2.55)	1,156,099.26 (1,078,825.58 to 1,235,074.94)	61.16 (57.09 to 65.23)	0.38 (0.35 to 0.42)
Middle SDI	66,679.59 (60,781.67 to 73,141.40)	2.46 (2.25 to 2.69)	31,805.86 (29,213.18 to 34,554.79)	1.22 (1.12 to 1.32)	901,500.01 (831,893.88 to 976,433.21)	33.47 (30.84 to 36.15)	0.49 (0.44 to 0.56)
Low-middle SDI	20,486.88 (18,629.11 to 22,489.93)	1.33 (1.22 to 1.45)	12,273.78 (11,332.25 to 13,364.54)	0.86 (0.79 to 0.93)	390,480.92 (348,567.54 to 434,565.66)	24.46 (22.12 to 27.02)	0.64 (0.57 to 0.72)
Low SDI	7823.35 (5608.43 to 9888.67)	1.15 (0.87 to 1.40)	5019.99 (3681.53 to 6242.71)	0.85 (0.65 to 1.04)	217,858.11 (149,898.20 to 286,746.52)	26.00 (18.96 to 32.50)	0.74 (0.53 to 1.03)
**Central Europe, Eastern Europe, and Central Asia**	54,172.67 (50,942.10 to 57,371.34)	8.55 (8.04 to 9.05)	25,333.27 (23,772.40 to 26,803.80)	3.88 (3.64 to 4.11)	625,250.16 (585,997.46 to 663,510.11)	100.14 (93.74 to 106.23)	0.45 (0.42 to 0.49)

Central Asia	3451.40 (2990.13 to 3881.41)	3.90 (3.39 to 4.38)	1870.76 (1623.57 to 2108.42)	2.25 (1.95 to 2.52)	57,945.94 (49,996.63 to 65,311.34)	63.85 (55.23 to 71.86)	0.58 (0.48 to 0.69)
Central Europe	18,116.82 (16,637.85 to 19,653.48)	8.63 (7.91 to 9.37)	9468.08 (8677.23 to 10,217.67)	4.22 (3.87 to 4.56)	212,304.70 (195,928.56 to 229,137.89)	102.79 (94.92 to 111.10)	0.49 (0.43 to 0.55)
Eastern Europe	32,604.45 (29,917.59 to 35,234.83)	9.61 (8.82 to 10.40)	13,994.43 (12,813.39 to 15,168.20)	3.97 (3.64 to 4.31)	354,999.52 (323,642.50 to 387,672.48)	105.76 (96.57 to 115.64)	0.41 (0.37 to 0.46)
**High-income regions**	187,326.88 (174,352.93 to 195,243.45)	9.52 (8.99 to 9.87)	68,355.77 (61,967.24 to 72,188.16)	2.99 (2.75 to 3.13)	1,400,271.11 (1,304,885.71 to 1,465,267.72)	70.97 (67.21 to 73.88)	0.31 (0.29 to 0.34)
Australasia	4218.55 (3715.57 to 4727.88)	8.55 (7.57 to 9.55)	1580.24 (1380.38 to 1779.41)	2.87 (2.51 to 3.22)	33,351.15 (29,575.73 to 37,243.11)	67.67 (60.29 to 75.54)	0.34 (0.28 to 0.40)
High-income Asia Pacific	19,388.08 (17,328.09 to 20,711.74)	4.58 (4.20 to 4.86)	10,002.67 (8550.49 to 10,811.09)	1.89 (1.68 to 2.02)	174,477.78 (156,186.00 to 186,096.43)	41.24 (38.15 to 43.59)	0.41 (0.37 to 0.46)
High-income North America	70,801.11 (65,828.30 to 73,663.32)	11.54 (10.82 to 11.96)	20,238.26 (18,356.94 to 21,212.13)	3.04 (2.78 to 3.17)	453,389.71 (425,275.66 to 473,506.04)	74.46 (70.22 to 77.61)	0.26 (0.24 to 0.29)
Southern Latin America	11,016.18 (10,041.81 to 12,054.65)	13.44 (12.27 to 14.73)	3959.55 (3587.69 to 4334.82)	4.55 (4.12 to 4.97)	100,201.79 (91,403.99 to 109,044.73)	120.33 (109.84 to 130.97)	0.34 (0.30 to 0.39)
Western Europe	81,902.97 (75,725.63 to 87,160.01)	9.74 (9.11 to 10.36)	32,575.04 (29,488.77 to 34,955.43)	3.28 (3.01 to 3.50)	638,850.68 (594,117.05 to 679,181.04)	75.82 (71.42 to 80.46)	0.34 (0.31 to 0.37)
**Latin America and Caribbean**	23,407.66 (21,713.35 to 25,165.19)	3.74 (3.47 to 4.02)	13,378.47 (12,346.79 to 14,360.39)	2.17 (2.00 to 2.33)	369,335.02 (342,657.10 to 398,891.28)	59.08 (54.71 to 63.80)	0.58 (0.52 to 0.64)
Andean Latin America	2158.97 (1722.08 to 2673.28)	3.56 (2.85 to 4.41)	1224.52 (981.94 to 1501.33)	2.08 (1.67 to 2.56)	33,893.31 (27,347.62 to 42,066.88)	55.46 (44.65 to 68.78)	0.58 (0.43 to 0.80)
Caribbean	1613.62 (1417.65 to 1808.49)	3.06 (2.69 to 3.44)	886.90 (776.28 to 996.96)	1.67 (1.46 to 1.87)	25,081.70 (21,912.99 to 28,469.98)	48.57 (42.24 to 55.71)	0.54 (0.46 to 0.65)
Central Latin America	10,899.16 (9767.38 to 12,183.03)	4.27 (3.82 to 4.76)	6143.88 (5509.78 to 6843.80)	2.46 (2.21 to 2.73)	172,174.04 (153,715.83 to 193,472.33)	67.13 (59.97 to 75.34)	0.58 (0.49 to 0.67)

Tropical Latin America	8735.90 (8186.48 to 9211.03)	3.41 (3.19 to 3.59)	5123.17 (4755.71 to 5409.13)	2.02 (1.87 to 2.13)	138,185.97 (130,615.38 to 144,983.57)	54.17 (51.10 to 56.95)	0.59 (0.54 to 0.65)
**North Africa and Middle East**	14,301.48 (12,685.48 to 16,109.62)	2.82 (2.51 to 3.16)	5464.09 (4846.74 to 6183.06)	1.25 (1.11 to 1.43)	165,276.43 (146,557.66 to 186,215.67)	32.81 (29.06 to 36.93)	0.44 (0.37 to 0.53)
**South Asia**	15,813.71 (14,358.95 to 17,338.21)	1.03 (0.94 to 1.13)	10,270.92 (9340.53 to 11,250.31)	0.71 (0.64 to 0.78)	304,759.28 (275,345.09 to 336,310.98)	19.46 (17.60 to 21.45)	0.69 (0.60 to 0.78)
**Southeast Asia, East Asia, and Oceania**	83,732.92 (71,325.90 to 97,763.24)	3.02 (2.58 to 3.50)	32,616.83 (27,993.79 to 37,868.93)	1.20 (1.04 to 1.39)	896,130.33 (770,883.18 to 1,033,411.13)	32.94 (28.53 to 37.73)	0.40 (0.32 to 0.49)
East Asia	70,149.86 (57,904.21 to 84,101.30)	3.41 (2.83 to 4.05)	26,321.96 (21,803.62 to 31,311.51)	1.27 (1.06 to 1.50)	699,886.74 (576,742.20 to 835,911.74)	34.77 (29.00 to 41.18)	0.37 (0.29 to 0.48)
Oceania	57.03 (36.55 to 80.97)	0.64 (0.41 to 0.92)	31.26 (19.21 to 45.79)	0.41 (0.25 to 0.60)	1150.47 (725.79 to 1641.41)	11.50 (7.14 to 16.86)	0.64 (0.33 to 1.18)
Southeast Asia	13,526.03 (12,006.23 to 15,221.67)	1.96 (1.74 to 2.20)	6263.62 (5542.22 to 6987.16)	0.97 (0.86 to 1.08)	195,093.12 (171,890.15 to 217,411.94)	28.03 (24.72 to 31.24)	0.49 (0.42 to 0.58)
**Sub-Saharan Africa**	9073.40 (6602.59 to 11,360.47)	1.38 (1.07 to 1.66)	5775.20 (4292.37 to 7149.10)	1.02 (0.81 to 1.22)	255,339.79 (177,467.00 to 331,342.93)	30.80 (22.89 to 38.14)	0.74 (0.55 to 1.01)
Central Sub-Saharan Africa	628.73 (390.05 to 958.37)	0.95 (0.58 to 1.48)	432.88 (265.31 to 669.75)	0.74 (0.45 to 1.18)	15,853.20 (9889.37 to 23,813.37)	20.48 (12.55 to 31.70)	0.79 (0.36 to 1.67)
Eastern Sub-Saharan Africa	3854.03 (2536.77 to 5071.07)	1.72 (1.20 to 2.18)	2519.56 (1698.38 to 3279.12)	1.31 (0.93 to 1.65)	105,318.12 (65,421.70 to 143,774.74)	37.82 (25.44 to 49.07)	0.76 (0.51 to 1.15)
Southern Sub-Saharan Africa	1331.58 (1189.85 to 1462.89)	2.13 (1.92 to 2.33)	828.84 (747.17 to 904.90)	1.45 (1.31 to 1.56)	26,408.66 (23,582.71 to 29,226.07)	40.36 (36.28 to 44.41)	0.68 (0.60 to 0.77)
Western Sub-Saharan Africa	3259.05 (2309.18 to 4196.78)	0.98 (0.78 to 1.20)	1993.93 (1496.56 to 2494.18)	0.72 (0.59 to 0.86)	107,759.81 (73,333.92 to 141,104.71)	24.32 (18.31 to 30.31)	0.73 (0.55 to 0.99)

Abbreviations: ASR, age-standardized rate; DALYs, Disability-Adjusted Life Years; UI, uncertainty interval; MIR, mortality-incidence ratio; SDI, Socio-demographic Index.

Table S12. Age-standardized mortality rates, per 10,000,000 population attributable due to high body-mass index in 1990 and 2021, globally and by regions.

	Both (95% UI)		Males (95% UI)		Females (95% UI)	
	1990	2021	1990	2021	1990	2021
**Global**	31.64 (12.40 to 51.73)	37.99 (15.42 to 61.92)	40.89 (15.83 to 67.03)	52.92 (21.39 to 85.96)	24.08 (9.43 to 39.45)	25.50 (10.30 to 40.92)
**SDI regions**						
High SDI	61.19 (24.07 to 99.73)	67.15 (27.54 to 107.86)	87.00 (33.99 to 142.69)	97.32 (39.94 to 155.73)	41.66 (16.36 to 68.35)	41.59 (16.71 to 66.63)
High-middle SDI	40.04 (15.69 to 65.31)	51.68 (20.77 to 84.13)	52.98 (20.47 to 87.00)	72.77 (29.01 to 119.31)	30.53 (12.00 to 49.68)	34.61 (13.94 to 55.13)
Middle SDI	9.67 (3.74 to 15.76)	20.71 (8.51 to 34.14)	11.67 (4.44 to 18.96)	28.00 (11.65 to 46.50)	7.90 (3.12 to 12.92)	14.34 (5.79 to 23.25)
Low-middle SDI	4.66 (1.80 to 7.52)	11.65 (4.59 to 19.03)	5.01 (1.89 to 8.01)	14.71 (5.81 to 24.06)	4.31 (1.72 to 6.91)	8.87 (3.44 to 14.31)
Low SDI	3.71 (1.39 to 5.96)	7.47 (2.62 to 12.47)	4.12 (1.57 to 6.81)	9.39 (3.22 to 15.68)	3.30 (1.23 to 5.60)	5.65 (2.08 to 9.52)
**Central Europe, Eastern Europe, and Central Asia**	64.41 (25.07 to 104.28)	97.01 (39.73 to 155.90)	91.83 (34.98 to 150.23)	143.25 (57.82 to 234.89)	47.33 (18.89 to 77.13)	64.73 (26.76 to 101.83)
Central Asia	32.75 (13.03 to 53.33)	50.33 (19.92 to 82.76)	37.43 (13.88 to 63.99)	69.34 (27.02 to 116.39)	29.11 (11.58 to 47.95)	35.42 (14.53 to 57.45)
Central Europe	75.54 (29.65 to 124.95)	105.97 (43.29 to 174.99)	108.91 (42.35 to 180.41)	154.58 (62.56 to 252.70)	50.22 (20.05 to 82.21)	68.21 (28.59 to 111.71)
Eastern Europe	63.68 (24.70 to 102.85)	100.60 (40.64 to 161.35)	89.90 (33.86 to 146.97)	151.07 (60.48 to 249.79)	48.96 (19.70 to 78.89)	67.57 (27.71 to 105.17)

**High-income regions**	60.25 (23.69 to 98.06)	68.11 (27.65 to 108.84)	86.07 (33.71 to 141.32)	100.35 (40.79 to 159.34)	40.70 (15.92 to 66.30)	41.10 (16.32 to 66.10)
Australasia	61.87 (23.78 to 102.15)	74.31 (30.65 to 120.45)	80.80 (31.55 to 133.47)	110.52 (45.58 to 177.44)	46.53 (17.42 to 76.31)	42.42 (16.89 to 68.68)
High-income Asia Pacific	13.99 (5.32 to 22.87)	21.07 (7.95 to 34.33)	20.69 (7.91 to 33.85)	31.83 (12.31 to 51.10)	8.81 (3.29 to 14.39)	12.06 (4.24 to 19.77)
High-income North America	75.56 (30.15 to 122.84)	84.77 (35.31 to 132.30)	106.35 (42.41 to 171.90)	124.84 (52.11 to 193.59)	51.33 (20.47 to 84.25)	50.86 (20.78 to 79.36)
Southern Latin America	78.60 (31.15 to 129.31)	118.32 (46.42 to 185.49)	103.12 (40.56 to 172.70)	177.20 (67.30 to 285.67)	57.71 (22.84 to 93.88)	71.26 (28.78 to 110.92)
Western Europe	65.78 (25.84 to 106.97)	72.71 (29.08 to 118.88)	96.00 (37.37 to 156.72)	106.73 (42.74 to 173.91)	43.69 (17.12 to 70.93)	44.65 (17.37 to 74.07)
**Latin America and Caribbean**	27.07 (10.64 to 44.47)	51.73 (21.18 to 84.67)	29.70 (11.63 to 49.37)	68.60 (27.81 to 111.08)	24.67 (9.74 to 40.42)	37.46 (15.38 to 60.55)
Andean Latin America	29.02 (11.10 to 48.68)	45.45 (18.80 to 76.84)	26.93 (10.19 to 45.88)	54.44 (22.51 to 92.47)	31.06 (12.16 to 50.99)	37.31 (15.51 to 63.98)
Caribbean	20.80 (8.04 to 34.56)	33.59 (13.70 to 54.67)	23.07 (8.90 to 37.11)	42.89 (17.41 to 71.62)	18.68 (7.25 to 31.55)	25.41 (10.07 to 40.32)
Central Latin America	33.86 (13.26 to 55.31)	63.00 (26.51 to 100.83)	35.58 (13.77 to 58.70)	82.96 (35.10 to 133.72)	32.26 (12.86 to 51.99)	45.90 (19.38 to 73.87)
Tropical Latin America	22.29 (8.85 to 37.20)	45.94 (18.16 to 74.61)	26.80 (10.69 to 44.97)	63.50 (24.99 to 104.10)	18.31 (7.22 to 30.04)	31.82 (12.75 to 51.56)
**North Africa and Middle East**	16.79 (6.55 to 26.79)	33.17 (13.95 to 52.91)	20.17 (7.69 to 32.24)	44.13 (18.40 to 72.78)	13.37 (5.29 to 21.35)	21.96 (8.96 to 34.51)

**South Asia**	1.98 (0.74 to 3.14)	6.08 (2.24 to 9.94)	2.63 (0.96 to 4.23)	8.05 (2.99 to 13.23)	1.27 (0.48 to 2.12)	4.22 (1.52 to 7.07)
**Southeast Asia, East Asia, and Oceania**	6.12 (2.37 to 10.17)	15.94 (5.95 to 27.31)	8.02 (3.04 to 13.28)	22.26 (8.30 to 38.64)	4.50 (1.70 to 7.54)	10.59 (4.16 to 18.34)
East Asia	6.81 (2.66 to 11.43)	17.78 (6.61 to 31.33)	9.10 (3.54 to 15.28)	25.29 (9.59 to 44.49)	4.94 (1.89 to 8.39)	11.42 (4.52 to 20.26)
Oceania	5.00 (1.72 to 8.84)	6.93 (2.52 to 12.17)	8.59 (2.97 to 15.24)	11.89 (4.30 to 21.40)	1.31 (0.50 to 2.22)	1.74 (0.71 to 2.84)
Southeast Asia	4.20 (1.59 to 6.75)	9.61 (3.61 to 16.17)	5.32 (1.99 to 8.64)	11.97 (4.47 to 19.74)	3.23 (1.17 to 5.20)	7.52 (2.84 to 12.54)
**Sub-Saharan Africa**	6.24 (2.26 to 9.95)	13.73 (5.06 to 21.94)	7.24 (2.63 to 12.06)	17.71 (6.39 to 29.15)	5.32 (1.95 to 8.53)	10.36 (3.94 to 16.93)
Central Sub-Saharan Africa	4.08 (1.38 to 7.48)	9.55 (3.11 to 18.56)	6.17 (2.02 to 11.93)	16.59 (5.47 to 33.22)	2.24 (0.68 to 4.04)	4.17 (1.33 to 8.46)
Eastern Sub-Saharan Africa	5.84 (2.11 to 9.37)	12.57 (4.26 to 21.33)	5.94 (2.20 to 9.87)	14.09 (4.64 to 24.24)	5.74 (2.09 to 9.48)	11.21 (4.01 to 19.88)
Southern Sub-Saharan Africa	15.92 (5.83 to 25.60)	33.43 (13.50 to 52.72)	20.08 (7.21 to 33.16)	46.40 (18.38 to 75.32)	13.00 (4.96 to 21.36)	24.95 (10.31 to 39.32)
Western Sub-Saharan Africa	3.86 (1.38 to 6.45)	9.75 (3.61 to 15.86)	4.90 (1.67 to 8.36)	13.52 (5.13 to 22.60)	2.83 (1.06 to 4.72)	6.42 (2.24 to 10.83)

Abbreviations: UI, uncertainty interval; SDI, Socio-demographic Index.

Table S13. Age-standardized disability-adjusted life year rates, per 100,000 population attributable due to high body-mass index in 1990 and 2021, globally and by regions.

	Both (95% UI)		Males (95% UI)		Females (95% UI)	
	**1990**	**2021**	**1990**	**2021**	**1990**	**2021**
**Global**	7.80 (3.06 to 12.69)	8.99 (3.68 to 14.51)	10.10 (3.92 to 16.46)	12.52 (5.09 to 20.38)	5.74 (2.27 to 9.33)	5.81 (2.34 to 9.20)
**SDI regions**						
High SDI	15.65 (6.18 to 25.42)	16.15 (6.70 to 25.64)	22.13 (8.70 to 36.13)	23.32 (9.69 to 37.29)	10.22 (4.04 to 16.65)	9.59 (3.95 to 15.28)
High-middle SDI	10.51 (4.12 to 17.16)	12.88 (5.23 to 20.90)	13.77 (5.32 to 22.66)	18.17 (7.29 to 29.74)	7.80 (3.10 to 12.62)	8.18 (3.30 to 12.94)
Middle SDI	2.57 (0.99 to 4.20)	5.50 (2.25 to 9.03)	3.07 (1.18 to 5.00)	7.39 (3.04 to 12.19)	2.09 (0.83 to 3.41)	3.75 (1.52 to 6.05)
Low-middle SDI	1.24 (0.48 to 2.02)	3.10 (1.23 to 5.03)	1.33 (0.50 to 2.15)	3.91 (1.56 to 6.38)	1.15 (0.46 to 1.86)	2.35 (0.91 to 3.76)
Low SDI	0.99 (0.37 to 1.62)	1.99 (0.70 to 3.33)	1.09 (0.41 to 1.80)	2.49 (0.84 to 4.21)	0.90 (0.34 to 1.54)	1.50 (0.56 to 2.55)
**Central Europe, Eastern Europe, and Central Asia**	17.55 (6.83 to 28.33)	24.50 (10.08 to 39.03)	24.60 (9.37 to 40.18)	36.24 (14.69 to 58.90)	12.57 (5.03 to 20.35)	15.52 (6.35 to 24.31)
Central Asia	9.21 (3.65 to 15.03)	13.78 (5.41 to 22.71)	10.69 (3.98 to 18.14)	19.04 (7.38 to 31.83)	8.03 (3.22 to 13.25)	9.42 (3.85 to 15.32)
Central Europe	19.43 (7.64 to 32.11)	25.63 (10.47 to 42.14)	28.24 (11.05 to 46.70)	37.50 (15.15 to 61.16)	12.18 (4.85 to 19.89)	15.59 (6.54 to 25.60)
Eastern Europe	17.97 (6.96 to 28.94)	26.28 (10.65 to 42.07)	24.72 (9.30 to 40.32)	39.64 (15.85 to 65.22)	13.53 (5.50 to 21.66)	16.64 (6.75 to 25.63)

**High-income regions**	15.48 (6.12 to 25.11)	16.55 (6.83 to 26.25)	22.02 (8.68 to 36.00)	24.26 (10.00 to 38.42)	10.03 (3.95 to 16.28)	9.62 (3.96 to 15.28)
Australasia	15.50 (5.96 to 25.58)	17.79 (7.29 to 28.35)	20.21 (7.91 to 33.45)	26.67 (10.90 to 42.58)	11.27 (4.20 to 18.53)	9.59 (3.90 to 15.30)
High-income Asia Pacific	3.53 (1.34 to 5.78)	4.74 (1.80 to 7.60)	5.27 (2.01 to 8.62)	7.20 (2.75 to 11.57)	2.07 (0.77 to 3.39)	2.50 (0.92 to 4.04)
High-income North America	20.57 (8.28 to 33.31)	21.08 (8.89 to 32.52)	28.90 (11.64 to 46.66)	31.02 (13.09 to 47.82)	13.50 (5.41 to 21.87)	12.19 (5.05 to 18.61)
Southern Latin America	21.82 (8.64 to 35.82)	31.26 (12.33 to 48.63)	29.56 (11.61 to 49.33)	46.32 (17.70 to 72.86)	14.97 (5.97 to 24.24)	18.40 (7.48 to 28.80)
Western Europe	16.38 (6.47 to 26.54)	16.73 (6.70 to 27.28)	23.70 (9.29 to 38.66)	24.41 (9.81 to 39.61)	10.42 (4.10 to 16.82)	9.88 (3.92 to 16.13)
**Latin America and Caribbean**	7.38 (2.92 to 12.06)	13.83 (5.71 to 22.57)	8.09 (3.19 to 13.44)	18.26 (7.46 to 29.52)	6.72 (2.67 to 10.96)	9.97 (4.17 to 15.93)
Andean Latin America	8.15 (3.12 to 13.63)	12.07 (5.05 to 20.37)	7.37 (2.83 to 12.59)	14.37 (5.89 to 24.73)	8.91 (3.44 to 14.65)	9.92 (4.22 to 16.83)
Caribbean	5.90 (2.30 to 9.72)	9.22 (3.78 to 14.97)	6.48 (2.52 to 10.42)	11.50 (4.70 to 18.95)	5.35 (2.10 to 8.99)	7.16 (2.88 to 11.33)
Central Latin America	9.11 (3.58 to 14.90)	17.09 (7.20 to 27.12)	9.60 (3.74 to 15.80)	22.59 (9.57 to 36.35)	8.65 (3.44 to 13.96)	12.28 (5.21 to 19.49)
Tropical Latin America	6.06 (2.42 to 10.07)	11.97 (4.77 to 19.32)	7.33 (2.93 to 12.24)	16.32 (6.45 to 26.40)	4.91 (1.96 to 8.04)	8.28 (3.33 to 13.38)
**North Africa and Middle East**	4.39 (1.74 to 7.08)	8.37 (3.56 to 13.33)	5.24 (2.02 to 8.44)	11.17 (4.66 to 18.11)	3.52 (1.41 to 5.61)	5.45 (2.28 to 8.61)

**South Asia**	0.53 (0.20 to 0.85)	1.62 (0.60 to 2.63)	0.70 (0.25 to 1.12)	2.15 (0.81 to 3.52)	0.34 (0.13 to 0.58)	1.11 (0.40 to 1.83)
**Southeast Asia, East Asia, and Oceania**	1.63 (0.64 to 2.69)	4.22 (1.56 to 7.32)	2.13 (0.81 to 3.49)	5.90 (2.22 to 10.12)	1.16 (0.44 to 1.93)	2.65 (1.04 to 4.55)
East Asia	1.77 (0.70 to 2.95)	4.71 (1.75 to 8.30)	2.33 (0.90 to 3.92)	6.72 (2.57 to 11.71)	1.23 (0.46 to 2.09)	2.80 (1.11 to 4.94)
Oceania	1.40 (0.48 to 2.45)	1.93 (0.70 to 3.46)	2.41 (0.84 to 4.30)	3.37 (1.19 to 6.18)	0.31 (0.12 to 0.52)	0.40 (0.17 to 0.66)
Southeast Asia	1.22 (0.47 to 1.97)	2.76 (1.03 to 4.60)	1.53 (0.57 to 2.48)	3.46 (1.28 to 5.65)	0.94 (0.35 to 1.51)	2.13 (0.80 to 3.53)
**Sub-Saharan Africa**	1.62 (0.60 to 2.60)	3.48 (1.29 to 5.60)	1.85 (0.67 to 3.04)	4.50 (1.60 to 7.38)	1.40 (0.52 to 2.24)	2.60 (0.98 to 4.29)
Central Sub-Saharan Africa	1.08 (0.37 to 1.99)	2.56 (0.84 to 4.91)	1.63 (0.54 to 3.15)	4.28 (1.47 to 8.59)	0.60 (0.18 to 1.06)	1.12 (0.36 to 2.21)
Eastern Sub-Saharan Africa	1.56 (0.56 to 2.50)	3.31 (1.10 to 5.67)	1.57 (0.59 to 2.63)	3.74 (1.24 to 6.40)	1.54 (0.56 to 2.54)	2.91 (1.03 to 5.22)
Southern Sub-Saharan Africa	4.33 (1.58 to 6.94)	8.64 (3.49 to 13.54)	5.21 (1.85 to 8.48)	11.59 (4.51 to 18.61)	3.69 (1.42 to 6.07)	6.53 (2.71 to 10.30)
Western Sub-Saharan Africa	0.98 (0.35 to 1.63)	2.40 (0.88 to 3.94)	1.23 (0.42 to 2.13)	3.35 (1.25 to 5.58)	0.70 (0.26 to 1.15)	1.57 (0.55 to 2.69)

Abbreviations: DALYs, Disability-Adjusted Life Years; UI, uncertainty interval; SDI, Socio-demographic Index.

Table S14. Age-standardized mortality rates, per 10,000,000 population attributable due to tobacco in 1990 and 2021, globally and by regions.

	Both (95% UI)		Males (95% UI)		Females (95% UI)	
	1990	2021	1990	2021	1990	2021
**Global**	24.93 (15.55 to 35.24)	18.87 (11.22 to 27.16)	46.46 (29.13 to 65.80)	36.18 (21.58 to 51.80)	7.73 (4.65 to 11.16)	4.49 (2.58 to 6.95)
**SDI regions**						
High SDI	48.95 (30.31 to 69.44)	32.73 (18.80 to 48.78)	88.63 (54.79 to 125.80)	57.64 (33.05 to 85.80)	19.71 (11.99 to 28.20)	11.97 (6.86 to 18.40)
High-middle SDI	29.47 (18.97 to 40.87)	27.33 (16.79 to 38.15)	63.83 (41.01 to 88.83)	56.69 (34.55 to 79.25)	4.53 (2.74 to 6.44)	3.75 (2.25 to 5.43)
Middle SDI	9.72 (6.31 to 13.34)	10.59 (6.49 to 14.70)	19.05 (12.40 to 26.10)	21.62 (13.30 to 30.18)	1.61 (0.97 to 2.27)	1.20 (0.71 to 1.84)
Low-middle SDI	5.04 (3.14 to 7.08)	5.41 (3.34 to 7.78)	9.28 (5.81 to 13.08)	10.73 (6.64 to 15.22)	0.77 (0.44 to 1.12)	0.71 (0.40 to 1.11)
Low SDI	2.33 (1.34 to 3.42)	2.24 (1.25 to 3.34)	4.29 (2.46 to 6.32)	4.20 (2.34 to 6.24)	0.38 (0.21 to 0.60)	0.42 (0.21 to 0.66)
**Central Europe, Eastern Europe, and Central Asia**	39.93 (26.01 to 55.55)	44.26 (27.78 to 61.65)	96.63 (62.77 to 135.44)	100.02 (62.45 to 139.84)	5.01 (3.11 to 7.16)	6.04 (3.73 to 8.70)
Central Asia	15.33 (9.50 to 22.44)	21.91 (14.18 to 31.15)	37.26 (23.12 to 55.00)	50.62 (32.78 to 71.91)	0.67 (0.40 to 1.02)	0.55 (0.33 to 0.82)
Central Europe	50.57 (32.47 to 70.64)	48.86 (29.55 to 70.84)	100.89 (64.72 to 140.05)	94.39 (56.58 to 137.85)	13.13 (8.11 to 18.74)	14.12 (8.68 to 20.50)
Eastern Europe	38.28 (25.22 to 53.27)	46.34 (29.45 to 64.56)	104.47 (68.27 to 147.34)	115.52 (72.83 to 161.84)	1.81 (1.10 to 2.65)	2.65 (1.59 to 3.80)

**High-income regions**	48.23 (29.82 to 68.47)	32.28 (18.57 to 48.42)	87.79 (54.04 to 124.40)	56.94 (32.36 to 85.91)	19.12 (11.65 to 27.51)	11.90 (6.78 to 18.21)
Australasia	37.26 (22.22 to 55.37)	20.09 (10.73 to 32.80)	58.30 (33.79 to 87.75)	32.59 (17.24 to 52.88)	19.96 (12.14 to 29.75)	8.97 (4.98 to 14.84)
High-income Asia Pacific	26.76 (17.51 to 36.61)	19.56 (11.82 to 28.36)	58.17 (38.10 to 78.88)	40.21 (24.19 to 57.82)	3.99 (2.40 to 5.90)	3.06 (1.61 to 5.09)
High-income North America	53.56 (32.07 to 76.56)	35.27 (19.32 to 55.39)	88.07 (52.55 to 126.02)	56.99 (31.14 to 88.52)	26.88 (16.10 to 38.77)	16.84 (9.14 to 26.40)
Southern Latin America	39.64 (23.65 to 57.57)	39.55 (22.48 to 59.97)	71.05 (41.45 to 104.01)	74.23 (41.17 to 112.98)	13.96 (8.14 to 20.66)	11.90 (7.13 to 18.23)
Western Europe	54.05 (33.47 to 75.95)	35.78 (20.74 to 53.09)	101.53 (62.56 to 142.52)	63.79 (36.36 to 95.06)	20.29 (12.18 to 29.35)	12.70 (7.40 to 19.05)
**Latin America and Caribbean**	14.40 (8.93 to 20.41)	11.88 (6.62 to 17.90)	24.16 (15.01 to 34.40)	21.57 (12.11 to 32.38)	5.70 (3.34 to 8.30)	3.96 (2.12 to 6.13)
Andean Latin America	4.82 (2.69 to 7.47)	5.39 (2.89 to 8.91)	8.83 (4.91 to 13.81)	10.57 (5.69 to 17.53)	1.14 (0.60 to 1.82)	0.83 (0.42 to 1.44)
Caribbean	13.00 (7.69 to 19.51)	12.36 (7.14 to 18.74)	22.17 (13.11 to 32.76)	22.59 (12.80 to 34.53)	4.54 (2.58 to 7.05)	3.45 (1.92 to 5.50)
Central Latin America	11.18 (6.65 to 16.03)	7.60 (4.40 to 11.40)	19.44 (11.56 to 27.77)	14.90 (8.59 to 22.47)	3.63 (2.12 to 5.41)	1.56 (0.87 to 2.36)
Tropical Latin America	19.87 (12.20 to 28.27)	17.39 (9.65 to 26.91)	32.67 (20.39 to 47.00)	30.69 (17.16 to 47.76)	8.87 (5.08 to 12.97)	7.00 (3.71 to 11.06)
**North Africa and Middle East**	12.21 (7.31 to 17.25)	13.39 (8.20 to 19.67)	23.34 (13.98 to 32.94)	25.70 (15.79 to 37.83)	1.02 (0.56 to 1.57)	0.97 (0.53 to 1.50)

**South Asia**	4.18 (2.61 to 5.92)	3.83 (2.38 to 5.35)	7.72 (4.84 to 10.95)	7.61 (4.72 to 10.69)	0.36 (0.20 to 0.58)	0.37 (0.21 to 0.58)
**Southeast Asia, East Asia, and Oceania**	11.22 (7.20 to 15.54)	14.54 (8.88 to 20.26)	23.37 (14.99 to 32.36)	30.63 (18.63 to 42.98)	1.28 (0.75 to 1.90)	1.14 (0.66 to 1.84)
East Asia	12.56 (8.15 to 17.68)	16.51 (9.88 to 23.42)	26.53 (17.11 to 37.44)	34.63 (20.61 to 49.27)	1.49 (0.86 to 2.26)	1.31 (0.75 to 2.15)
Oceania	2.35 (1.13 to 3.79)	2.00 (0.95 to 3.43)	4.54 (2.17 to 7.38)	3.82 (1.79 to 6.58)	0.14 (0.08 to 0.22)	0.11 (0.06 to 0.19)
Southeast Asia	7.31 (4.52 to 10.33)	7.71 (4.88 to 10.89)	15.26 (9.49 to 21.65)	16.63 (10.55 to 23.44)	0.72 (0.41 to 1.05)	0.54 (0.33 to 0.81)
**Sub-Saharan Africa**	2.28 (1.27 to 3.31)	2.12 (1.19 to 3.17)	4.30 (2.36 to 6.38)	4.18 (2.33 to 6.23)	0.46 (0.28 to 0.67)	0.41 (0.22 to 0.63)
Central Sub-Saharan Africa	1.50 (0.75 to 2.72)	1.79 (0.79 to 3.25)	3.11 (1.55 to 5.68)	4.12 (1.77 to 7.48)	0.07 (0.04 to 0.12)	0.07 (0.03 to 0.14)
Eastern Sub-Saharan Africa	2.10 (1.19 to 3.14)	2.20 (1.11 to 3.42)	3.91 (2.18 to 5.86)	4.25 (2.14 to 6.65)	0.34 (0.20 to 0.52)	0.44 (0.23 to 0.72)
Southern Sub-Saharan Africa	8.07 (4.37 to 12.16)	6.03 (3.66 to 8.56)	16.80 (8.96 to 25.60)	12.87 (7.77 to 18.36)	1.95 (1.13 to 3.02)	1.51 (0.84 to 2.29)
Western Sub-Saharan Africa	0.70 (0.39 to 1.06)	0.94 (0.52 to 1.50)	1.35 (0.74 to 2.07)	1.91 (1.06 to 3.05)	0.07 (0.04 to 0.12)	0.07 (0.04 to 0.12)

Abbreviations: UI, uncertainty interval; SDI, Socio-demographic Index.

Table S15. Age-standardized disability-adjusted life year rates, per 100,000 population attributable due to tobacco in 1990 and 2021, globally and by regions.

	Both (95% UI)		Males (95% UI)		Females (95% UI)	
	**1990**	**2021**	**1990**	**2021**	**1990**	**2021**
**Global**	6.17 (3.90 to 8.61)	4.37 (2.66 to 6.14)	11.18 (7.11 to 15.60)	8.21 (5.01 to 11.50)	1.82 (1.11 to 2.57)	0.96 (0.57 to 1.43)
**SDI regions**						
High SDI	12.65 (7.91 to 17.80)	7.68 (4.54 to 11.29)	22.06 (13.85 to 31.21)	13.20 (7.77 to 19.33)	5.01 (3.08 to 7.05)	2.75 (1.63 to 4.12)
High-middle SDI	7.78 (5.06 to 10.64)	6.88 (4.32 to 9.50)	16.08 (10.47 to 22.03)	13.79 (8.65 to 19.02)	1.13 (0.70 to 1.59)	0.89 (0.54 to 1.27)
Middle SDI	2.35 (1.53 to 3.21)	2.51 (1.55 to 3.48)	4.48 (2.94 to 6.12)	5.00 (3.09 to 6.93)	0.36 (0.22 to 0.50)	0.26 (0.16 to 0.40)
Low-middle SDI	1.17 (0.73 to 1.63)	1.25 (0.77 to 1.77)	2.15 (1.35 to 2.97)	2.45 (1.52 to 3.44)	0.17 (0.10 to 0.25)	0.15 (0.09 to 0.23)
Low SDI	0.53 (0.31 to 0.77)	0.50 (0.28 to 0.75)	0.96 (0.56 to 1.41)	0.94 (0.52 to 1.40)	0.08 (0.04 to 0.13)	0.08 (0.04 to 0.13)
**Central Europe, Eastern Europe, and Central Asia**	11.24 (7.39 to 15.47)	11.72 (7.40 to 16.13)	25.69 (16.87 to 35.55)	25.41 (16.01 to 35.14)	1.37 (0.87 to 1.93)	1.52 (0.95 to 2.16)
Central Asia	4.38 (2.76 to 6.35)	5.84 (3.75 to 8.25)	10.10 (6.31 to 14.73)	12.98 (8.35 to 18.36)	0.19 (0.11 to 0.28)	0.15 (0.09 to 0.21)
Central Europe	13.59 (8.84 to 18.64)	12.35 (7.66 to 17.67)	26.21 (17.10 to 36.16)	22.99 (14.16 to 32.94)	3.51 (2.22 to 4.97)	3.55 (2.21 to 5.04)
Eastern Europe	11.15 (7.41 to 15.47)	12.81 (8.22 to 17.74)	28.19 (18.68 to 39.34)	30.25 (19.29 to 42.08)	0.52 (0.31 to 0.73)	0.75 (0.46 to 1.06)

**High-income regions**	12.46 (7.76 to 17.53)	7.63 (4.47 to 11.22)	21.83 (13.66 to 30.84)	13.11 (7.64 to 19.26)	4.88 (3.02 to 6.88)	2.80 (1.66 to 4.18)
Australasia	9.35 (5.66 to 13.70)	4.76 (2.69 to 7.40)	14.47 (8.61 to 21.24)	7.81 (4.35 to 12.34)	4.77 (2.95 to 7.02)	1.95 (1.12 to 3.05)
High-income Asia Pacific	6.59 (4.36 to 8.90)	4.26 (2.63 to 6.08)	13.72 (9.04 to 18.42)	8.43 (5.23 to 11.97)	0.93 (0.57 to 1.37)	0.61 (0.35 to 0.95)
High-income North America	14.87 (9.09 to 21.13)	8.66 (5.04 to 13.20)	23.84 (14.50 to 33.85)	13.93 (8.00 to 21.19)	7.38 (4.52 to 10.47)	3.97 (2.27 to 6.05)
Southern Latin America	10.94 (6.78 to 15.49)	10.25 (6.06 to 14.98)	19.43 (11.90 to 27.81)	18.74 (10.87 to 27.85)	3.68 (2.19 to 5.31)	3.09 (1.90 to 4.52)
Western Europe	13.56 (8.50 to 19.02)	8.25 (4.85 to 11.90)	24.38 (15.32 to 34.17)	14.28 (8.36 to 20.60)	5.02 (3.07 to 7.11)	2.93 (1.74 to 4.29)
**Latin America and Caribbean**	3.47 (2.18 to 4.91)	2.72 (1.56 to 4.00)	5.77 (3.66 to 8.12)	4.85 (2.80 to 7.19)	1.37 (0.82 to 1.98)	0.92 (0.51 to 1.39)
Andean Latin America	1.06 (0.61 to 1.60)	1.14 (0.63 to 1.82)	1.91 (1.09 to 2.91)	2.20 (1.23 to 3.55)	0.26 (0.14 to 0.41)	0.17 (0.09 to 0.30)
Caribbean	3.21 (1.96 to 4.71)	2.95 (1.72 to 4.36)	5.41 (3.29 to 7.90)	5.33 (3.07 to 7.81)	1.14 (0.66 to 1.73)	0.81 (0.46 to 1.29)
Central Latin America	2.51 (1.51 to 3.60)	1.69 (1.00 to 2.48)	4.34 (2.61 to 6.24)	3.27 (1.93 to 4.80)	0.82 (0.49 to 1.19)	0.35 (0.20 to 0.52)
Tropical Latin America	4.95 (3.06 to 7.02)	4.03 (2.30 to 6.06)	8.08 (5.10 to 11.37)	6.95 (3.92 to 10.59)	2.17 (1.28 to 3.16)	1.64 (0.91 to 2.54)
**North Africa and Middle East**	3.07 (1.87 to 4.24)	3.25 (2.03 to 4.76)	5.80 (3.54 to 8.01)	6.18 (3.86 to 9.06)	0.26 (0.14 to 0.40)	0.23 (0.12 to 0.34)

**South Asia**	0.94 (0.59 to 1.32)	0.84 (0.52 to 1.19)	1.73 (1.08 to 2.43)	1.66 (1.02 to 2.31)	0.08 (0.04 to 0.12)	0.07 (0.04 to 0.11)
**Southeast Asia, East Asia, and Oceania**	2.67 (1.74 to 3.71)	3.47 (2.08 to 4.86)	5.35 (3.48 to 7.45)	7.05 (4.25 to 9.95)	0.24 (0.14 to 0.36)	0.22 (0.13 to 0.36)
East Asia	2.98 (1.93 to 4.19)	3.99 (2.37 to 5.65)	5.95 (3.86 to 8.38)	8.04 (4.79 to 11.41)	0.27 (0.15 to 0.41)	0.26 (0.15 to 0.43)
Oceania	0.56 (0.27 to 0.90)	0.49 (0.23 to 0.84)	1.06 (0.51 to 1.72)	0.92 (0.43 to 1.58)	0.03 (0.02 to 0.05)	0.03 (0.01 to 0.04)
Southeast Asia	1.73 (1.07 to 2.44)	1.83 (1.16 to 2.57)	3.56 (2.21 to 5.00)	3.85 (2.44 to 5.40)	0.15 (0.08 to 0.21)	0.10 (0.06 to 0.15)
**Sub-Saharan Africa**	0.52 (0.29 to 0.76)	0.49 (0.28 to 0.73)	0.96 (0.53 to 1.42)	0.97 (0.55 to 1.42)	0.10 (0.06 to 0.14)	0.08 (0.05 to 0.13)
Central Sub-Saharan Africa	0.36 (0.18 to 0.63)	0.44 (0.19 to 0.80)	0.74 (0.38 to 1.33)	0.95 (0.42 to 1.75)	0.02 (0.01 to 0.03)	0.02 (0.01 to 0.03)
Eastern Sub-Saharan Africa	0.47 (0.27 to 0.69)	0.49 (0.25 to 0.77)	0.87 (0.49 to 1.29)	0.95 (0.47 to 1.49)	0.07 (0.04 to 0.11)	0.09 (0.05 to 0.14)
Southern Sub-Saharan Africa	1.94 (1.06 to 2.92)	1.47 (0.90 to 2.06)	3.90 (2.06 to 5.94)	3.06 (1.86 to 4.30)	0.46 (0.27 to 0.71)	0.33 (0.19 to 0.49)
Western Sub-Saharan Africa	0.16 (0.09 to 0.24)	0.22 (0.12 to 0.34)	0.30 (0.17 to 0.46)	0.45 (0.25 to 0.70)	0.01 (0.01 to 0.02)	0.02 (0.01 to 0.02)

Abbreviations: DALYs, Disability-Adjusted Life Years; UI, uncertainty interval; SDI, Socio-demographic Index.

Table S16. Age-standardized mortality rates, per 10,000,000 population attributable due to occupational risks in 1990 and 2021, globally and by regions.

	Both (95% UI)		Males (95% UI)		Females (95% UI)	
	1990	2021	1990	2021	1990	2021
**Global**	0.06 (0.01 to 0.11)	0.09 (0.02 to 0.16)	0.09 (0.02 to 0.16)	0.13 (0.03 to 0.24)	0.04 (0.01 to 0.07)	0.05 (0.01 to 0.10)
**SDI regions**						
High SDI	0.07 (0.01 to 0.12)	0.07 (0.02 to 0.13)	0.10 (0.02 to 0.18)	0.10 (0.02 to 0.19)	0.04 (0.01 to 0.08)	0.04 (0.01 to 0.08)
High-middle SDI	0.08 (0.02 to 0.14)	0.11 (0.02 to 0.21)	0.12 (0.03 to 0.21)	0.17 (0.04 to 0.32)	0.04 (0.01 to 0.08)	0.07 (0.01 to 0.13)
Middle SDI	0.06 (0.01 to 0.12)	0.11 (0.02 to 0.20)	0.09 (0.02 to 0.16)	0.15 (0.03 to 0.28)	0.04 (0.01 to 0.07)	0.06 (0.01 to 0.12)
Low-middle SDI	0.03 (0.01 to 0.06)	0.06 (0.01 to 0.11)	0.04 (0.01 to 0.08)	0.09 (0.02 to 0.16)	0.02 (0.00 to 0.03)	0.03 (0.01 to 0.06)
Low SDI	0.03 (0.01 to 0.05)	0.04 (0.01 to 0.08)	0.04 (0.01 to 0.08)	0.06 (0.01 to 0.12)	0.02 (0.00 to 0.03)	0.03 (0.01 to 0.05)
**Central Europe, Eastern Europe, and Central Asia**	0.06 (0.01 to 0.11)	0.08 (0.02 to 0.14)	0.08 (0.02 to 0.15)	0.11 (0.02 to 0.20)	0.04 (0.01 to 0.08)	0.05 (0.01 to 0.10)
Central Asia	0.12 (0.03 to 0.22)	0.16 (0.04 to 0.29)	0.15 (0.03 to 0.29)	0.22 (0.05 to 0.41)	0.09 (0.02 to 0.16)	0.10 (0.02 to 0.18)
Central Europe	0.05 (0.01 to 0.10)	0.07 (0.02 to 0.13)	0.07 (0.02 to 0.13)	0.10 (0.02 to 0.19)	0.04 (0.01 to 0.07)	0.05 (0.01 to 0.09)
Eastern Europe	0.06 (0.01 to 0.10)	0.05 (0.01 to 0.10)	0.07 (0.02 to 0.14)	0.07 (0.02 to 0.13)	0.04 (0.01 to 0.07)	0.04 (0.01 to 0.08)

**High-income regions**	0.07 (0.02 to 0.14)	0.07 (0.02 to 0.13)	0.11 (0.02 to 0.20)	0.11 (0.02 to 0.20)	0.04 (0.01 to 0.08)	0.04 (0.01 to 0.08)
Australasia	0.06 (0.01 to 0.11)	0.06 (0.01 to 0.10)	0.08 (0.02 to 0.15)	0.08 (0.02 to 0.15)	0.04 (0.01 to 0.07)	0.03 (0.01 to 0.06)
High-income Asia Pacific	0.04 (0.01 to 0.07)	0.04 (0.01 to 0.07)	0.06 (0.01 to 0.11)	0.06 (0.01 to 0.11)	0.02 (0.00 to 0.04)	0.02 (0.00 to 0.04)
High-income North America	0.07 (0.02 to 0.14)	0.06 (0.01 to 0.11)	0.10 (0.02 to 0.19)	0.09 (0.02 to 0.16)	0.05 (0.01 to 0.09)	0.03 (0.01 to 0.06)
Southern Latin America	0.35 (0.08 to 0.65)	0.44 (0.10 to 0.81)	0.57 (0.13 to 1.05)	0.68 (0.15 to 1.31)	0.15 (0.03 to 0.29)	0.22 (0.05 to 0.41)
Western Europe	0.06 (0.01 to 0.12)	0.06 (0.01 to 0.11)	0.09 (0.02 to 0.17)	0.09 (0.02 to 0.16)	0.04 (0.01 to 0.07)	0.03 (0.01 to 0.06)
**Latin America and Caribbean**	0.13 (0.03 to 0.23)	0.22 (0.05 to 0.40)	0.17 (0.04 to 0.31)	0.30 (0.07 to 0.56)	0.09 (0.02 to 0.16)	0.14 (0.03 to 0.26)
Andean Latin America	0.16 (0.04 to 0.30)	0.25 (0.05 to 0.47)	0.17 (0.04 to 0.31)	0.30 (0.07 to 0.57)	0.16 (0.03 to 0.29)	0.19 (0.04 to 0.37)
Caribbean	0.09 (0.02 to 0.16)	0.14 (0.03 to 0.25)	0.12 (0.03 to 0.22)	0.18 (0.04 to 0.33)	0.06 (0.01 to 0.11)	0.10 (0.02 to 0.18)
Central Latin America	0.16 (0.03 to 0.29)	0.27 (0.06 to 0.50)	0.21 (0.05 to 0.39)	0.39 (0.08 to 0.72)	0.10 (0.02 to 0.19)	0.17 (0.04 to 0.31)
Tropical Latin America	0.11 (0.02 to 0.19)	0.18 (0.04 to 0.32)	0.15 (0.03 to 0.27)	0.24 (0.05 to 0.45)	0.07 (0.02 to 0.13)	0.11 (0.03 to 0.21)
**North Africa and Middle East**	0.05 (0.01 to 0.10)	0.07 (0.02 to 0.13)	0.09 (0.02 to 0.16)	0.12 (0.03 to 0.23)	0.01 (0.00 to 0.03)	0.02 (0.00 to 0.04)

**South Asia**	0.03 (0.01 to 0.05)	0.04 (0.01 to 0.08)	0.04 (0.01 to 0.08)	0.08 (0.02 to 0.14)	0.01 (0.00 to 0.01)	0.01 (0.00 to 0.02)
**Southeast Asia, East Asia, and Oceania**	0.06 (0.01 to 0.11)	0.11 (0.02 to 0.20)	0.08 (0.02 to 0.16)	0.15 (0.03 to 0.29)	0.04 (0.01 to 0.07)	0.07 (0.01 to 0.13)
East Asia	0.06 (0.01 to 0.12)	0.11 (0.03 to 0.22)	0.09 (0.02 to 0.16)	0.16 (0.04 to 0.31)	0.04 (0.01 to 0.07)	0.07 (0.02 to 0.14)
Oceania	0.01 (0.00 to 0.03)	0.02 (0.00 to 0.04)	0.02 (0.01 to 0.05)	0.03 (0.01 to 0.07)	0.00 (0.00 to 0.00)	0.00 (0.00 to 0.01)
Southeast Asia	0.05 (0.01 to 0.09)	0.09 (0.02 to 0.16)	0.07 (0.02 to 0.13)	0.12 (0.03 to 0.22)	0.03 (0.01 to 0.05)	0.05 (0.01 to 0.10)
**Sub-Saharan Africa**	0.03 (0.01 to 0.06)	0.05 (0.01 to 0.10)	0.04 (0.01 to 0.08)	0.07 (0.02 to 0.13)	0.02 (0.01 to 0.04)	0.03 (0.01 to 0.07)
Central Sub-Saharan Africa	0.03 (0.01 to 0.06)	0.03 (0.01 to 0.07)	0.05 (0.01 to 0.10)	0.05 (0.01 to 0.11)	0.01 (0.00 to 0.03)	0.02 (0.00 to 0.03)
Eastern Sub-Saharan Africa	0.05 (0.01 to 0.09)	0.08 (0.02 to 0.16)	0.06 (0.01 to 0.11)	0.10 (0.02 to 0.20)	0.04 (0.01 to 0.07)	0.07 (0.02 to 0.13)
Southern Sub-Saharan Africa	0.05 (0.01 to 0.09)	0.04 (0.01 to 0.07)	0.07 (0.02 to 0.14)	0.05 (0.01 to 0.10)	0.03 (0.01 to 0.06)	0.02 (0.00 to 0.04)
Western Sub-Saharan Africa	0.02 (0.00 to 0.03)	0.03 (0.01 to 0.06)	0.02 (0.01 to 0.05)	0.04 (0.01 to 0.08)	0.01 (0.00 to 0.02)	0.02 (0.00 to 0.04)

Abbreviations: UI, uncertainty interval; SDI, Socio-demographic Index.

Table S17. Age-standardized disability-adjusted life year rates, per 100,000 population attributable due to occupational risks in 1990 and 2021, globally and by regions.

	Both (95% UI)		Males (95% UI)		Females (95% UI)	
	1990	2021	1990	2021	1990	2021
**Global**	1.97 (0.44 to 3.59)	2.84 (0.62 to 5.22)	2.82 (0.64 to 5.15)	4.13 (0.92 to 7.74)	1.13 (0.25 to 2.07)	1.60 (0.35 to 2.93)
**SDI regions**						
High SDI	2.12 (0.47 to 3.89)	2.19 (0.49 to 4.05)	3.05 (0.67 to 5.61)	3.20 (0.71 to 5.97)	1.26 (0.28 to 2.28)	1.22 (0.27 to 2.23)
High-middle SDI	2.59 (0.58 to 4.72)	3.71 (0.80 to 7.00)	3.87 (0.87 to 7.10)	5.42 (1.16 to 10.31)	1.41 (0.31 to 2.56)	2.08 (0.46 to 3.98)
Middle SDI	2.07 (0.46 to 3.80)	3.44 (0.74 to 6.27)	2.89 (0.66 to 5.36)	4.96 (1.09 to 9.17)	1.22 (0.26 to 2.26)	1.97 (0.41 to 3.63)
Low-middle SDI	0.99 (0.22 to 1.85)	1.88 (0.42 to 3.47)	1.47 (0.33 to 2.77)	2.84 (0.63 to 5.21)	0.49 (0.11 to 0.93)	0.97 (0.21 to 1.80)
Low SDI	0.96 (0.21 to 1.76)	1.42 (0.33 to 2.69)	1.33 (0.29 to 2.47)	2.00 (0.46 to 3.79)	0.57 (0.12 to 1.10)	0.86 (0.20 to 1.65)
**Central Europe, Eastern Europe, and Central Asia**	2.02 (0.45 to 3.73)	2.48 (0.55 to 4.57)	2.77 (0.61 to 5.13)	3.45 (0.75 to 6.48)	1.41 (0.31 to 2.58)	1.65 (0.38 to 3.02)
Central Asia	3.87 (0.86 to 7.13)	5.18 (1.19 to 9.52)	5.04 (1.11 to 9.70)	7.35 (1.66 to 13.64)	2.87 (0.64 to 5.34)	3.28 (0.76 to 5.87)
Central Europe	1.70 (0.37 to 3.17)	2.18 (0.47 to 3.98)	2.35 (0.52 to 4.37)	3.04 (0.66 to 5.63)	1.12 (0.24 to 2.09)	1.38 (0.30 to 2.56)
Eastern Europe	1.84 (0.41 to 3.41)	1.75 (0.38 to 3.30)	2.54 (0.56 to 4.72)	2.33 (0.51 to 4.45)	1.28 (0.29 to 2.37)	1.26 (0.28 to 2.34)

**High-income regions**	2.35<br>(0.52 to 4.30)	2.25<br>(0.50 to 4.12)	3.53<br>(0.78 to 6.46)	3.34<br>(0.73 to 6.13)	1.28<br>(0.29 to 2.33)	1.23<br>(0.27 to 2.29)
Australasia	1.82<br>(0.42 to 3.38)	1.69<br>(0.37 to 3.13)	2.49<br>(0.57 to 4.59)	2.49<br>(0.55 to 4.64)	1.18<br>(0.26 to 2.15)	0.92<br>(0.20 to 1.71)
High-income Asia Pacific	1.15<br>(0.25 to 2.13)	1.11<br>(0.24 to 2.04)	1.81<br>(0.40 to 3.36)	1.67<br>(0.36 to 3.07)	0.56<br>(0.12 to 1.03)	0.56<br>(0.13 to 1.05)
High-income North America	2.39<br>(0.53 to 4.37)	1.87<br>(0.41 to 3.35)	3.37<br>(0.74 to 6.21)	2.76<br>(0.60 to 5.00)	1.50<br>(0.34 to 2.77)	1.04<br>(0.23 to 1.90)
Southern Latin America	11.55<br>(2.58 to 21.61)	13.84<br>(3.04 to 25.60)	19.07<br>(4.27 to 35.34)	21.56<br>(4.76 to 41.30)	4.77<br>(1.02 to 8.91)	6.93<br>(1.52 to 12.94)
Western Europe	1.98<br>(0.44 to 3.61)	1.72<br>(0.38 to 3.22)	2.94<br>(0.64 to 5.42)	2.49<br>(0.54 to 4.68)	1.11<br>(0.25 to 2.02)	0.99<br>(0.22 to 1.82)
**Latin America and Caribbean**	4.08<br>(0.90 to 7.43)	6.82<br>(1.48 to 12.44)	5.40<br>(1.18 to 9.86)	9.44<br>(2.04 to 17.33)	2.83<br>(0.61 to 5.21)	4.50<br>(0.99 to 8.25)
Andean Latin America	5.09<br>(1.11 to 9.45)	7.53<br>(1.61 to 14.44)	5.19<br>(1.12 to 9.68)	9.16<br>(2.03 to 17.30)	4.99<br>(1.09 to 9.35)	6.00<br>(1.16 to 11.49)
Caribbean	2.82<br>(0.62 to 5.17)	4.37<br>(0.96 to 8.12)	3.75<br>(0.85 to 6.87)	5.66<br>(1.24 to 10.38)	1.94<br>(0.42 to 3.55)	3.18<br>(0.68 to 6.06)
Central Latin America	4.93<br>(1.10 to 9.12)	8.55<br>(1.86 to 15.75)	6.68<br>(1.50 to 12.39)	12.19<br>(2.65 to 22.62)	3.28<br>(0.72 to 6.04)	5.34<br>(1.16 to 9.73)
Tropical Latin America	3.41<br>(0.75 to 6.22)	5.45<br>(1.20 to 9.98)	4.71<br>(1.03 to 8.67)	7.56<br>(1.65 to 13.87)	2.21<br>(0.49 to 4.11)	3.59<br>(0.81 to 6.59)
**North Africa and Middle East**	1.68<br>(0.37 to 3.14)	2.29<br>(0.50 to 4.30)	2.88<br>(0.64 to 5.33)	3.85<br>(0.84 to 7.17)	0.42<br>(0.09 to 0.85)	0.63<br>(0.14 to 1.24)

**South Asia**	0.82<br>(0.19 to 1.59)	1.37<br>(0.31 to 2.47)	1.41<br>(0.33 to 2.70)	2.39<br>(0.54 to 4.33)	0.17<br>(0.03 to 0.32)	0.35<br>(0.08 to 0.64)
**Southeast Asia, East Asia, and Oceania**	1.98<br>(0.44 to 3.68)	3.53<br>(0.76 to 6.57)	2.78<br>(0.64 to 5.23)	4.97<br>(1.09 to 9.59)	1.16<br>(0.24 to 2.21)	2.09<br>(0.45 to 3.95)
East Asia	2.12<br>(0.48 to 3.94)	3.76<br>(0.82 to 7.22)	2.93<br>(0.68 to 5.52)	5.32<br>(1.19 to 10.44)	1.26<br>(0.27 to 2.38)	2.19<br>(0.46 to 4.24)
Oceania	0.48<br>(0.11 to 1.01)	0.63<br>(0.15 to 1.32)	0.85<br>(0.19 to 1.80)	1.11<br>(0.25 to 2.35)	0.07<br>(0.02 to 0.14)	0.12<br>(0.03 to 0.23)
Southeast Asia	1.55<br>(0.33 to 2.93)	2.87<br>(0.65 to 5.23)	2.28<br>(0.50 to 4.40)	4.03<br>(0.93 to 7.42)	0.87<br>(0.18 to 1.68)	1.78<br>(0.39 to 3.32)
**Sub-Saharan Africa**	1.10<br>(0.25 to 2.06)	1.59<br>(0.37 to 3.09)	1.41<br>(0.32 to 2.68)	2.14<br>(0.50 to 4.10)	0.78<br>(0.17 to 1.42)	1.09<br>(0.26 to 2.15)
Central Sub-Saharan Africa	1.03<br>(0.24 to 2.00)	1.13<br>(0.27 to 2.36)	1.64<br>(0.37 to 3.31)	1.78<br>(0.38 to 3.80)	0.49<br>(0.11 to 0.97)	0.52<br>(0.12 to 1.17)
Eastern Sub-Saharan Africa	1.52<br>(0.33 to 2.82)	2.62<br>(0.64 to 5.18)	1.81<br>(0.40 to 3.40)	3.25<br>(0.76 to 6.54)	1.22<br>(0.27 to 2.36)	2.02<br>(0.48 to 4.06)
Southern Sub-Saharan Africa	1.65<br>(0.38 to 3.21)	1.24<br>(0.28 to 2.34)	2.37<br>(0.54 to 4.76)	1.79<br>(0.41 to 3.37)	1.04<br>(0.24 to 1.97)	0.79<br>(0.17 to 1.53)
Western Sub-Saharan Africa	0.59<br>(0.13 to 1.11)	0.95<br>(0.21 to 1.86)	0.78<br>(0.17 to 1.49)	1.36<br>(0.29 to 2.67)	0.36<br>(0.08 to 0.70)	0.59<br>(0.13 to 1.14)

Abbreviations: DALYs, Disability-Adjusted Life Years; UI, uncertainty interval; SDI, Socio-demographic Index.

Table S18. Counts of death and disability-adjusted life years for kidney cancer in 2021 and 2050 (reference and improved behavioral and metabolic risks scenarios) for both sexes, globally and by regions.

	Death (95% UI)			DALYs (95% UI)		
	**2021**	**2050 (reference scenario)**	**2050 (improved scenario)**	**2021**	**2050 (reference scenario)**	**2050 (improved scenario)**
**Global**	161,189.01 (150,039.05 to 169,979.20)	323,953.02 (231,579.32 to 429,995.09)	282,192.49 (203,131.64 to 371,248.61)	4,016,362.12 (3,806,832.21 to 4,246,783.03)	6,846,317.48 (4,991,742.18 to 9,090,578.77)	5,583,566.80 (4,106,318.06 to 7,388,235.35)
**Central Europe, Eastern Europe, and Central Asia**	25,332.94 (23,733.58 to 26,835.76)	37,765.86 (25,509.09 to 54,109.39)	32,452.27 (22,462.69 to 45,425.52)	625,250.16 (585,997.46 to 663,510.11)	795,990.50 (541,192.45 to 1,139,008.06)	625,708.26 (432,088.85 to 881,975.40)
**High-income regions**	68,350.87 (61,406.41 to 72,279.87)	108,708.83 (87,297.02 to 130,530.65)	92,133.43 (73,277.83 to 110,820.73)	1,400,271.11 (1,304,885.71 to 1,465,267.72)	1,897,493.19 (1,598,242.22 to 2,235,208.75)	1,490,537.79 (1,242,094.20 to 1,764,553.75)
**Latin America and Caribbean**	13,378.52 (12,300.16 to 14,364.55)	31,498.58 (20,027.35 to 45,463.93)	24,946.88 (16,211.58 to 35,546.73)	369,335.02 (342,657.10 to 398,891.28)	720,859.37 (473,509.26 to 1,026,757.33)	541,765.80 (363,024.10 to 763,241.36)
**North Africa and Middle East**	5465.20 (4821.44 to 6217.80)	20,148.82 (12,335.60 to 29,275.38)	14,665.55 (9178.43 to 21,062.55)	165,276.43 (146,557.66 to 186,215.67)	477,988.45 (306,866.33 to 663,510.57)	318,806.28 (207,038.07 to 441,626.45)
**South Asia**	10,270.17 (9338.93 to 11,219.75)	31,804.44 (19,824.75 to 48,258.76)	30,361.14 (19,309.37 to 45,465.25)	304,759.28 (275,345.09 to 336,310.98)	787,905.65 (507,835.49 to 1,171,886.68)	717,103.66 (469,370.31 to 1,059,417.20)

**Southeast Asia, East Asia, and Oceania**	32,619.04 (27,916.60 to 37,953.31)	73,691.71 (47,197.95 to 104,524.69)	69,835.46 (45,022.89 to 99,104.37)	896,130.33 (770,883.18 to 1,033,411.13)	1,515,739.16 (1,002,777.84 to 2,086,221.88)	1,328,255.26 (888,476.39 to 1,819,560.86)
**Sub-Saharan Africa**	5772.28 (4325.88 to 7136.68)	20,334.79 (13,564.51 to 28,533.62)	17,797.77 (11,915.73 to 24,850.97)	255,339.79 (177,467.00 to 331,342.93)	650,341.16 (411,444.73 to 935,735.80)	561,389.76 (352,662.59 to 811,054.40)

Abbreviations: DALYs, Disability-Adjusted Life Years; UI, uncertainty interval.

Table S19. Age-standardized mortality rate and age-standardized disability-adjusted life year rate (per 100,000 population) for kidney cancer in 2021 and 2050 (reference and improved behavioral and metabolic risks scenarios) for both sexes, globally and by regions.

	ASMR, per 100,000 population (95% UI)			ASDR, per 100,000 population (95% UI)		
	2021	2050 (reference scenario)	2050 (improved scenario)	2021	2050 (reference scenario)	2050 (improved scenario)
**Global**	1.91 (1.77 to 2.02)	1.92 (1.48 to 2.46)	1.42 (1.08 to 1.84)	47.33 (44.76 to 50.07)	47.72 (35.98 to 63.05)	35.86 (26.71 to 48.11)
**Central Europe, Eastern Europe, and Central Asia**	3.88 (3.64 to 4.11)	4.14 (2.92 to 5.72)	2.90 (2.02 to 4.01)	100.14 (93.74 to 106.23)	103.15 (71.03 to 144.93)	71.51 (48.92 to 100.80)
**High-income regions**	2.99 (2.73 to 3.14)	3.21 (2.71 to 3.74)	2.28 (1.92 to 2.64)	70.97 (67.21 to 73.88)	74.55 (63.87 to 86.43)	52.42 (44.80 to 60.96)
**Latin America and Caribbean**	2.17 (1.99 to 2.33)	2.50 (1.70 to 3.50)	1.75 (1.18 to 2.45)	59.08 (54.71 to 63.80)	66.31 (45.08 to 93.35)	46.80 (31.63 to 66.06)
**North Africa and Middle East**	1.25 (1.11 to 1.43)	1.64 (1.09 to 2.28)	0.97 (0.64 to 1.37)	32.81 (29.06 to 36.93)	40.90 (27.63 to 56.39)	24.49 (16.26 to 34.26)
**South Asia**	0.71 (0.64 to 0.78)	0.95 (0.65 to 1.32)	0.82 (0.56 to 1.13)	19.46 (17.60 to 21.45)	25.58 (17.58 to 35.86)	21.97 (15.09 to 30.88)
**Southeast Asia, East Asia, and Oceania**	1.20 (1.03 to 1.39)	1.41 (0.96 to 1.90)	1.13 (0.77 to 1.52)	32.94 (28.53 to 37.73)	37.28 (25.26 to 50.90)	29.77 (20.24 to 40.70)
**Sub-Saharan Africa**	1.02 (0.81 to 1.23)	1.36 (0.96 to 1.88)	1.08 (0.75 to 1.50)	30.80 (22.89 to 38.14)	37.97 (25.31 to 54.35)	30.86 (20.06 to 44.65)

Abbreviations: ASDR, age-standardized disability-adjusted life year rate; ASMR, age-standardized mortality rate; UI, uncertainty interval.

Table S20. Global counts of death and disability-adjusted life years for kidney cancer from 2022 to 2050 (reference and improved behavioral and metabolic risks scenarios) for both sexes.

Year	Death (95% UI)		DALYs (95% UI)	
	Reference scenario	Improved scenario	Reference scenario	Improved scenario
2022	164,488.44 (152,341.66 to 174,111.09)	164,488.44 (152,341.66 to 174,111.09)	4,076,172.08 (3,824,348.34 to 4,320,586.84)	4,076,093.88 (3,824,331.03 to 4,320,491.24)
2023	169,273.06 (156,512.02 to 179,664.39)	167,733.82 (155,084.30 to 178,036.86)	4,169,890.46 (3,907,273.61 to 4,443,357.77)	4,133,246.86 (3,872,672.32 to 4,404,819.26)
2024	174,116.08 (160,145.29 to 185,536.73)	171,140.54 (157,383.43 to 182,376.20)	4,260,707.29 (3,970,170.28 to 4,555,082.06)	4,188,869.84 (3,902,667.88 to 4,479,422.79)
2025	179,181.87 (163,854.76 to 191,563.42)	174,827.78 (159,833.44 to 186,933.66)	4,357,600.93 (4,036,216.60 to 4,669,377.57)	4,250,897.77 (3,936,510.17 to 4,556,530.26)
2026	184,086.54 (167,219.79 to 197,321.74)	178,423.33 (162,002.49 to 191,362.96)	4,451,097.36 (4,099,384.58 to 4,784,715.80)	4,310,214.45 (3,966,221.59 to 4,637,071.81)
2027	189,157.32 (170,404.35 to 203,852.36)	182,226.51 (164,057.11 to 196,409.98)	4,546,087.35 (4,173,001.12 to 4,912,765.51)	4,371,231.71 (4,007,114.45 to 4,728,271.88)
2028	194,534.33 (174,588.11 to 211,229.63)	186,556.07 (167,167.60 to 202,537.67)	4,643,370.96 (4,234,177.65 to 5,044,247.98)	4,438,060.98 (4,042,677.65 to 4,824,591.03)
2029	200,125.73 (178,907.10 to 219,429.32)	191,070.51 (170,654.63 to 209,534.02)	4,743,006.41 (4,276,847.09 to 5,199,904.28)	4,505,887.93 (4,062,429.27 to 4,939,564.04)
2030	205,937.21 (182,631.72 to 228,121.99)	195,769.72 (173,494.97 to 216,766.41)	4,848,030.60 (4,341,560.23 to 5,358,587.55)	4,577,508.23 (4,099,887.03 to 5,061,658.40)
2031	211,522.14 (185,852.58 to 236,462.15)	200,243.60 (175,770.74 to 223,771.64)	4,948,674.71 (4,394,088.81 to 5,498,460.17)	4,644,082.92 (4,124,368.44 to 5,162,344.44)

**2032**	217,174.52<br>(189,330.69 to 244,552.99)	204,757.13<br>(178,189.25 to 230,573.18)	5,049,413.68<br>(4,447,219.63 to 5,665,872.07)	4,709,552.98<br>(4,146,522.53 to 5,284,724.48)
**2033**	223,065.72<br>(192,183.06 to 253,273.91)	209,499.30<br>(180,470.86 to 237,838.44)	5,151,691.25<br>(4,496,483.59 to 5,846,763.20)	4,775,218.54<br>(4,166,243.74 to 5,414,155.70)
**2034**	229,104.85<br>(196,161.16 to 262,354.86)	214,326.94<br>(183,037.73 to 245,374.38)	5,255,366.91<br>(4,533,365.35 to 6,027,943.16)	4,840,354.70<br>(4,180,937.14 to 5,547,997.36)
**2035**	235,286.24<br>(199,605.97 to 271,473.92)	219,229.83<br>(185,710.14 to 252,863.08)	5,363,370.31<br>(4,580,042.10 to 6,201,481.43)	4,907,630.27<br>(4,197,844.23 to 5,669,441.54)
**2036**	241,207.84<br>(202,813.58 to 280,842.03)	223,859.80<br>(187,948.97 to 260,448.99)	5,466,280.96<br>(4,631,147.56 to 6,387,281.46)	4,968,960.50<br>(4,205,273.66 to 5,803,242.47)
**2037**	247,229.08<br>(205,436.57 to 291,072.69)	228,540.12<br>(189,616.71 to 268,700.09)	5,569,957.54<br>(4,683,657.40 to 6,568,299.10)	5,029,490.30<br>(4,221,924.94 to 5,933,956.77)
**2038**	253,448.90<br>(208,306.80 to 301,128.53)	233,336.24<br>(191,514.23 to 276,658.39)	5,675,356.41<br>(4,730,464.68 to 6,755,782.36)	5,088,941.21<br>(4,243,216.46 to 6,060,257.96)
**2039**	259,722.46<br>(211,122.93 to 311,136.52)	238,123.18<br>(193,540.59 to 284,751.87)	5,781,114.84<br>(4,762,996.16 to 6,941,520.57)	5,146,958.68<br>(4,254,346.44 to 6,180,728.34)
**2040**	266,055.01<br>(214,159.74 to 321,448.11)	242,889.85<br>(195,435.42 to 292,914.10)	5,889,378.92<br>(4,805,842.90 to 7,140,342.49)	5,205,083.38<br>(4,261,823.93 to 6,311,223.80)
**2041**	271,989.47<br>(216,881.58 to 331,470.40)	247,247.09<br>(196,638.63 to 300,549.92)	5,990,679.49<br>(4,841,581.22 to 7,339,403.42)	5,255,657.22<br>(4,253,566.67 to 6,438,919.19)
**2042**	277,755.20<br>(218,810.19 to 341,667.59)	251,378.57<br>(197,600.68 to 307,805.45)	6,088,892.70<br>(4,875,293.37 to 7,532,630.23)	5,301,755.20<br>(4,248,153.16 to 6,550,768.82)
**2043**	283,575.31<br>(220,569.68 to 352,204.43)	255,396.08<br>(198,490.35 to 315,481.57)	6,187,046.51<br>(4,893,827.41 to 7,718,922.63)	5,343,259.84<br>(4,236,099.35 to 6,645,678.72)

**2044**	289,420.52 (222,378.68 to 363,273.62)	259,342.34 (199,513.85 to 323,639.95)	6,284,467.90 (4,912,618.33 to 7,930,172.06)	5,382,092.78 (4,219,783.45 to 6,768,940.50)
**2045**	295,304.82 (224,845.28 to 373,850.30)	263,226.49 (200,700.20 to 331,068.80)	6,383,593.46 (4,937,341.76 to 8,118,803.44)	5,420,487.85 (4,207,132.51 to 6,869,265.27)
**2046**	300,899.03 (226,161.54 to 384,716.50)	266,790.00 (201,950.79 to 338,832.71)	6,475,874.42 (4,954,197.09 to 8,314,686.59)	5,451,836.79 (4,186,527.13 to 6,973,150.60)
**2047**	306,581.98 (227,449.96 to 396,055.99)	270,354.86 (202,111.87 to 346,934.23)	6,568,418.67 (4,963,238.26 to 8,503,152.47)	5,481,802.60 (4,164,894.93 to 7,080,961.96)
**2048**	312,361.68 (229,102.39 to 407,206.78)	274,204.47 (202,493.50 to 354,697.15)	6,660,767.85 (4,978,700.74 to 8,695,689.57)	5,513,876.42 (4,142,663.87 to 7,187,416.17)
**2049**	318,117.73 (229,998.66 to 418,968.68)	277,992.47 (202,562.49 to 363,071.75)	6,751,697.94 (4,982,112.35 to 8,882,375.25)	5,543,944.90 (4,116,681.78 to 7,266,734.48)
**2050**	323,953.02 (231,579.32 to 429,995.09)	282,192.49 (203,131.64 to 371,248.61)	6,846,317.48 (4,991,742.18 to 9,090,578.77)	5,583,566.80 (4,106,318.06 to 7,388,235.35)

Abbreviations: DALYs, Disability-Adjusted Life Years; UI, uncertainty interval.

Table S21. Global age-standardized mortality rate and age-standardized disability-adjusted life year rate (per 100,000 population) for kidney cancer from 2022 to 2050 (reference and improved behavioral and metabolic risks scenarios) for both sexes.

Year	ASMR, per 100,000 population (95% UI)		ASDR, per 100,000 population (95% UI)	
	Reference scenario	Improved scenario	Reference scenario	Improved scenario
**2022**	1.92 (1.79 to 2.04)	1.92 (1.79 to 2.04)	47.28 (44.40 to 50.16)	47.28 (44.40 to 50.16)
**2023**	1.92 (1.78 to 2.04)	1.91 (1.77 to 2.02)	47.31 (44.34 to 50.36)	46.89 (43.94 to 49.92)
**2024**	1.92 (1.77 to 2.05)	1.89 (1.74 to 2.01)	47.26 (44.15 to 50.52)	46.43 (43.37 to 49.65)
**2025**	1.92 (1.77 to 2.05)	1.87 (1.72 to 2.00)	47.28 (43.98 to 50.70)	46.04 (42.81 to 49.40)
**2026**	1.92 (1.76 to 2.07)	1.85 (1.69 to 1.99)	47.25 (43.69 to 51.06)	45.60 (42.16 to 49.31)
**2027**	1.92 (1.75 to 2.08)	1.83 (1.67 to 1.98)	47.22 (43.37 to 51.38)	45.17 (41.48 to 49.17)
**2028**	1.92 (1.74 to 2.09)	1.81 (1.64 to 1.98)	47.18 (43.17 to 51.58)	44.77 (40.93 to 48.98)
**2029**	1.92 (1.73 to 2.11)	1.80 (1.62 to 1.97)	47.15 (42.85 to 51.89)	44.37 (40.29 to 48.91)
**2030**	1.92 (1.73 to 2.12)	1.78 (1.60 to 1.97)	47.18 (42.59 to 52.32)	44.00 (39.69 to 48.83)
**2031**	1.92 (1.71 to 2.13)	1.76 (1.57 to 1.96)	47.17 (42.22 to 52.73)	43.61 (39.00 to 48.83)

**2032**	1.92<br>(1.70 to 2.14)	1.75<br>(1.55 to 1.95)	47.15<br>(41.97 to 53.12)	43.20<br>(38.38 to 48.73)
**2033**	1.92<br>(1.69 to 2.15)	1.73<br>(1.52 to 1.95)	47.13<br>(41.63 to 53.68)	42.78<br>(37.70 to 48.84)
**2034**	1.92<br>(1.68 to 2.17)	1.71<br>(1.49 to 1.94)	47.13<br>(41.25 to 54.18)	42.37<br>(36.96 to 48.84)
**2035**	1.92<br>(1.66 to 2.18)	1.69<br>(1.47 to 1.93)	47.17<br>(40.92 to 54.58)	41.99<br>(36.29 to 48.74)
**2036**	1.92<br>(1.65 to 2.20)	1.67<br>(1.44 to 1.93)	47.18<br>(40.53 to 55.12)	41.59<br>(35.61 to 48.77)
**2037**	1.91<br>(1.63 to 2.21)	1.66<br>(1.41 to 1.92)	47.20<br>(40.22 to 55.45)	41.18<br>(34.93 to 48.62)
**2038**	1.91<br>(1.62 to 2.23)	1.64<br>(1.38 to 1.92)	47.23<br>(39.90 to 55.92)	40.77<br>(34.21 to 48.52)
**2039**	1.92<br>(1.61 to 2.25)	1.62<br>(1.36 to 1.91)	47.26<br>(39.51 to 56.49)	40.36<br>(33.55 to 48.52)
**2040**	1.92<br>(1.60 to 2.27)	1.60<br>(1.34 to 1.91)	47.33<br>(39.26 to 57.06)	39.98<br>(33.00 to 48.51)
**2041**	1.92<br>(1.59 to 2.29)	1.59<br>(1.31 to 1.90)	47.37<br>(38.96 to 57.59)	39.57<br>(32.40 to 48.45)
**2042**	1.92<br>(1.58 to 2.31)	1.57<br>(1.28 to 1.90)	47.39<br>(38.57 to 58.15)	39.15<br>(31.75 to 48.40)
**2043**	1.92<br>(1.56 to 2.33)	1.55<br>(1.25 to 1.89)	47.42<br>(38.22 to 58.78)	38.71<br>(31.07 to 48.40)

**2044**	1.91<br>(1.55 to 2.34)	1.53<br>(1.23 to 1.88)	47.44<br>(37.80 to 59.43)	38.27<br>(30.35 to 48.40)
**2045**	1.91<br>(1.54 to 2.36)	1.51<br>(1.20 to 1.87)	47.50<br>(37.48 to 60.05)	37.85<br>(29.72 to 48.37)
**2046**	1.91<br>(1.53 to 2.38)	1.49<br>(1.18 to 1.86)	47.53<br>(37.21 to 60.65)	37.41<br>(29.04 to 48.28)
**2047**	1.91<br>(1.52 to 2.40)	1.47<br>(1.15 to 1.85)	47.56<br>(36.85 to 61.32)	36.98<br>(28.38 to 48.29)
**2048**	1.92<br>(1.50 to 2.42)	1.45<br>(1.13 to 1.85)	47.60<br>(36.54 to 61.99)	36.57<br>(27.79 to 48.32)
**2049**	1.92<br>(1.49 to 2.44)	1.43<br>(1.10 to 1.84)	47.63<br>(36.19 to 62.43)	36.17<br>(27.17 to 48.13)
**2050**	1.92<br>(1.48 to 2.46)	1.42<br>(1.08 to 1.84)	47.72<br>(35.98 to 63.05)	35.86<br>(26.71 to 48.11)

Abbreviations: ASDR, age-standardized disability-adjusted life years rate; ASMR, age-standardized mortality rate; UI, uncertainty interval.

Figure S1. Age-standardized incidence, mortality, and DALYs rates of kidney cancer stratified by sex and age in 2021, globally and by SDI levels. DALYs, disability-adjusted life years; Socio-demographic Index, SDI.

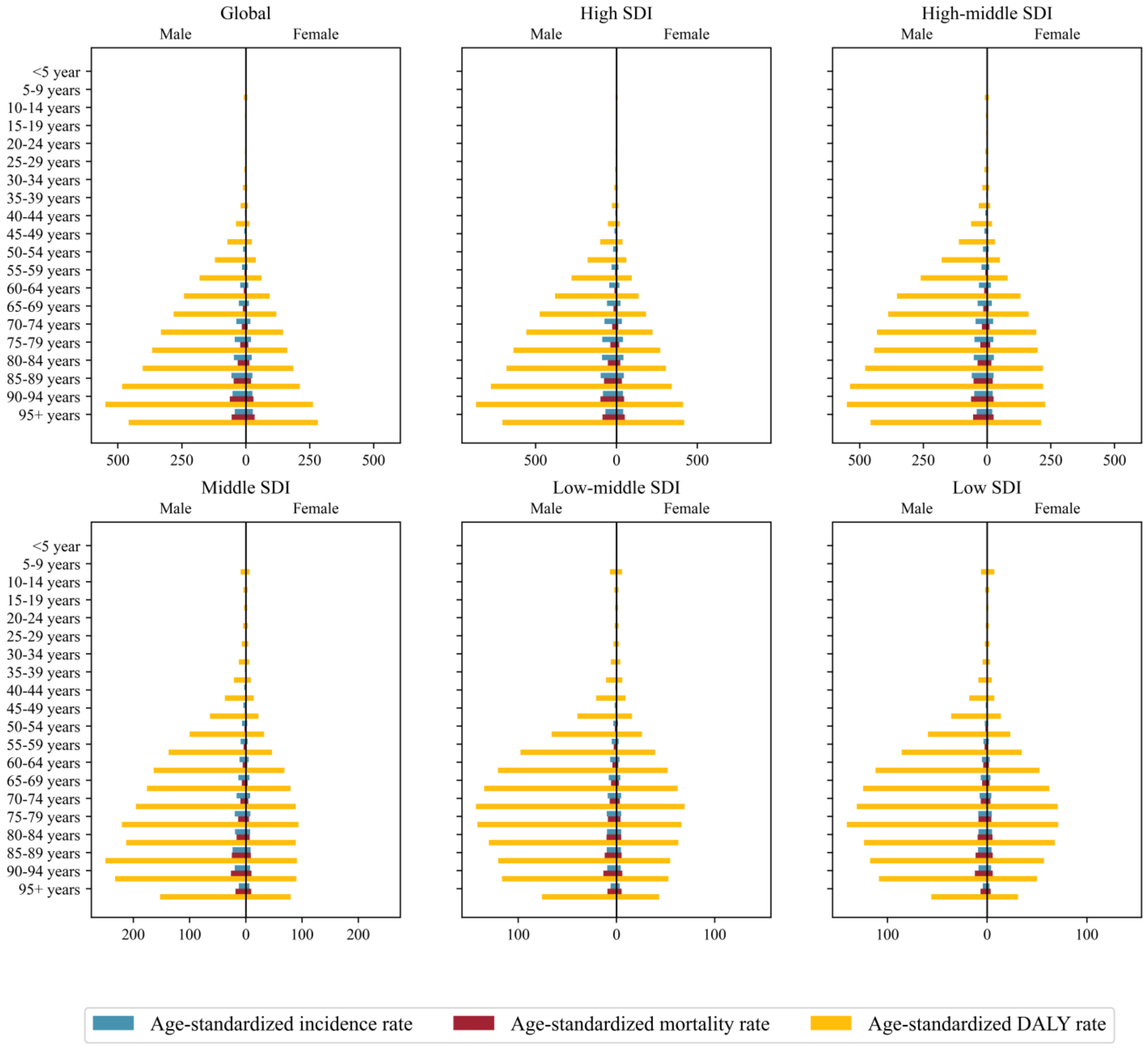

Figure S2. Mortality to incidence ratio of kidney cancer stratified by sex and age in the high SDI region, 2021.

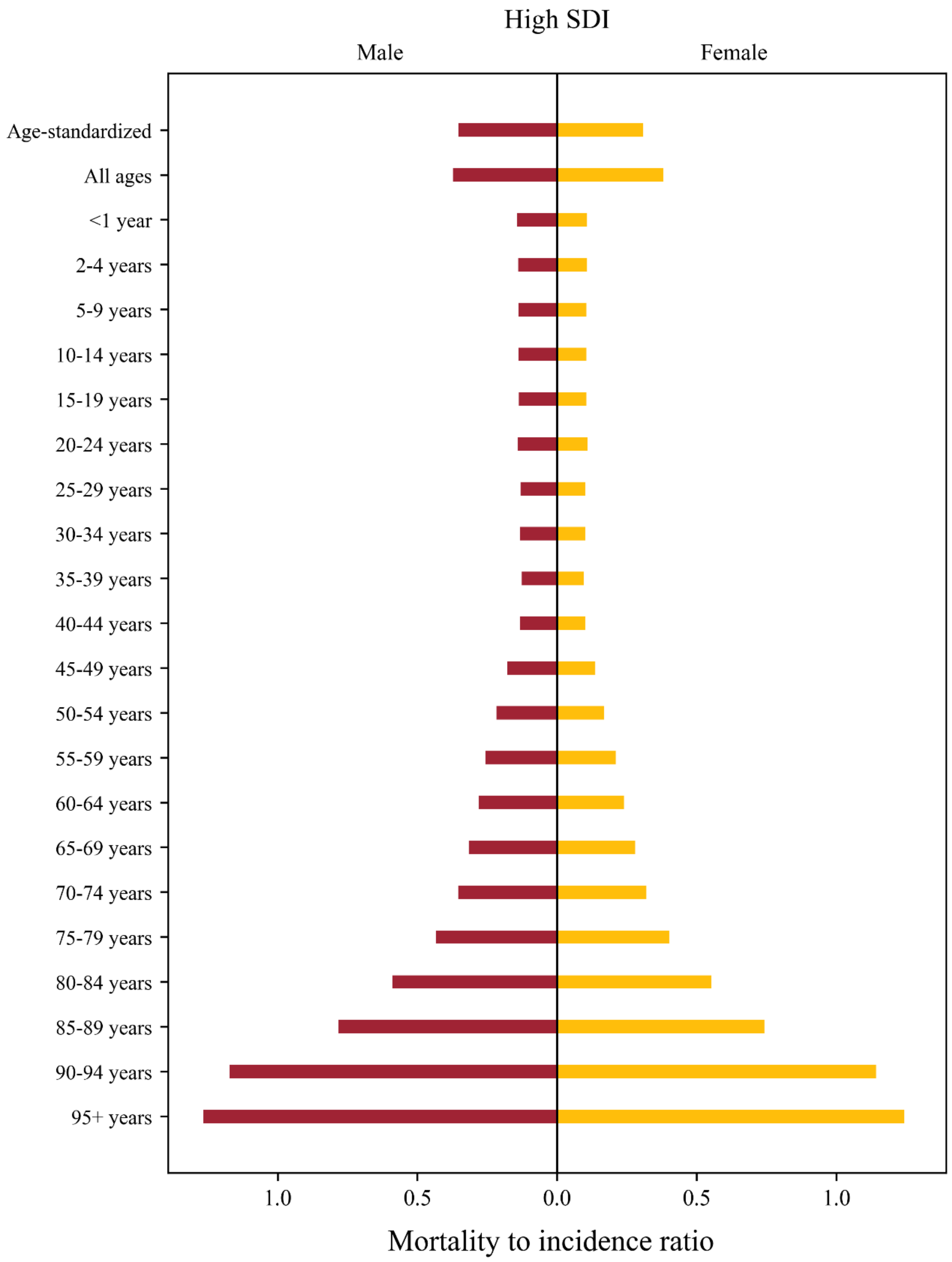

Figure S3. Global mortality to incidence ratio of kidney cancer stratified by sex and age in the high-middle SDI region, 2021.

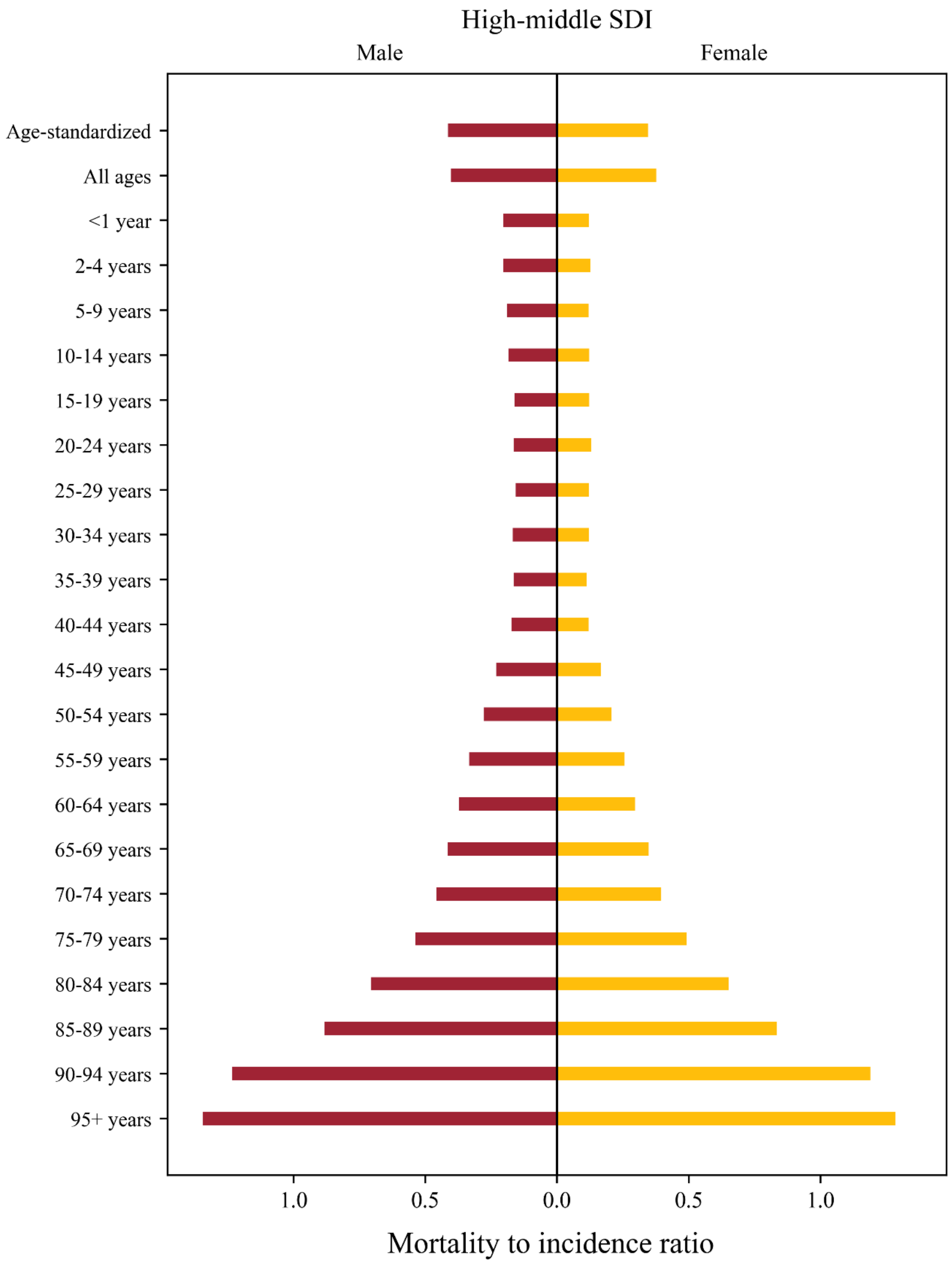

Figure S4. Global mortality to incidence ratio of kidney cancer stratified by sex and age in the middle SDI region, 2021.

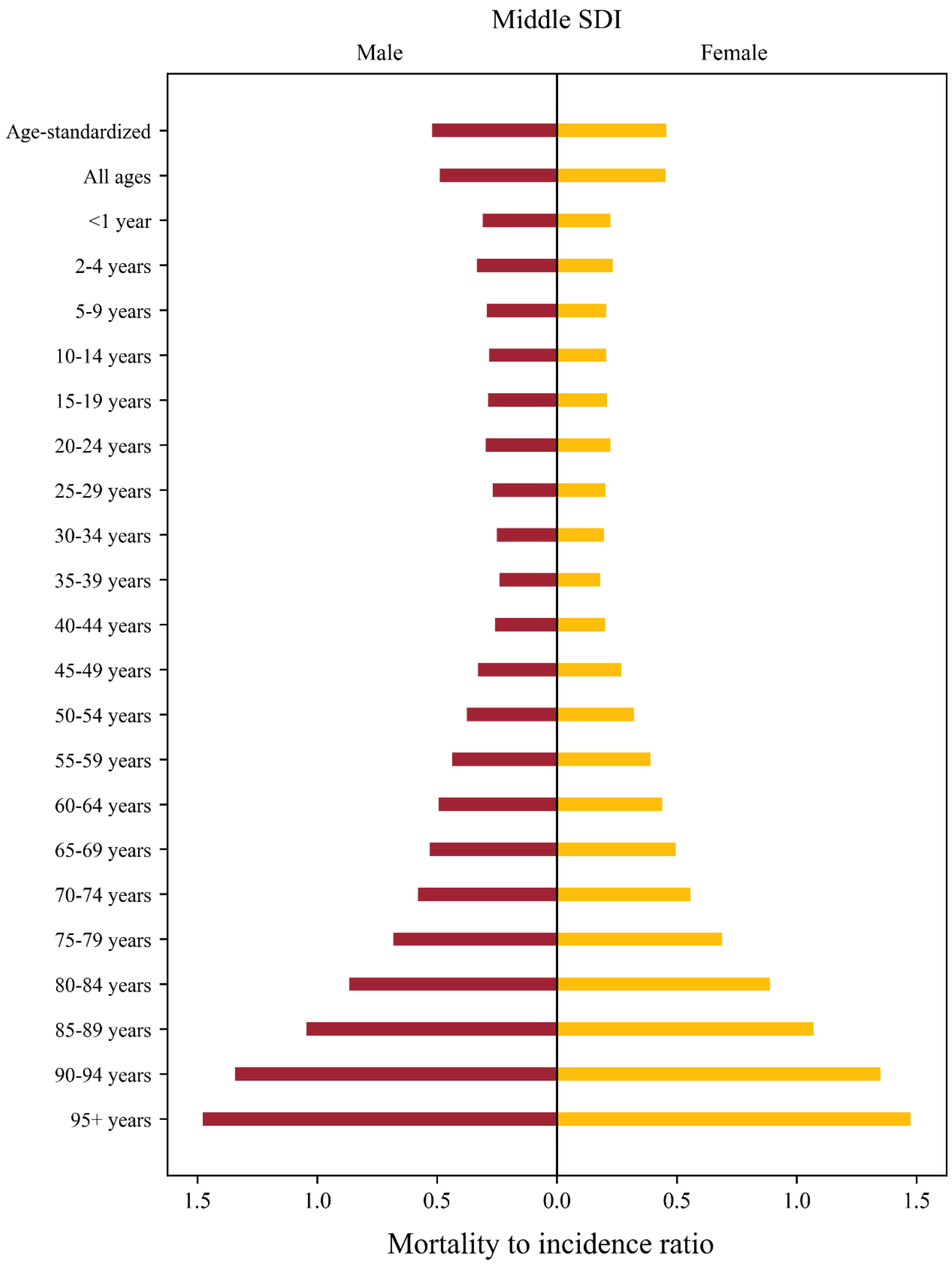

Figure S5. Global mortality to incidence ratio of kidney cancer stratified by sex and age in the low-middle SDI region, 2021.

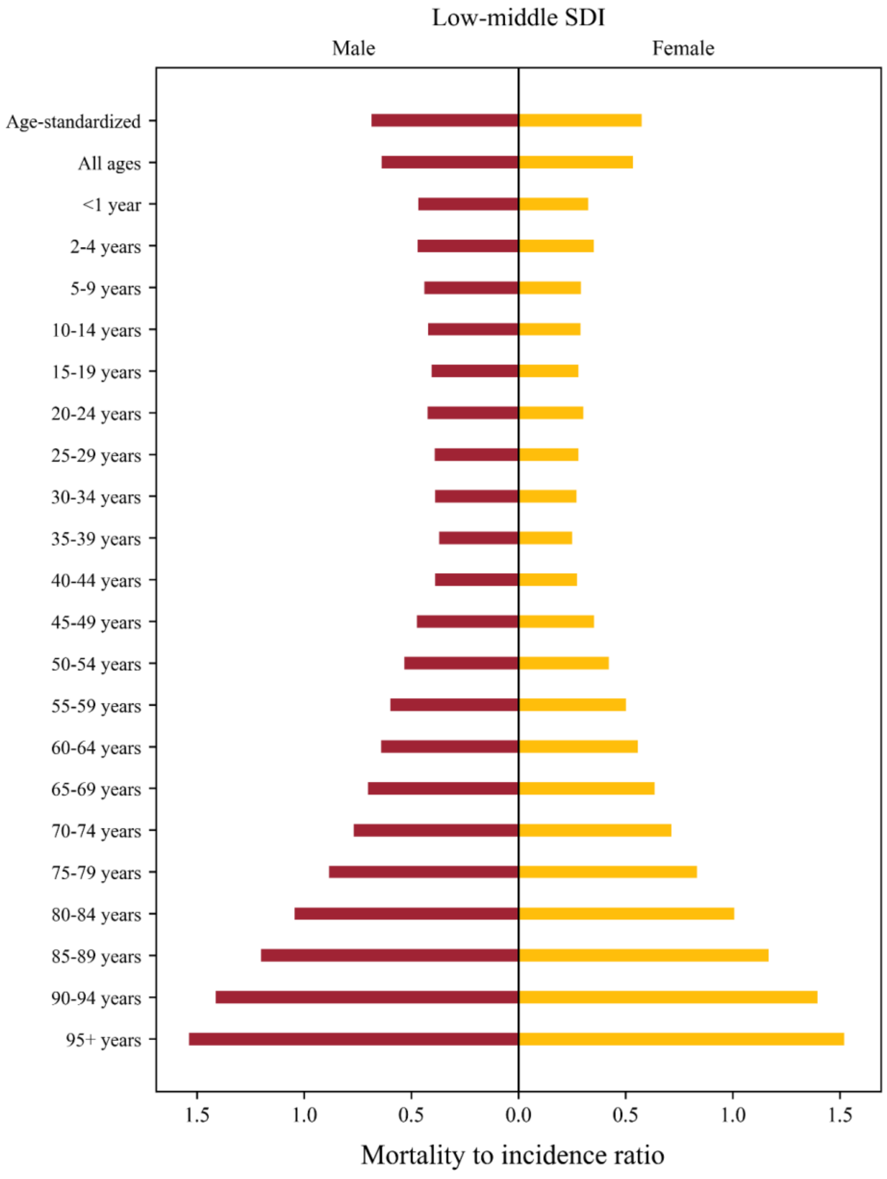

Figure S6. Global mortality to incidence ratio of kidney cancer stratified by sex and age in the low SDI region, 2021.

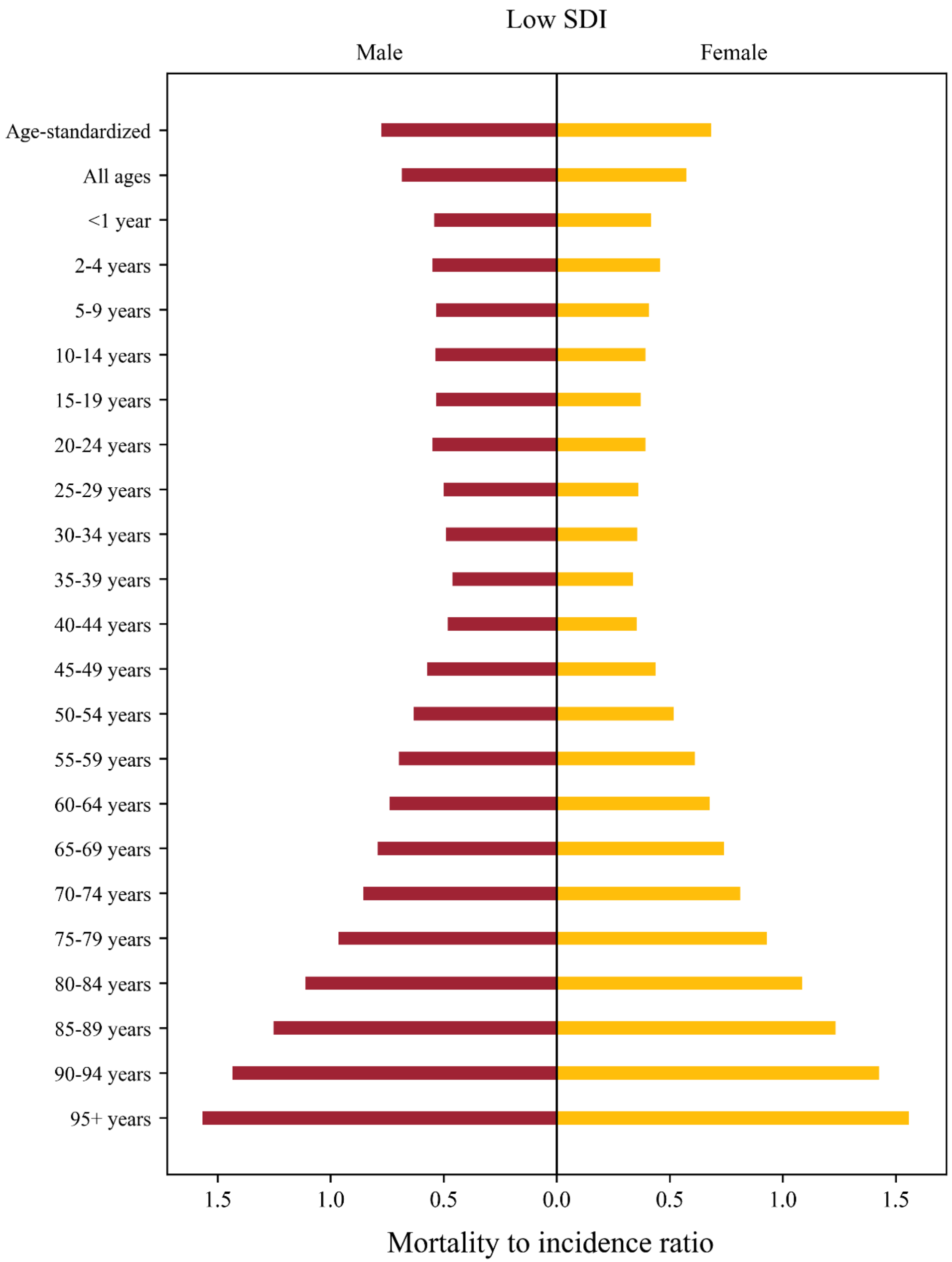

Figure S7. Global distribution of age-standardized (A) incidence, (B) mortality, and (C) DALYs rates of kidney cancer for both sexes in 1990. DALYs, disability-adjusted life years.

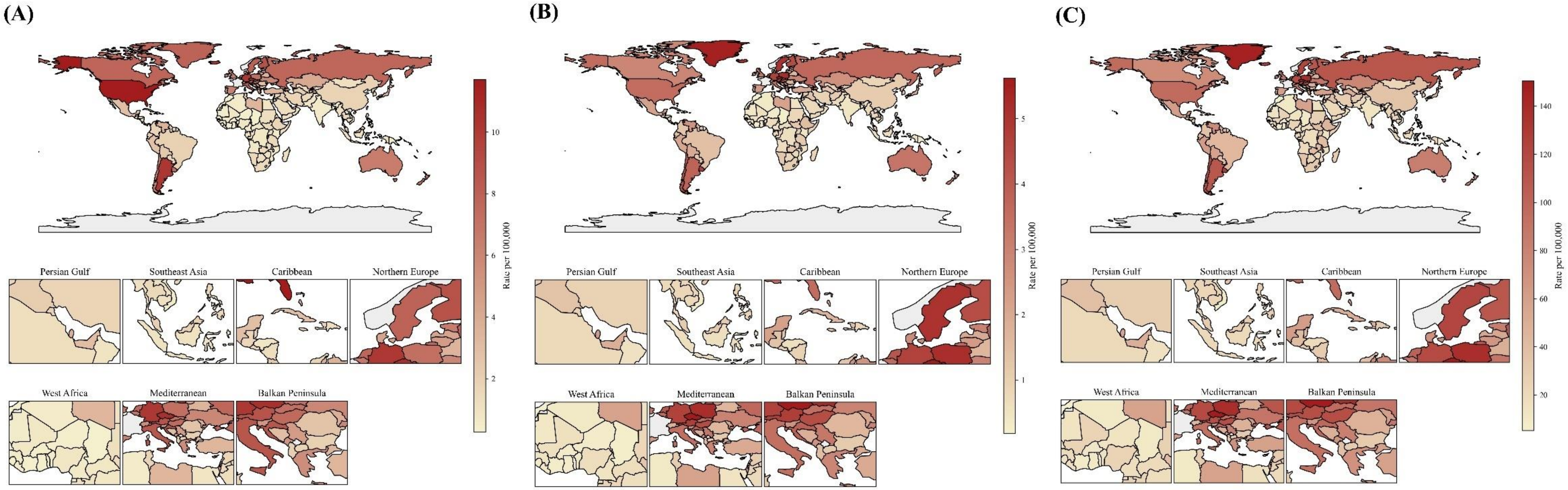

Figure S8. Global distribution of age-standardized (A) incidence, (B) mortality, and (C) DALYs rates of kidney cancer for both sexes in 2021. DALYs, disability-adjusted life years.

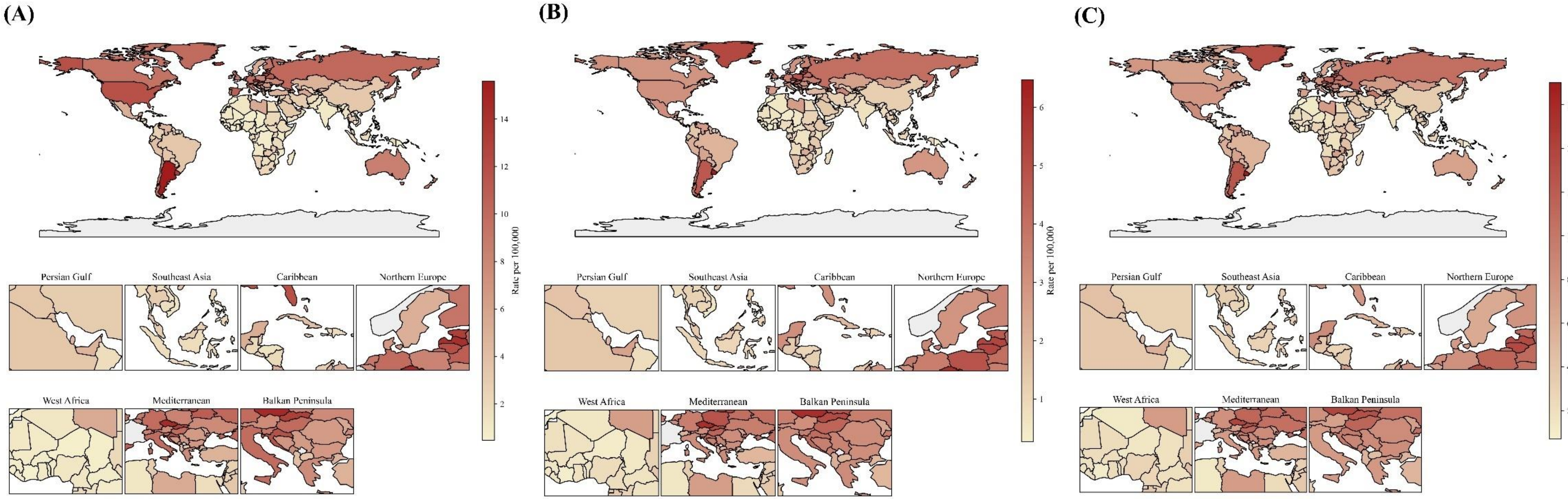

Figure S9. Trends of age-standardized rates for (A) incidence, (B) mortality, and (C) DALYs of kidney cancer for both sexes, 1990–2021, globally and by regions. DALYs, disability-adjusted life years.

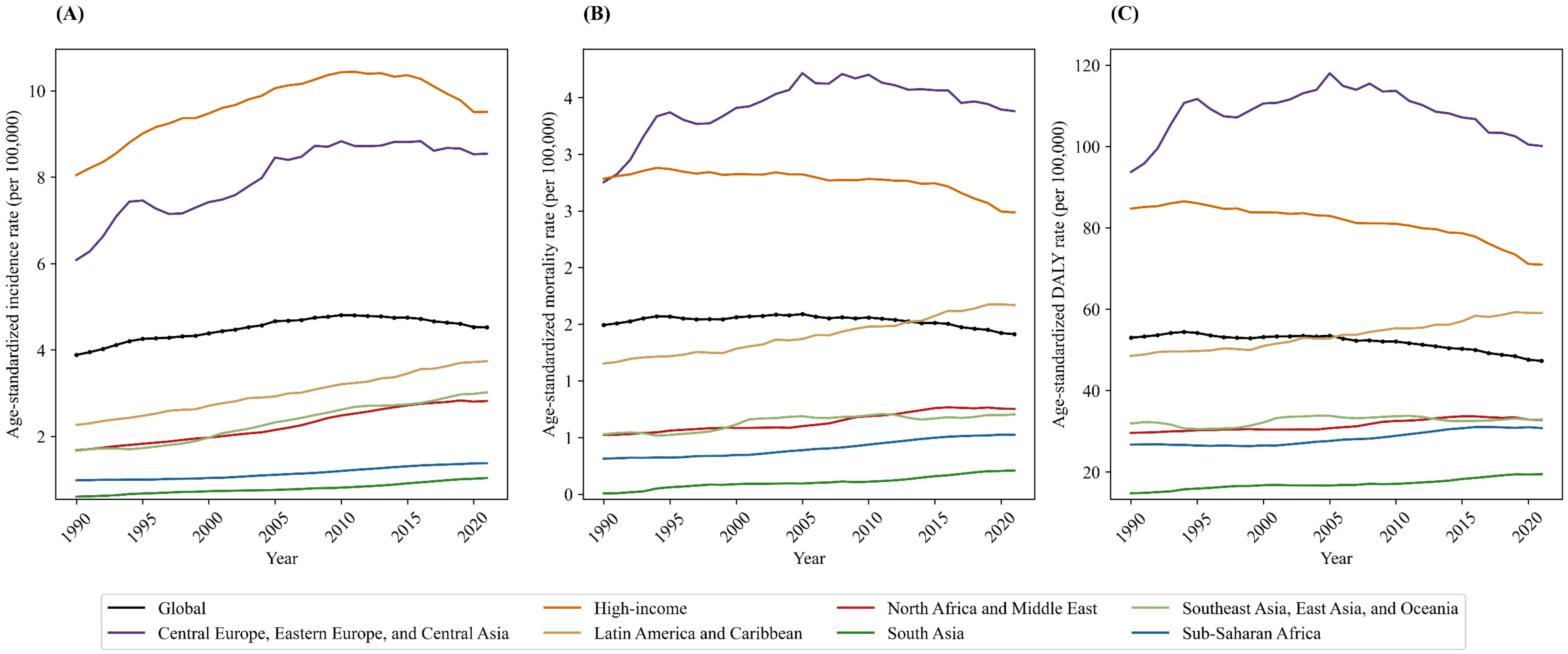

Figure S10. Ranking changes of kidney cancer for both sexes between 1990 and 2021 in age-standardized incidence rate, per 100,000 population (95% uncertainty interval) by regions.

Rank increased | Rank decreased | No change

Rank 1990		Rank 2021		Age-standardized incidence in 2021	Percentage change, 1990-2021
1	High-income North America	1	Southern Latin America	13.44 (12.27 to 14.73)	0.49 (0.33 to 0.66)
2	Southern Latin America	2	High-income North America	11.54 (10.82 to 11.96)	0.02 (-0.02 to 0.05)
3	Western Europe	3	Western Europe	9.74 (9.11 to 10.36)	0.24 (0.16 to 0.32)
4	Eastern Europe	4	Eastern Europe	9.61 (8.82 to 10.40)	0.40 (0.27 to 0.53)
5	Australasia	5	Central Europe	8.63 (7.91 to 9.37)	0.50 (0.36 to 0.64)
6	Central Europe	6	Australasia	8.55 (7.57 to 9.55)	0.29 (0.14 to 0.46)
7	High-income Asia Pacific	7	High-income Asia Pacific	4.58 (4.20 to 4.86)	0.45 (0.35 to 0.52)
8	Central Asia	8	Central Latin America	4.27 (3.82 to 4.76)	0.68 (0.50 to 0.89)
9	Central Latin America	9	Central Asia	3.90 (3.39 to 4.38)	0.40 (0.16 to 0.65)
10	Andean Latin America	10	Andean Latin America	3.56 (2.85 to 4.41)	0.44 (0.13 to 0.81)
11	Caribbean	11	East Asia	3.41 (2.83 to 4.05)	0.88 (0.53 to 1.32)
12	Tropical Latin America	12	Tropical Latin America	3.41 (3.19 to 3.59)	0.74 (0.63 to 0.83)
13	East Asia	13	Caribbean	3.06 (2.69 to 3.44)	0.31 (0.14 to 0.48)
14	North Africa and Middle East	14	North Africa and Middle East	2.82 (2.51 to 3.16)	0.68 (0.44 to 0.94)
15	Southern Sub-Saharan Africa	15	Southern Sub-Saharan Africa	2.13 (1.92 to 2.33)	0.61 (0.40 to 0.89)
16	Southeast Asia	16	Southeast Asia	1.96 (1.74 to 2.20)	0.48 (0.26 to 0.72)
17	Eastern Sub-Saharan Africa	17	Eastern Sub-Saharan Africa	1.72 (1.20 to 2.18)	0.30 (-0.01 to 0.69)
18	Central Sub-Saharan Africa	18	South Asia	1.03 (0.94 to 1.13)	0.71 (0.50 to 0.98)
19	Western Sub-Saharan Africa	19	Western Sub-Saharan Africa	0.98 (0.78 to 1.20)	0.55 (0.28 to 0.93)
20	South Asia	20	Central Sub-Saharan Africa	0.95 (0.58 to 1.48)	0.24 (-0.14 to 0.68)
21	Oceania	21	Oceania	0.64 (0.41 to 0.92)	0.11 (-0.08 to 0.40)

Figure S11. Ranking changes of kidney cancer for both sexes between 1990 and 2021 in age-standardized mortality rate, per 100,000 population (95% uncertainty interval) by regions.

Rank increased | Rank decreased | No change

Rank 1990		Rank 2021		Age-standardized mortality in 2021	Percentage change, 1990-2021
1	Southern Latin America	1	Southern Latin America	4.55 (4.12 to 4.97)	0.15 (0.02 to 0.29)
2	Western Europe	2	Central Europe	4.22 (3.87 to 4.56)	0.18 (0.08 to 0.28)
3	Central Europe	3	Eastern Europe	3.97 (3.64 to 4.31)	0.19 (0.07 to 0.31)
4	High-income North America	4	Western Europe	3.28 (3.01 to 3.50)	-0.10 (-0.17 to -0.05)
5	Eastern Europe	5	High-income North America	3.04 (2.78 to 3.17)	-0.13 (-0.17 to -0.10)
6	Australasia	6	Australasia	2.87 (2.51 to 3.22)	-0.13 (-0.23 to -0.02)
7	Andean Latin America	7	Central Latin America	2.46 (2.21 to 2.73)	0.31 (0.17 to 0.45)
8	Central Latin America	8	Central Asia	2.25 (1.95 to 2.52)	0.27 (0.06 to 0.51)
9	Central Asia	9	Andean Latin America	2.08 (1.67 to 2.56)	0.09 (-0.14 to 0.36)
10	High-income Asia Pacific	10	Tropical Latin America	2.02 (1.87 to 2.13)	0.41 (0.32 to 0.49)
11	Caribbean	11	High-income Asia Pacific	1.89 (1.68 to 2.02)	0.12 (0.04 to 0.17)
12	Tropical Latin America	12	Caribbean	1.67 (1.46 to 1.87)	0.09 (-0.05 to 0.22)
13	East Asia	13	Southern Sub-Saharan Africa	1.45 (1.31 to 1.56)	0.44 (0.24 to 0.72)
14	Eastern Sub-Saharan Africa	14	Eastern Sub-Saharan Africa	1.31 (0.93 to 1.65)	0.17 (-0.09 to 0.49)
15	North Africa and Middle East	15	East Asia	1.27 (1.06 to 1.50)	0.12 (-0.08 to 0.39)
16	Southern Sub-Saharan Africa	16	North Africa and Middle East	1.25 (1.11 to 1.43)	0.22 (0.06 to 0.41)
17	Southeast Asia	17	Southeast Asia	0.97 (0.86 to 1.08)	0.24 (0.06 to 0.44)
18	Central Sub-Saharan Africa	18	Central Sub-Saharan Africa	0.74 (0.45 to 1.18)	0.11 (-0.22 to 0.50)
19	Western Sub-Saharan Africa	19	Western Sub-Saharan Africa	0.72 (0.59 to 0.86)	0.38 (0.15 to 0.68)
20	South Asia	20	South Asia	0.71 (0.64 to 0.78)	0.40 (0.22 to 0.61)
21	Oceania	21	Oceania	0.41 (0.25 to 0.60)	0.10 (-0.10 to 0.39)

Figure S12. Ranking changes of kidney cancer for both sexes between 1990 and 2021 in age-standardized DALYs rate, per 100,000 population (95% uncertainty interval) by regions. DALYs, disability-adjusted life years.

Rank increased | Rank decreased | No change

Rank 1990		Rank 2021		Age-standardized DALY in 2021	Percentage change, 1990-2021
1	Southern Latin America	1	Southern Latin America	120.33 (109.84 to 130.97)	0.07 (-0.04 to 0.19)
2	Eastern Europe	2	Eastern Europe	105.76 (96.57 to 115.64)	0.06 (-0.06 to 0.17)
3	Central Europe	3	Central Europe	102.79 (94.92 to 111.10)	0.08 (-0.01 to 0.18)
4	High-income North America	4	Western Europe	75.82 (71.42 to 80.46)	-0.19 (-0.24 to -0.13)
5	Western Europe	5	High-income North America	74.46 (70.22 to 77.61)	-0.21 (-0.24 to -0.18)
6	Australasia	6	Australasia	67.67 (60.29 to 75.54)	-0.18 (-0.27 to -0.08)
7	Andean Latin America	7	Central Latin America	67.13 (59.97 to 75.34)	0.25 (0.11 to 0.40)
8	Central Asia	8	Central Asia	63.85 (55.23 to 71.86)	0.19 (-0.02 to 0.41)
9	Central Latin America	9	Andean Latin America	55.46 (44.65 to 68.78)	-0.02 (-0.23 to 0.23)
10	Caribbean	10	Tropical Latin America	54.17 (51.10 to 56.95)	0.27 (0.19 to 0.34)
11	Tropical Latin America	11	Caribbean	48.57 (42.24 to 55.71)	0.03 (-0.12 to 0.18)
12	High-income Asia Pacific	12	High-income Asia Pacific	41.24 (38.15 to 43.59)	-0.03 (-0.09 to 0.01)
13	Eastern Sub-Saharan Africa	13	Southern Sub-Saharan Africa	40.36 (36.28 to 44.41)	0.37 (0.20 to 0.57)
14	East Asia	14	Eastern Sub-Saharan Africa	37.82 (25.44 to 49.07)	0.05 (-0.25 to 0.45)
15	North Africa and Middle East	15	East Asia	34.77 (29.00 to 41.18)	-0.02 (-0.21 to 0.24)
16	Southern Sub-Saharan Africa	16	North Africa and Middle East	32.81 (29.06 to 36.93)	0.11 (-0.04 to 0.27)
17	Southeast Asia	17	Southeast Asia	28.03 (24.72 to 31.24)	0.19 (0.03 to 0.36)
18	Western Sub-Saharan Africa	18	Western Sub-Saharan Africa	24.32 (18.31 to 30.31)	0.26 (-0.05 to 0.65)
19	Central Sub-Saharan Africa	19	Central Sub-Saharan Africa	20.48 (12.55 to 31.70)	0.08 (-0.29 to 0.52)
20	South Asia	20	South Asia	19.46 (17.60 to 21.45)	0.31 (0.15 to 0.55)
21	Oceania	21	Oceania	11.50 (7.14 to 16.86)	0.11 (-0.10 to 0.41)

Figure S13. ASMR of kidney cancer attributable to high body mass index, tobacco, and occupational risks in 2021, by (A) both sexes, (B) males, and (C) females, ranked globally and by region. ASMR, age-standardized mortality rate.

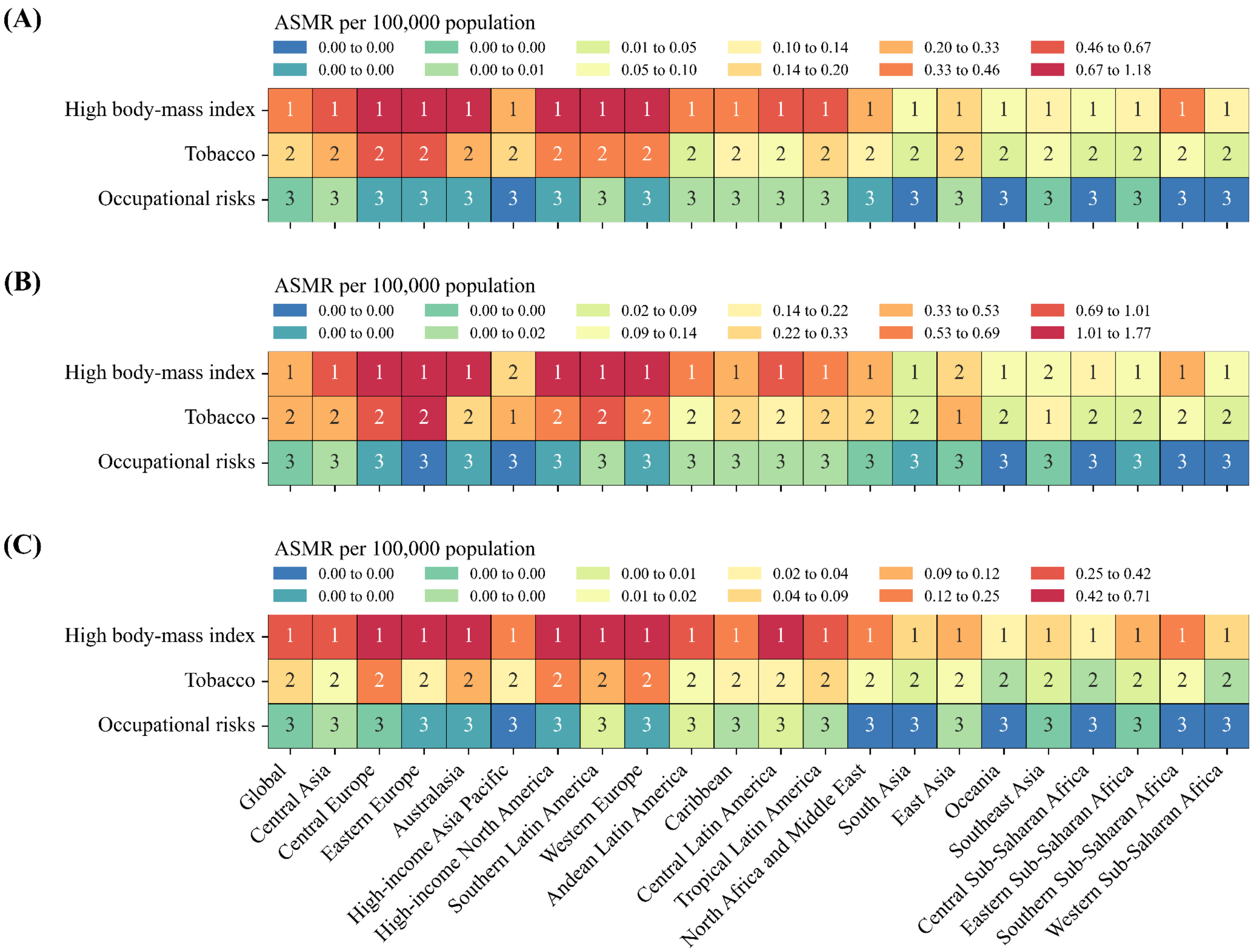

Figure S14. Forecasted age-standardized mortality rates (per 100,000 population) to 2050 under two scenarios (reference and improved behavioral and metabolic risks), globally and by regions. The shaded regions indicate the 95% uncertainty intervals, and the vertically dashed line marks the start of the forecast period in 2022.

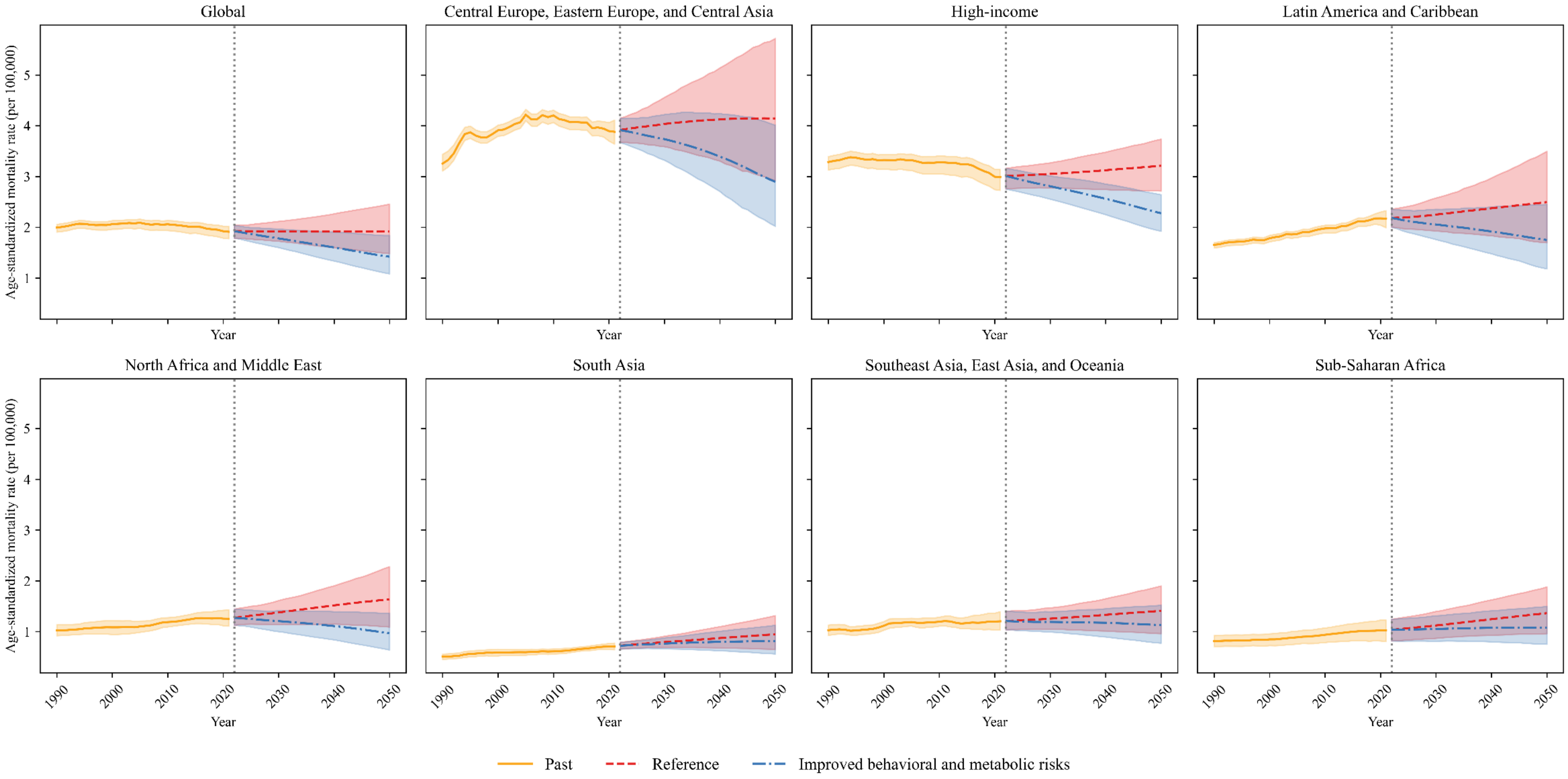

Figure S15. Forecasted ASDR (per 100,000 population) to 2050 under two scenarios (reference and improved behavioral and metabolic risks), by countries in High-income. The shaded regions indicate the 95% uncertainty intervals, and the vertically dashed line marks the start of the forecast period in 2022. ASDR, age-standardized disability-adjusted life years rate.

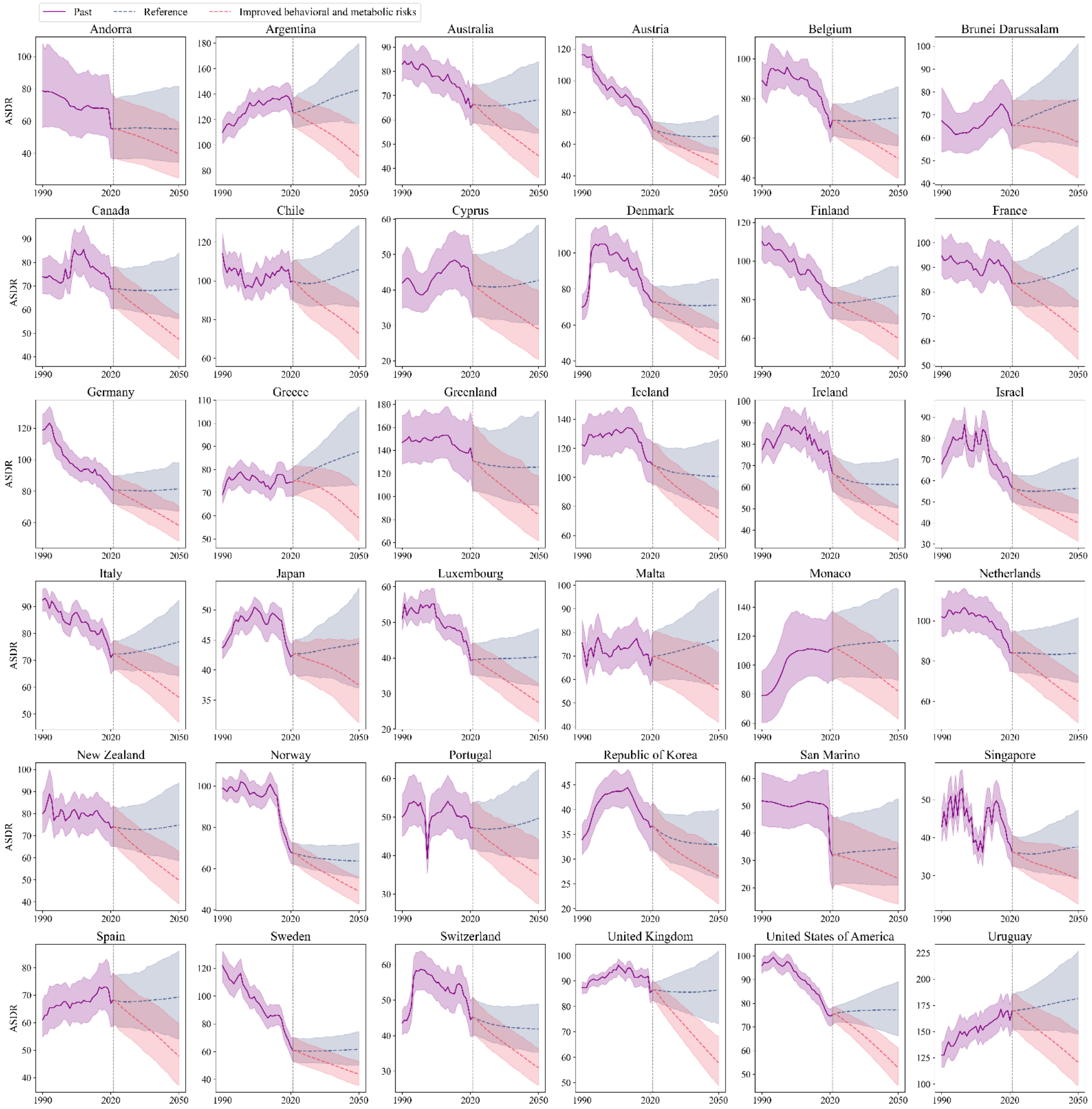

Figure S16. Forecasted ASDR (per 100,000 population) to 2050 under two scenarios (reference and improved behavioral and metabolic risks), by countries in Central Europe, Eastern Europe, and Central Asia. The shaded regions indicate the 95% uncertainty intervals, and the vertically dashed line marks the start of the forecast period in 2022. ASDR, age-standardized disability-adjusted life years rate.

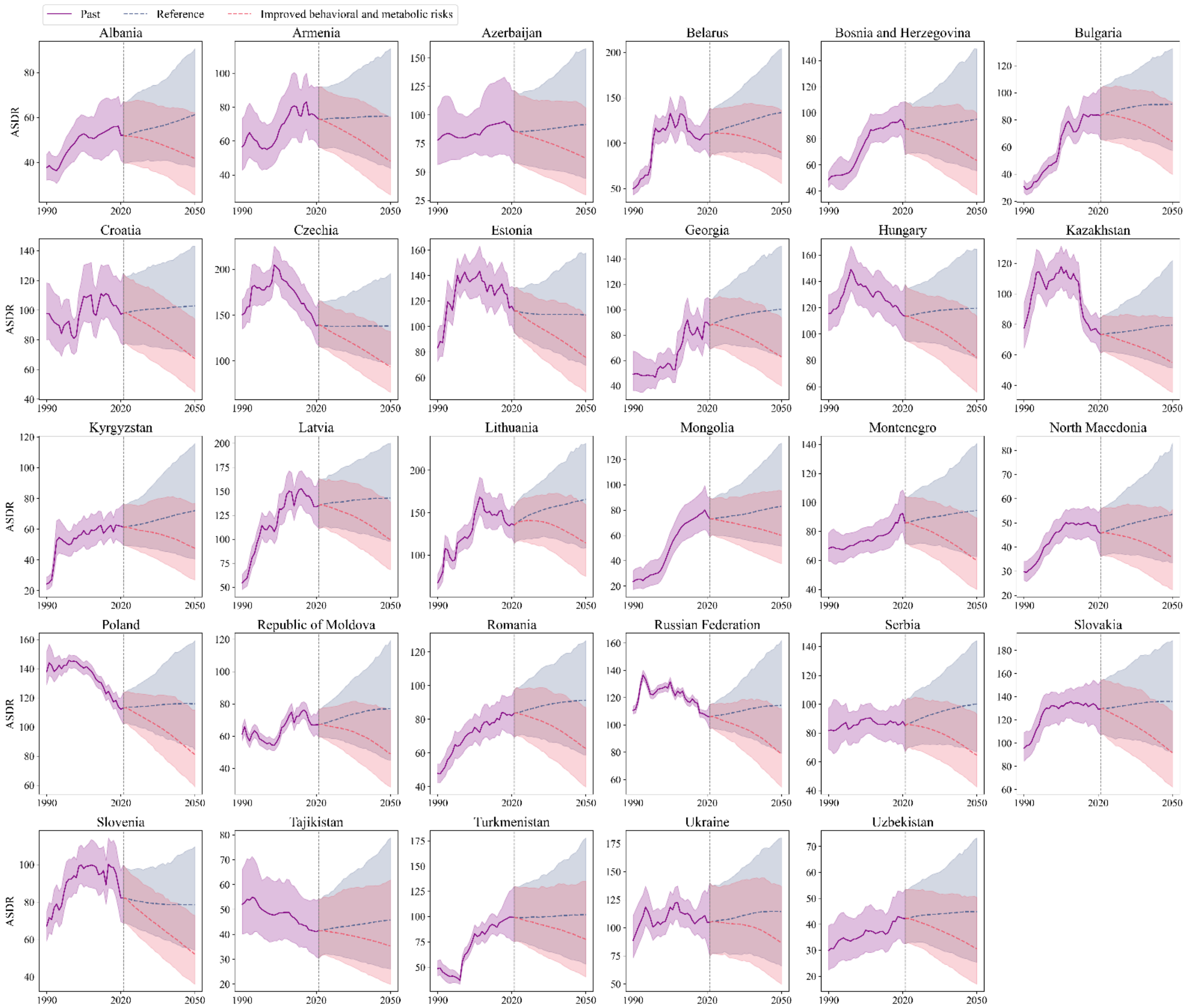

Figure S17. Forecasted ASDR (per 100,000 population) to 2050 under two scenarios (reference and improved behavioral and metabolic risks), by countries in Latin America and Caribbean. The shaded regions indicate the 95% uncertainty intervals, and the vertically dashed line marks the start of the forecast period in 2022. ASDR, age-standardized disability-adjusted life years rate.

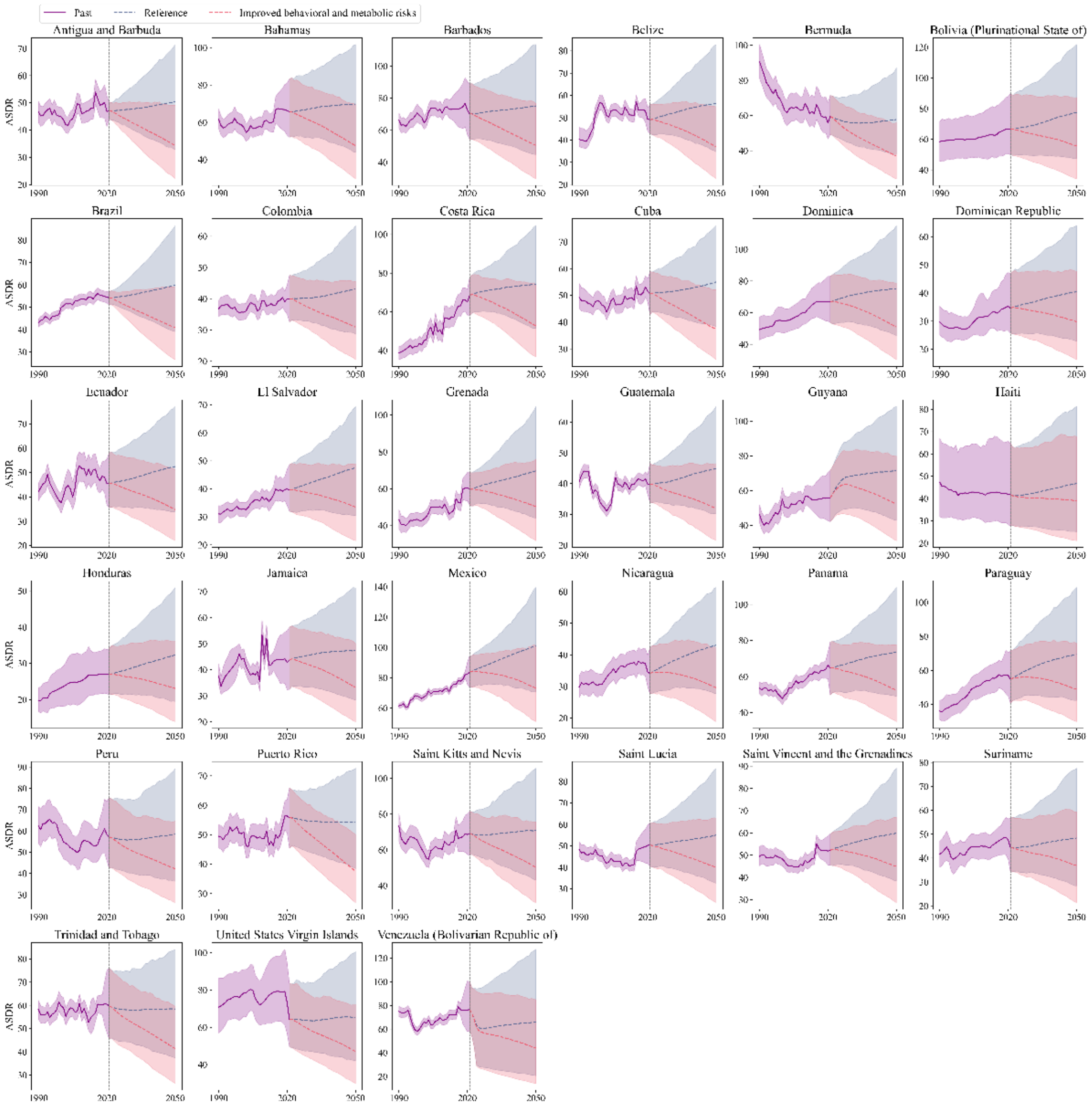

Figure S18. Forecasted ASDR (per 100,000 population) to 2050 under two scenarios (reference and improved behavioral and metabolic risks), by countries in North Africa and Middle East. The shaded regions indicate the 95% uncertainty intervals, and the vertically dashed line marks the start of the forecast period in 2022. ASDR, age-standardized disability-adjusted life years rate.

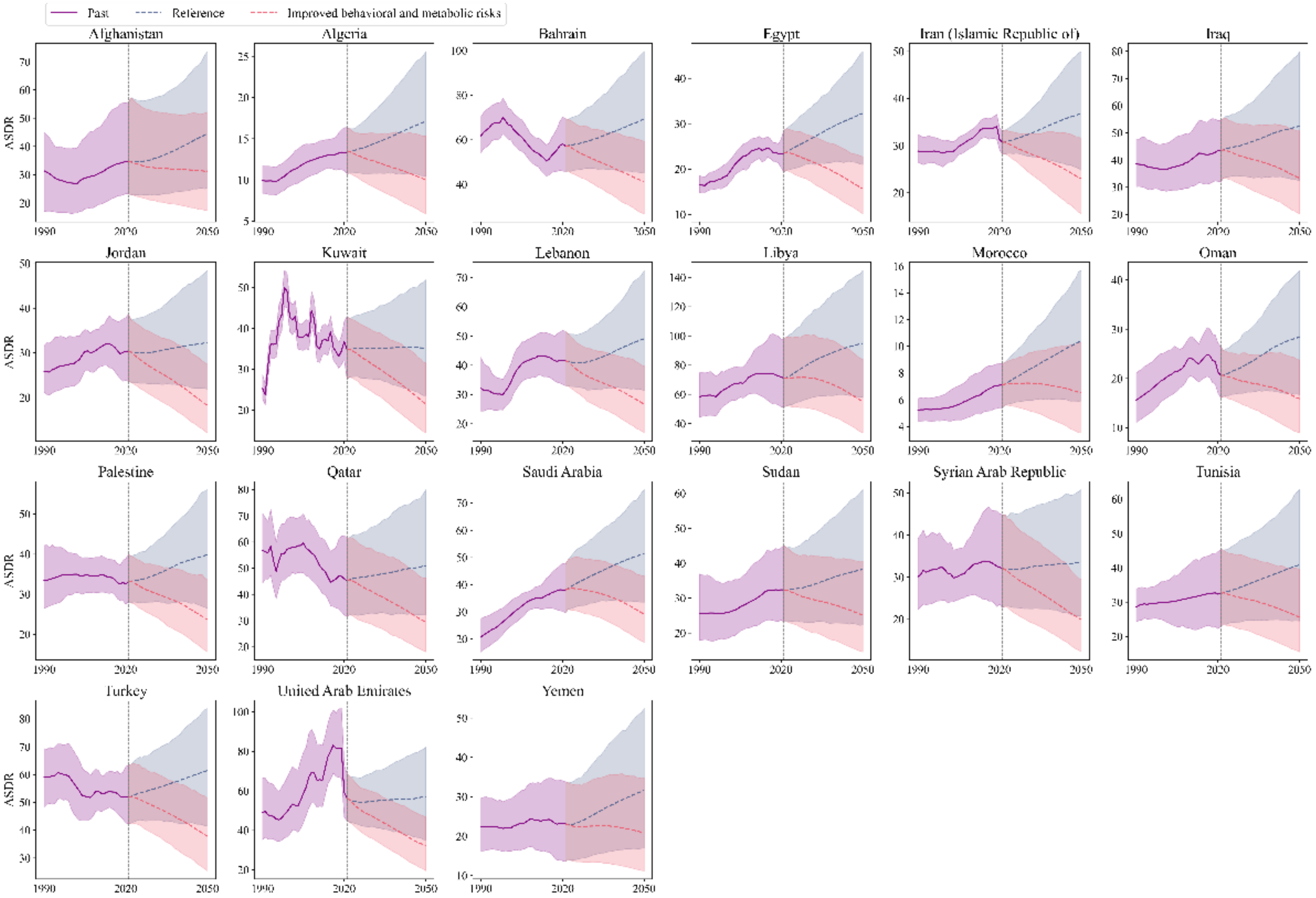

Figure S19. Forecasted ASDR (per 100,000 population) to 2050 under two scenarios (reference and improved behavioral and metabolic risks), by countries in Sub-Saharan Africa. The shaded regions indicate the 95% uncertainty intervals, and the vertically dashed line marks the start of the forecast period in 2022. ASDR, age-standardized disability-adjusted life years rate.

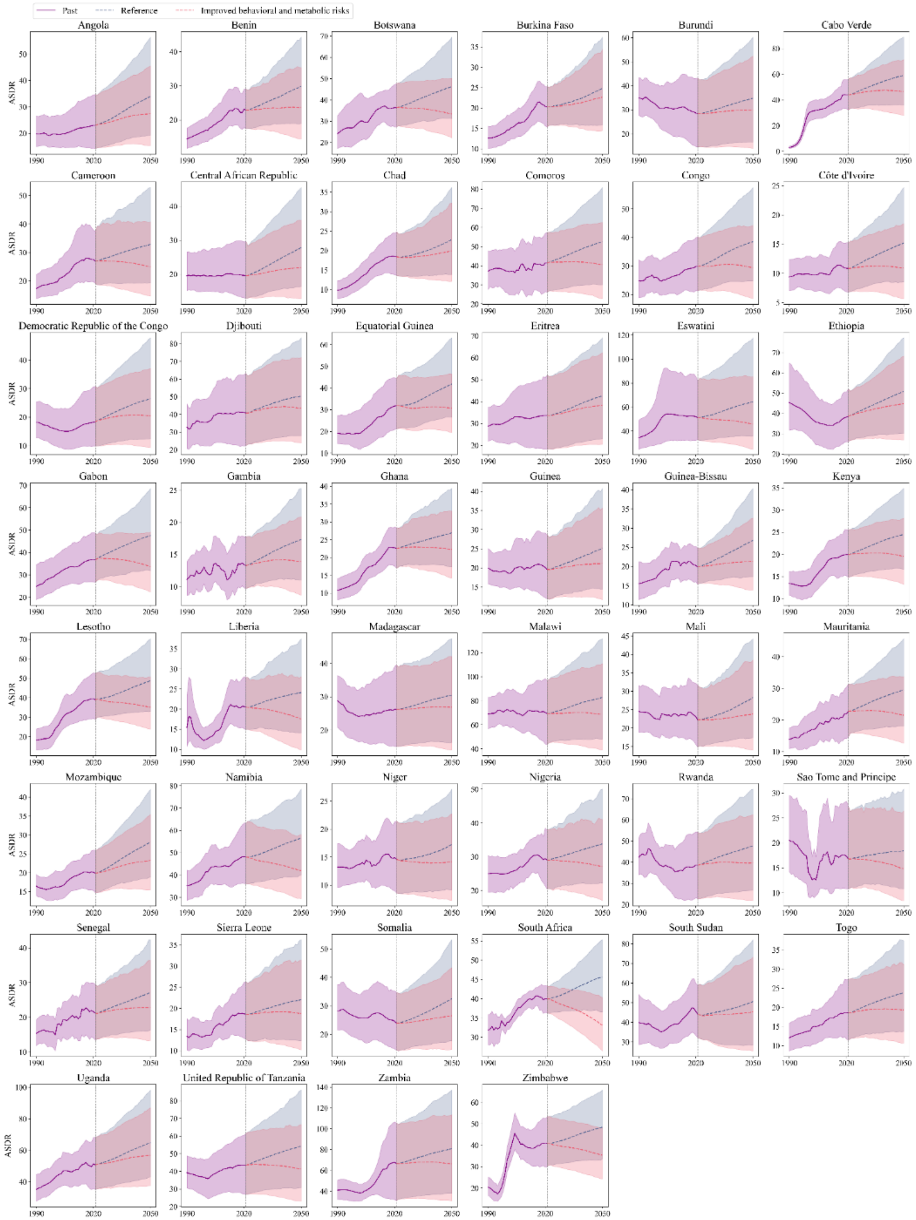

Figure S20. Forecasted ASDR (per 100,000 population) to 2050 under two scenarios (reference and improved behavioral and metabolic risks), by countries in South Asia. The shaded regions indicate the 95% uncertainty intervals, and the vertically dashed line marks the start of the forecast period in 2022. ASDR, age-standardized disability-adjusted life years rate.

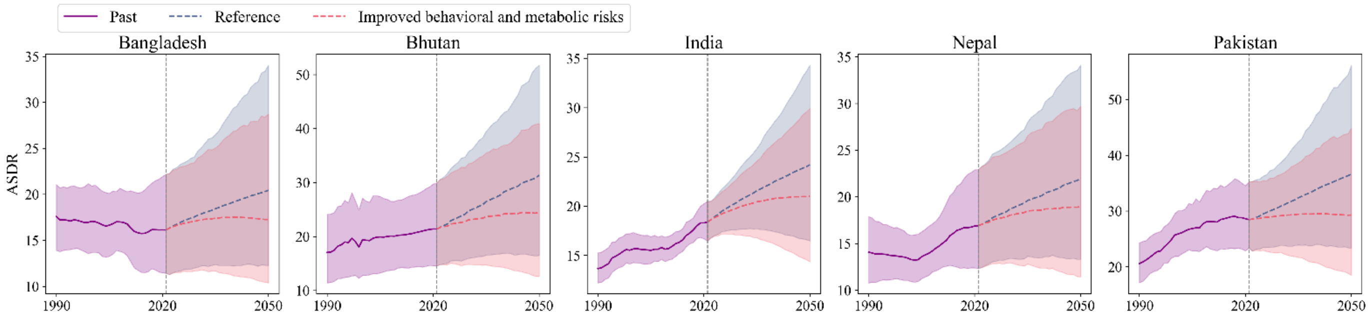

Figure S21. Forecasted ASDR (per 100,000 population) to 2050 under two scenarios (reference and improved behavioral and metabolic risks), by countries in Southeast Asia, East Asia, and Oceania. The shaded regions indicate the 95% uncertainty intervals, and the vertically dashed line marks the start of the forecast period in 2022. ASDR, age-standardized disability-adjusted life years rate.

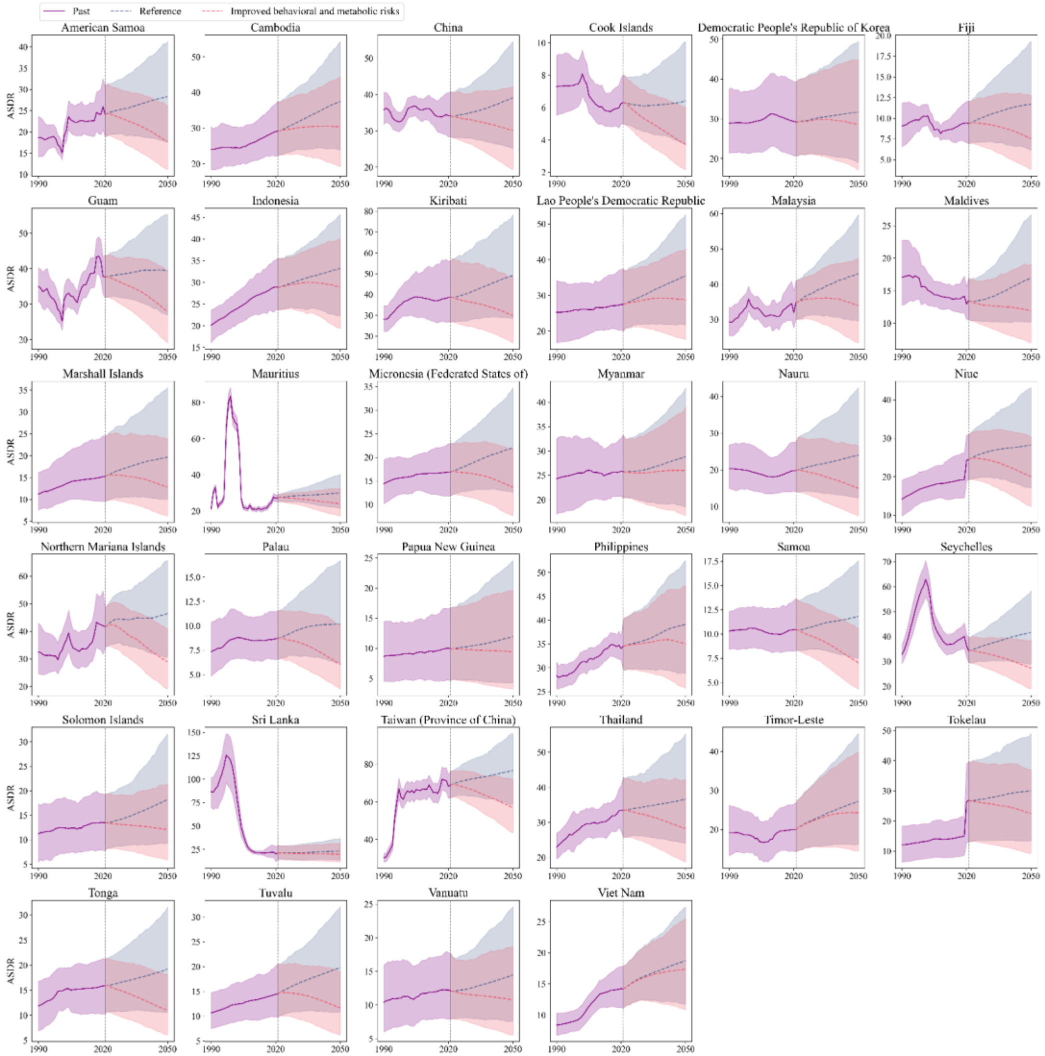

Supplement: Supplementary file 1 [file medi-104-e46059-s001.pdf]
